# Gene expression in the rat brain: High similarity but unique differences between frontomedial-, temporal- and occipital cortex

**DOI:** 10.1186/1471-2202-12-15

**Published:** 2011-01-26

**Authors:** Christine Stansberg, Kari M Ersland, Paul van der Valk, Vidar M Steen

**Affiliations:** 1 Dr E. Martens Research Group for Biological Psychiatry, Department of Clinical Medicine, University of Bergen, Norway; 2 Center for Medical Genetics and Molecular Medicine, Haukeland University Hospital, Bergen, Norway; 3 Department of Pathology, VU University Medical Center, Amsterdam, The Netherlands

## Abstract

**Background:**

The six-layered neocortex of the mammalian brain may appear largely homologous, but is in reality a modular structure of anatomically and functionally distinct areas. However, global gene expression seems to be almost identical across the cerebral cortex and only a few genes have so far been reported to show regional enrichment in specific cortical areas.

**Results:**

In the present study on adult rat brain, we have corroborated the strikingly similar gene expression among cortical areas. However, differential expression analysis has allowed for the identification of 30, 24 and 11 genes enriched in frontomedial -, temporal- or occipital cortex, respectively. A large proportion of these 65 genes appear to be involved in signal transduction, including the ion channel *Fxyd6*, the neuropeptide *Grp *and the nuclear receptor *Rorb*. We also find that the majority of these genes display increased expression levels around birth and show distinct preferences for certain cortical layers and cell types in rodents.

**Conclusions:**

Since specific patterns of expression often are linked to equally specialised biological functions, we propose that these cortex sub-region enriched genes are important for proper functioning of the cortical regions in question.

## Background

The cerebral cortex is the largest and most complex component of the mammalian brain. It is organised into six radial layers with different morphologies and connectivities, whereas in its tangential dimension, it is subdivided into multiple functionally divergent areas, each with characteristic laminar features and electrophysiological and neurochemical properties [1-3].

The properties that distinguish cortical areas emerge gradually during development and various area specific features become evident at different developmental stages [4]. Early arealisation of the developing cerebral cortex is controlled by an interplay between intrinsic genetic factors and extrinsic influences [1,3]. Differential expression across the embryonic cortex is assumed to play a major role in its subdivision into distinct areas, [5,6] and differentially expressed genes are probably involved in genetic control of structural regionalisation and synaptic connectivity [7]. Morphogens secreted from forebrain patterning centres establish gradients of transcription factors along the anterior-posterior and medial-lateral axes early in cortical development [3,4,8], but dramatic changes in gene expression also occur at later stages [9,10]. Cortical "regions" with unclear borders and/or immature laminar structures are not converted to area-specific cytoarchitectures with sharp limits until the post-natal stages [5].

Several studies have identified specific genes with differential expression across cortical areas throughout development [5,6,11-14]. It has however been questioned whether the functional specialisations of adult cortical areas involve expression of a particular set of genes (protomap), or whether functional divergence is primarily achieved by different neural connectivity and/or signal processing within each area (protocortex) [2,7,15]. It is possible that, once area-specific architecture is established, only a handful of genes may be sufficient for its maintenance and execution of its distinct function. Yet another possible cause of differential expression in the adult cortex could be the differential distribution of a particular cell type that specifically expresses the gene of interest [16].

Few differences have so far been found between various adult cortical areas, and gene expression appears more homogenous than could be expected based on their functional divergence [7,17-19]. Global studies of human cortical areas have reported large inter-individual variations [7,18] and transcriptomes of different areas appear more similar within than between individuals [18]. Such inter-individual variation seems to be larger among humans than among chimpanzees [18].

To date, global expression studies of the adult cortex mainly involve humans and primates. Rats should ideally show substantially less inter-individual variation and could therefore pose as a useful model for identifying genes differentially expressed among adult cortical regions. We recently published a study comparing global gene expression in six different regions of the rat brain, including three cortical areas; fronto-medial (FMCx), temporal (TCx) and occipital (OCx), in addition to hippocampus, striatum and cerebellum [20]. In agreement with human and primate studies, global gene expression across rat cortical regions appeared highly homogenous, with average correlations as high as 0.99 between samples from different cortical regions [20].

Genes specifically or highly expressed within a given organ are often closely related to the specialised functions of that particular organ [19-21]. In the present study, we have therefore aimed at identifying genes enriched in rat FMCx, TCx and OCx, by using a combination of microarray-based global gene expression profiling on two different platforms (Applied Biosystems and Illumina), quantitative real-time PCR and data mining.

## Methods

### Animals, tissue dissection and RNA preparation

The present study is a follow up on a previous report comparing global gene expression in different regions in the adult rat brain; including left and right samples of the frontomedial (FMCx), temporal (TCx) and occipital (OCx) cortex from three rats. For independent validation of genes by quantitative real-time PCR, corresponding samples from three additional rats (both left and right sides) were included in the analysis. The animals and procedures for tissue dissection and RNA preparation have been described earlier [20]. The experiments were carried out in accordance with the guidelines of the Norwegian Committee for Experiments on Animals.

### Microarray experiments

We have used data generated for a previous study (using the Applied Biosystems AB1700 Expression Array System, [20]) and have extended this by repeating the analysis of adult rat FMCx, TCx and OCx, with corresponding samples from both left and right hemisphere, on a second microarray platform, the Illumina Whole Genome Expression Bead Chips.

For the Illumina experiments, 250 ng total RNA from each sample was reversely transcribed, amplified and biotin-labelled using the Illumina Total Prep RNA amplification kit (Ambion, UK). Amount and quality of the biotin-labelled cRNA was controlled by both NanoDrop Spectrophotometer and Agilent 2100 Bioanalyzer. 750 ng biotin-labelled cRNA was hybridised to Illumina RatRef-12 Expression Bead Chips (Illumina, USA), according to the manufacturer's instructions. These chips contain 22,523 probes, representing 22,228 rat genes, selected primarily from the NCBI RefSeq database, release 16. Following hybridisation, the Bead Chips were washed and stained with Streptavidin-Cy3. Fluorescent signal detection was performed by the Bead Array Reader and the resulting images were processed by Bead Studio. Signal intensities were imported into J-Express Pro V2.7 software (Molmine, Norway) [22], where inter-array quantile normalisation was performed to minimise the effect of external variables introduced into the data from RNA extraction, -labelling and -hybridisation. The Illumina microarray data are publicly available from ArrayExpress under the accession number [ArrayExpress: E-TABM-1019].

### Quantitative real time PCR analyses

From each sample, 50 ng RNA was reverse transcribed to cDNA using the High Capacity cDNA Archive Kit (Applied Biosystems, Foster City, CA). The final concentrations of the reagents were as follows: 1x TaqMan RT buffer, 5.5 mM MgCl_2_, 2 mM dNTP mixture, 2.5 mM random hexamers, 0.4 U/μl RNase inhibitor and 1.25 U/μl Multiscribe reverse transcriptase in RNase-free water to a total volume of 50 μl. The reaction mix was incubated at 25°C for 10 min (primer annealing), 48°C for 30 min (synthesis) and 95°C for 5 min (enzyme inactivation). The resulting cDNA samples were stored at -20°C. All subsequent real-time PCR experiments were performed on an ABI Prism 7900HT sequence detector system using 384-well plates. The PCR reaction solution contained 0.5 μl 20x TaqMan Gene Expression Assays (Applied Biosystems) and 5 μl 2x TaqMan Universal PCR Master Mix. All PCR reactions contained 1 μl of cDNA reaction mix and RNase-free water to a total volume of 10 μl. The real-time PCR was run as follows: 50°C for 2 min (UNG incubation) and 95°C for 10 min (AmpliTaq Gold activation), followed by 40 cycles of 95°C for 10 s and 60°C for 1 min.

The relative gene expression levels were determined with the comparative ΔΔCt-method [23] and normalised relative to the ribosomal protein P0 gene, *Arbp*, which was found to be the most stable control after testing all samples on the TaqMan Rat Endogenous Control Array (Applied Biosystems) (not shown). Similar results were obtained when normalising relative to other genes, such as *Gapdh*, *Actinb *and *18s*.

### Microarray data analyses

To identify differentially expressed genes, the quantile normalised datasets from J-Express V2.7 were imported into the TM4 Microarray Software suite Multi Experiment Viewer 3.1 (TMeV) (TIGR, US) [24]. Genes showing significant enrichment in FMCx, TCx and OCx, respectively, were identified by a combination of statistical comparisons on data sets resulting from both the AB1700 and Illumina microarray platforms. Using significance analysis of microarrays (SAM) [25], we compared gene expression levels in one cortical region to that of 1) all other brain regions included in the original study; i.e. the other two cortical regions, hippocampus, striatum and cerebellum (only valid for the AB1700 dataset), 2) the other two cortical regions combined and 3) each of the other two cortical regions separately. To reduce false positive findings, the significance threshold was conservatively set to FDR = 0. In addition, each statistically significant gene expression profile was thoroughly explored by manual inspection to exclude genes that failed to display a visually obvious enrichment in one cortical sub-region versus the others.

Functional classification of the resulting lists of regionally enriched genes was performed by the Panther Classification System version 6 (http://www.pantherdb.org) [26,27], as described previously [20].

### Analyses of publicly available data sets

Data sets from previously published analyses describing relevant aspects of the cerebral cortex were downloaded from the Gene Expression Omnibus (GEO) (http://www.ncbi.nlm.nih.gov/geo/) or downloaded from the journal webpage. The data sets analysed are presented in Table 1[5,10,17-19,28-30]. Raw signal intensities were imported into J-Express Pro V2.7 software and quantile normalised. Mapping of probe identifiers across species and microarray platforms was performed using BioMart (http://www.biomart.org). Differential expression among sample groups was tested by ANOVA using a p-value of 0.05 as significance threshold. In addition, expression profiles were generated for all cortical genes that were represented in the respective data sets and manually inspected for trends in gene expression. Housekeeping genes analysed in the Stead data set include the 12 well known endogenous controls represented on the TaqMan Rat Endogenous Control Array (Applied Biosystems) and a set of 27 genes reported to be expressed at the same level in eleven human adult and foetal tissues [31].

**Table 1:** Overview of external data sets analysed in the present study

Description	Reference	Data set	Platform	Replicates	Represented^1^	Differentially expressed^2^
Developing rat brain	Stead JD *et al. *2006	From journal webpage^3^	Affymetrix Rn 230	6	32	29
Mouse neurons, astrocytes, oligo-dendrocytes	Cahoy JD *et al. *2008	GSE9566	Affymetrix Mm 430 2.0	1-6	42	31
Mouse neuronal subtypes	Sugino K *et al. *2005	GSE2882	Affymetrix Mm 430 2.0	3	51	38

Note: Number of the 65 cortex subregion enriched genes ^1^represented by probes in the data set and ^2^showing differential expression among sample groups. ^3^Only pre-processed data available.

In situ hybridisation images for mouse homologues of rat cortical genes were downloaded from the Allen Mouse Brain Atlas (http://www.brain-map.org) [32]. Both sagittal and coronal sections from brain regions corresponding to rat FMCx, TCx and OCx were downloaded (when available) and manually examined at multiple magnification levels. For FMCx and OCx genes, images presented in this study represent sagittal sections near midline (lateral ~0.7-1.4 mm). For TCx genes, coronal sections between Bregma -3.08 and -3.38 are presented.

## Results

### Regionally enriched gene expression in the adult rat frontomedial-, temporal- and occipital cortex

To identify genes with enriched expression in FMCx, TCx or OCx, respectively, we extended the analysis of our previously published AB1700 dataset (ArrayExpress accession number E-BASE-4, [20]). Using significance analysis of microarrays (SAM), we compared gene expression levels in one cortical region to that of all other brain regions included in the original study (hippocampus, striatum and cerebellum) as well as to the other two cortical regions, both combined and separately. We have previously shown that samples originating from the left and right hemispheres of a certain cortical region from the same rat brain are no closer related to each other than to corresponding samples from the other brains [20]. In addition, no systematic difference between samples from left and right hemispheres could be observed [20]. We therefore chose to treat left and right samples from the same cortical region as individual replicates. The number of genes identified as being differentially expressed by each comparison (FDR = 0) is provided in Additional file 1. When all gene lists were combined, a total number of 184 unique genes appeared to be significantly enriched in either FMCx, TCx or OCx (Table 2). To complement and validate these results, the cortical samples were also run on a second microarray platform, the Illumina RatRef-12 BeadArrays. Using the same statistical comparisons and criteria, 345 genes were identified as potentially regionally enriched (Additional file 1).

**Table 2:** Total number of unique genes enriched in each cortical region

Cortical region	Full gene list^1^	Curated gene list^2^
FMCx	56	30
TCx	91	24
OCx	37	11
Total:	184	65

Note: Results from AB1700 ^1^before and ^2^after manual inspection of gene expression profiles and Illumina cross-validation.

**Figure 1:**
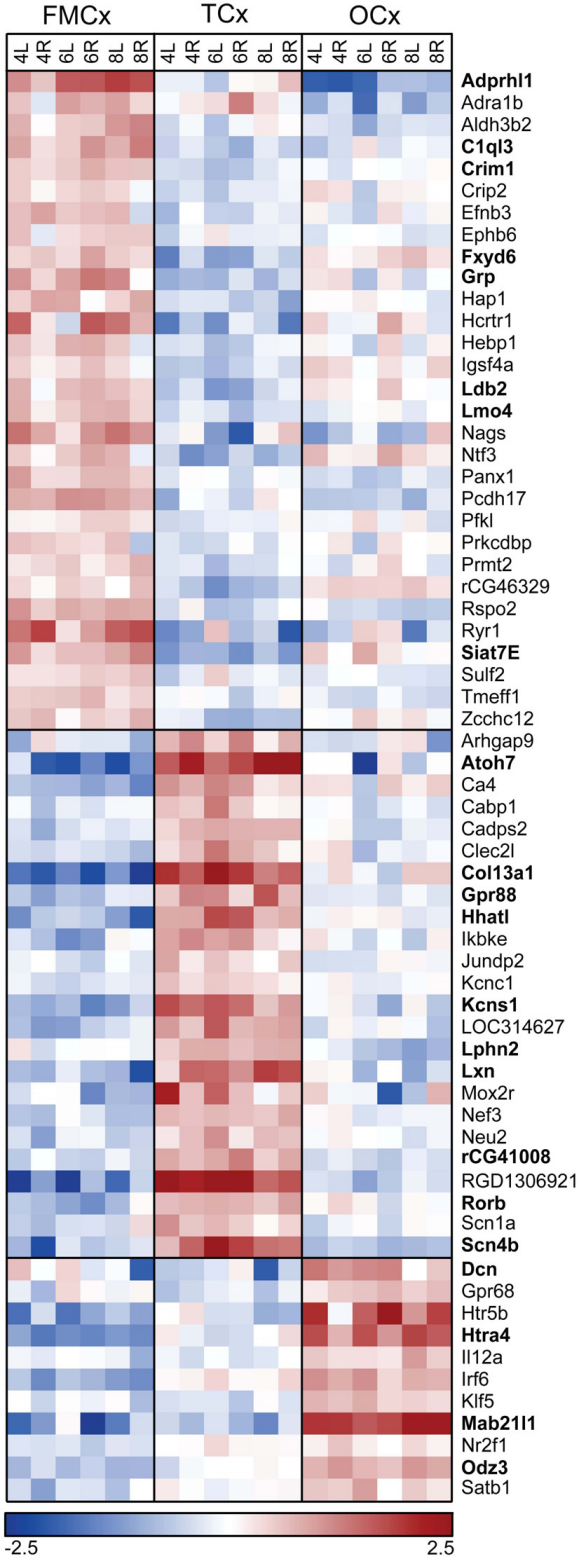
**Genes enriched in rat FMCx, TCx or OCx**. Heat-map illustrating relative expression levels of regionally enriched genes across the six replicates of rat FMCx, TCx and OCx. Each pixel corresponds to a log2-transformed, high-level mean normalised, expression level in a single sample. Red and blue colours indicate expression levels above and below average, respectively. Genes in bold letters were selected for QPCR validation. Numerals (4,6,8) refer to the individual rats. L, left hemisphere; R, right hemisphere. FMCx, fronto-medial cortex; TCx, temporal cortex; OCx, occipital cortex.

**Figure 2:**
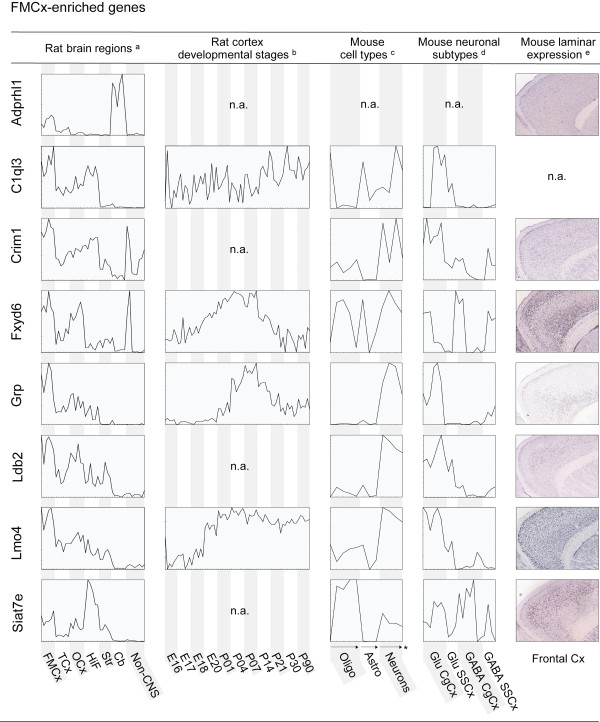
**Spatial and time-dependent expression patterns of genes enriched in FMCx**. Expression profiles for 8 selected genes in rat brain regions, across different stages of the developing cortex, in astrocytes, oligodendrocytes and neurons and in different neuronal subtypes, as well as laminar expression patterns of the regional genes in their corresponding cortical regions. Profiles (a-d) were generated based on microarray data obtained from [20] (a), [10] (b), [28] (c) and [30] (d). Individual samples, including replicates, are placed along the x-axis. The y-axis indicates normalised signal intensities for each gene in each individual sample. Simple profiles are presented for illustration purposes; full profiles with detailed expression levels and sample information are available as additional material. In situ hybridisation images (e) were downloaded from the Allen Mouse Brain Atlas. FMCx, fronto-medial cortex; TCx, temporal cortex; OCx, occipital cortex.; HiF, Hippocampus; Str, Striatum; Cb, cerebellum; Oligo, oligodendrocytes; Astro, astrocytes; Glu, Glutamatergic neurons; GABA, GABAergic neurons; CgCx, cingulated cortex; SSCx, somatosensory cortex; n.a., not available; * arrows indicate increasingly mature cells.

**Figure 3:**
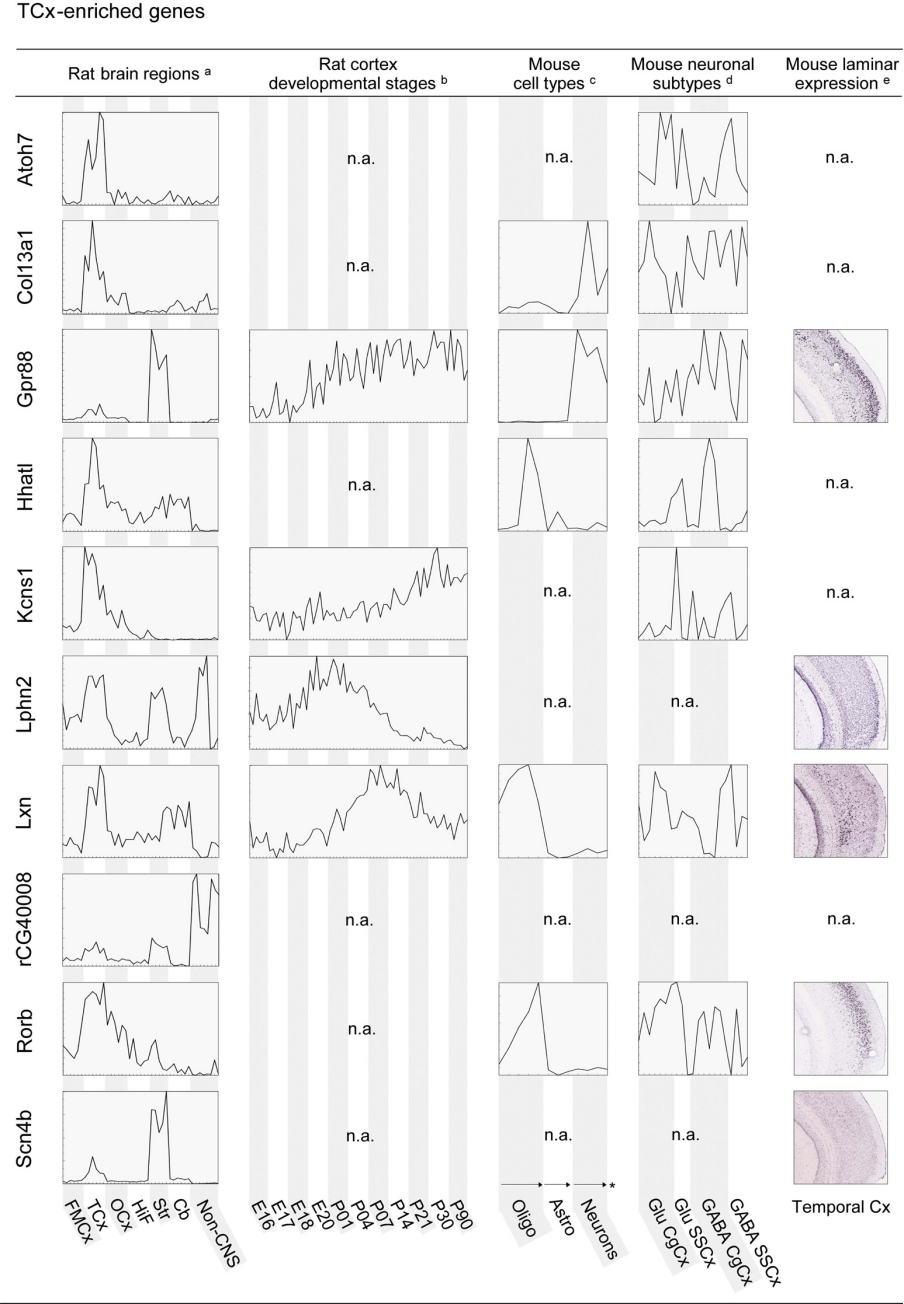
**Spatial and time-dependent expression patterns of genes enriched in TCx**. Expression profiles for 10 selected genes in rat brain regions, across different stages of the developing cortex, in astrocytes, oligodendrocytes and neurons and in different neuronal subtypes, as well as laminar expression patterns of the regional genes in their corresponding cortical regions. Profiles (a-d) were generated based on microarray data obtained from [20] (a), [10] (b), [28] (c) and [30] (d). Individual samples, including replicates, are placed along the x-axis. The y-axis indicates normalised signal intensities for each gene in each individual sample. Simple profiles are presented for illustration purposes; full profiles with detailed expression levels and sample information are available as additional material. In situ hybridisation images (e) were downloaded from the Allen Mouse Brain Atlas. FMCx, fronto-medial cortex; TCx, temporal cortex; OCx, occipital cortex.; HiF, Hippocampus; Str, Striatum; Cb, cerebellum; Oligo, oligodendrocytes; Astro, astrocytes; Glu, Glutamatergic neurons; GABA, GABAergic neurons; CgCx, cingulated cortex; SSCx, somatosensory cortex; n.a., not available; * arrows indicate increasingly mature cells

**Figure 4:**
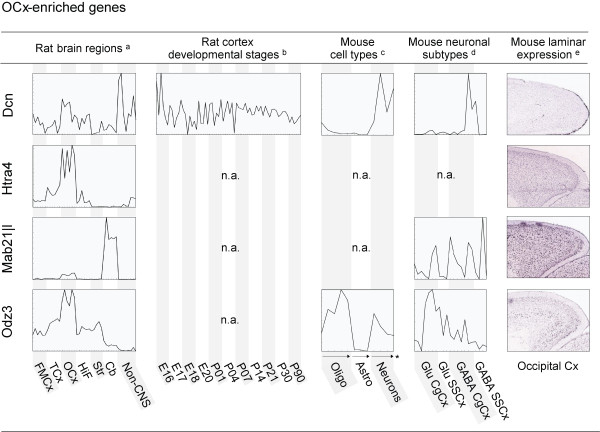
**Spatial and time-dependent expression patterns of genes enriched in OCx**. Expression profiles for 4 selected genes in rat brain regions, across different stages of the developing cortex, in astrocytes, oligodendrocytes and neurons and in different neuronal subtypes, as well as laminar expression patterns of the regional genes in their corresponding cortical regions. Profiles (a-d) were generated based on microarray data obtained from [20] (a), [10] (b), [28] (c) and [30] (d). Individual samples, including replicates, are placed along the x-axis. The y-axis indicates normalised signal intensities for each gene in each individual sample. Simple profiles are presented for illustration purposes; full profiles with detailed expression levels and sample information are available as additional material. In situ hybridisation images (e) were downloaded from the Allen Mouse Brain Atlas. FMCx, fronto-medial cortex; TCx, temporal cortex; OCx, occipital cortex.; HiF, Hippocampus; Str, Striatum; Cb, cerebellum; Oligo, oligodendrocytes; Astro, astrocytes; Glu, Glutamatergic neurons; GABA, GABAergic neurons; CgCx, cingulated cortex; SSCx, somatosensory cortex; n.a., not available; * arrows indicate increasingly mature cells

After strict manual inspection of all FDR = 0 selected expression profiles, a total of 65 genes were ultimately determined as being markedly enriched in either FMCx, TCx or OCx, of which 54 were supported in both data sets. Nine of the AB1700-generated genes could not be confirmed in the Illumina data, either due to non-representation on the Illumina RatRef-12 array (*Aldh3b2, C1ql3, rCG46329, rCG41008, RGD1306921 *and *Mab21|l*) or lack of significant enrichment (*Lmo4*, *Gpr68 *and *Siat7e*). Figure 1 illustrates the expression levels of the 65 genes in all cortical samples, of which 30 were enriched in FMCx, 24 in TCx and 11 in OCx (Table 2). Gene expression profiles of selected genes are presented in Figure 2 (FMCx), Figure 3 (TCx) and Figure 4 (OCx), whereas the complete gene sets are provided as Additional file 2 (AB1700 data) and Additional file 3 (Illumina data). Fold differences ranged between 1.4 and 13.9. As can be seen from Figures 2, 3 and 4 and Additional file 2, gene expression levels vary within samples from the same cortical region. Such variation within a group is quite common in gene expression studies of animal tissue and is one of the reasons why SAM is preferred over traditional statistical methods such as Student t-test and ANOVA when analysing microarray data. The entire list of regionally enriched genes, including probe and gene identifiers for both microarray platforms and observed fold differences is provided in Additional file 4.

**Figure 5:**
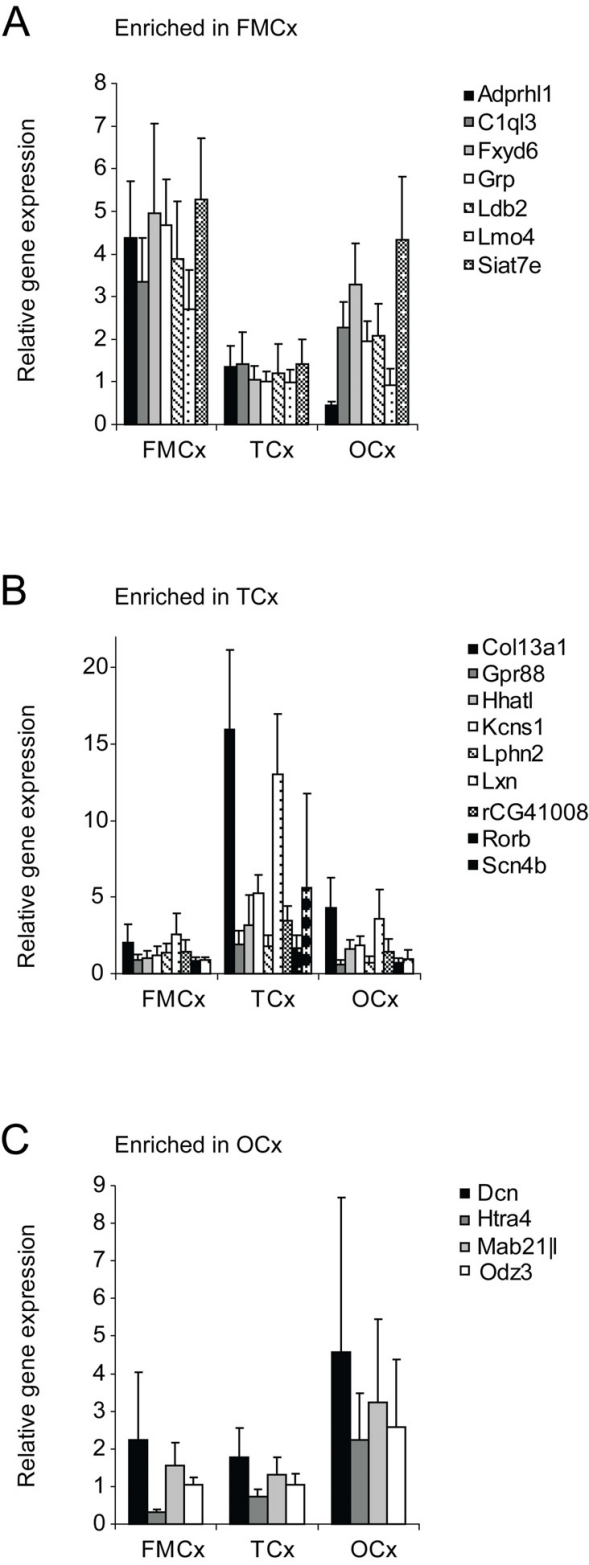
**Validation of regional enrichment by QPCR**. Relative gene expression as demonstrated by TaqMan QPCR for 20 selected genes enriched in rat A) FMCx, B) TCx and C) OCx. The diagrams display mean values for up to twelve replicates from each region, including left and right hemispheres from the three rats included in the microarray study as well as three additional rats included for validation purposes. Gene expression levels in the samples from the three additional rats completely correlated with data from the original rats. ANOVA (p < 0.05) confirmed significant regional enrichment across cortical regions for all genes, with p-values ranging from 10^-5 ^- 10^-11 ^for the FMCx genes, 10^-3 ^- 10^-12 ^for the TCx genes and 10^-2 ^- 10^-6 ^for the OCx genes. Expression of Crim1 and Atoh7 could not be detected by their respective TaqMan assays. Details on TaqMan assays and statistical results are included in Additional file 4. FMCx, fronto-medial cortex; TCx, temporal cortex; OCx, occipital cortex.

Among the 65 regionally enriched genes, we selected a subset of 22 genes for more detailed analysis (indicated by bold letters in Figure 1), including an independent validation by quantitative real-time PCR. Since most of our 65 regionally enriched genes were validated by independent analysis on a second microarray platform, we considered it unnecessary to validate all genes by QPCR. We used all cortical samples from the three original rats included in the microarray experiment, together with corresponding samples from three additional rats. As mentioned above for the microarray analyses, corresponding left and right samples from the same cortical region were treated as individual replicates. The QPCR analyses confirmed the regional enrichment of all but two of these 22 genes (*Atoh7 *and *Crim1*), whose transcripts could not be amplified in any of the cortical samples (Figure 5). Gene expression levels in the samples from the three additional rats completely correlated with data from the original rats. Details on TaqMan assays and statistical results are included in Additional file 4.

### General features of genes enriched in rat frontomedial-, temporal- or occipital cortex

Genes preferentially expressed in one of the three cortical areas also differed in their expression patterns in other regions of the brain (Figures 2, 3 and 4 and Additional file 2). In general, TCx enriched genes showed the strongest region-specific patterns compared to those of FMCx and OCx, which displayed a higher degree of co-expression in the other brain regions examined. Nine of the 24 TCx genes showed none, or only weak, expression in other brain regions and tissues, such as *Atoh7, Col13a1 and Kcns1*. The remaining TCx genes were co-enriched either in striatum, cerebellum or in hippocampus. In comparison, only four of the 30 FMCx genes showed specific enrichment in the FMCx (i.e. *Ephb6*, *Lmo4*, *Nags *and *Panx1*), but none of them were exclusively detected there. Interestingly, thirteen of the FMCx genes were co-enriched in the hippocampus (e.g. *C1ql3*, *Crim1 *and *Siat7e*). In comparison, only one TCx gene (*Nef3*) and one OCx gene (*Htr5b*) had a similar co-enrichment in the hippocampus.

### Functional annotation of the regionally enriched cortex genes

**Figure 6:**
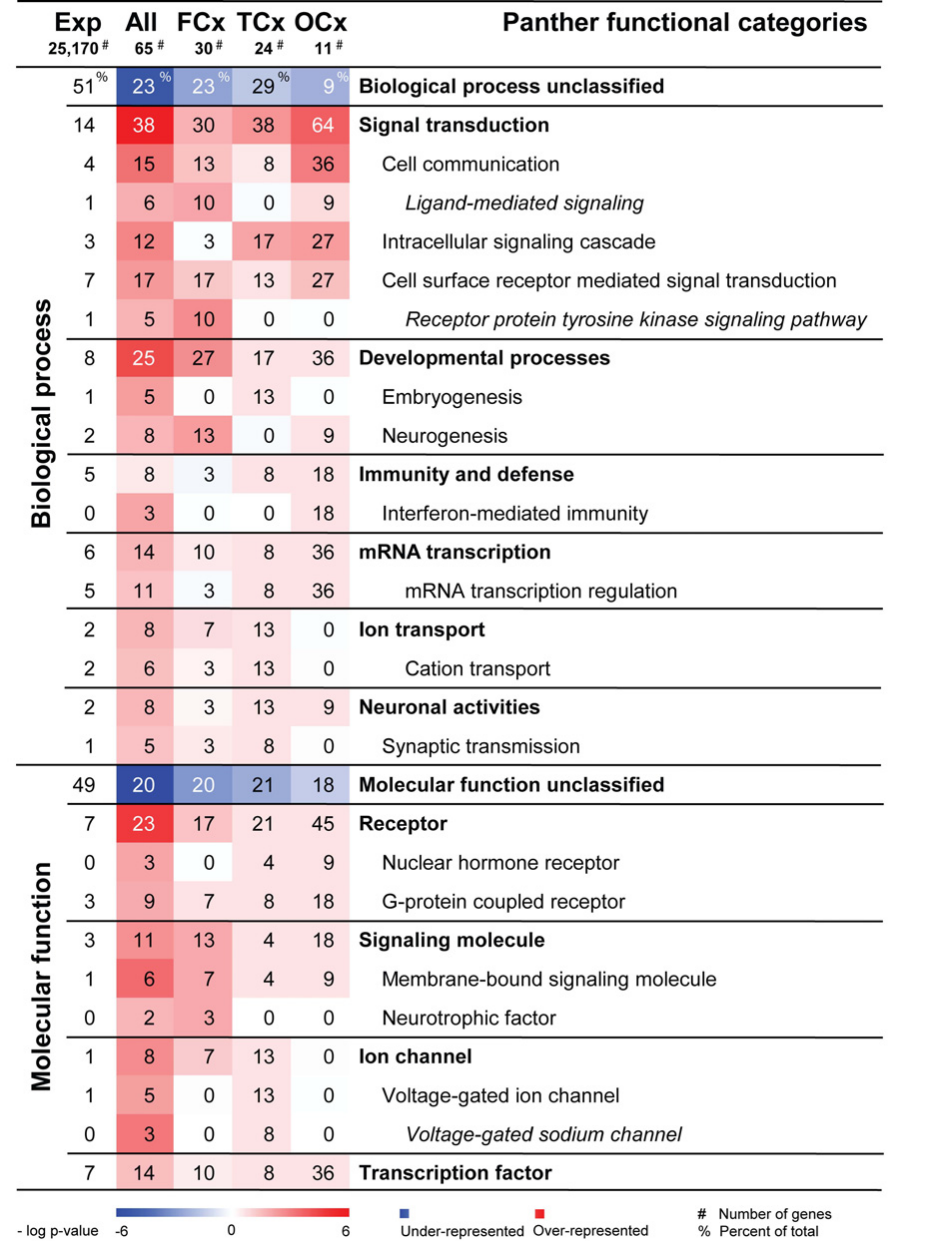
**Functional characterisation of the 65 genes regionally enriched in cortex subregions**. Panther was used to search for over-represented biological processes and molecular functions among the sub-regionally enriched cortex-genes. The heat map demonstrates the significance of over- and under-represented Panther categories in the entire set of 65 cortical genes (column 2), the 30 FMCx genes (column 3), the 24 TCx genes (column 4) and the 11 OCx genes (column 5). The colour intensity displays the statistical significance (negative log p-value) of over- and under-represented functional categories. Red colour signifies an over-representation of genes mapping to a certain term, blue colour an under-representation and white a representation as expected, based on the overall distribution on the array. Numbers presented on the heat map indicate the percentage of genes within a gene set that map to the given category, e.g. 38% of the 65 regional genes map to the biological process 'signal transduction'. The first column states the overall distribution of a term among the 25,170 genes with detectable expression in the data set, e.g. 13.6% of the 65 regional genes were expected to map to 'signal transduction', hence this category is significantly over-represented among our regional genes. Exp, expected (based on overall distribution on array); Cx, cortex; FCx, fronto-medial cortex; TCx, temporal cortex; OCx, occipital cortex; #, number of genes in each gene set; %, percentage of genes.

In the same rat model, we have previously shown that functional annotations of genes preferably expressed in a certain brain region reflect the functional specialisation of the given area [20]. We therefore mapped the regionally enriched cortex genes, both the entire set of 65 cortical genes and each regional set individually, to the Panther annotation categories to search for significant over-representations of particular functional groups compared to the overall distribution of the 25,170 genes detected on the AB1700 Rat Genome Survey array (Figure 6). At first glance it is interesting to note that only 23% of the 65 genes examined were so far un-annotated, with no known function. This is far less than expected based on the overall distribution of un-annotated genes on the microarray (~50%) (Figure 6).

Strikingly, 38% of the 65 genes were found to be involved in signal transduction; primarily cell communication, cell surface receptor mediated signal transduction and intracellular signalling cascades. This represents a massive over-representation (p < 10^-6^) compared to the overall distribution of such genes present on the microarray. This observation is also valid for each of the three cortical gene sets individually, where additional subtle differences may be observed, despite the low number of genes in each regional set (Figure 6). Signal transduction genes range from 30% among the FCx genes to a massive 64% among the OCx-genes. There is also a differentiation between subclasses of signal transduction. Cell communication and intracellular signalling cascades seems most prominent among the OCx genes, whereas genes in the receptor protein tyrosine kinase pathway are only found among the FCx genes. Similar differences between cortical regions can also be spotted among other functional categories. Genes mapping to the embryogenesis are only found among the TCx-genes and genes involved in neurogenesis are only found among the FCx- and OCx-genes. Furthermore, ion channels are only found to be enriched in the TCx and immunity-related genes mainly among the OCx-genes.

### Spatial and time-dependent expression patterns among the regionally enriched genes

The 65 genes enriched in either FMCx, TCx or OCx were examined with respect to their expression in other aspects of the cerebral cortex, such as during cortical development and in different layers and cell types. We imported raw microarray data from relevant, publicly available studies in rodents (Table 1) and re-analysed them with focus on the 65 regionally enriched genes. For illustration purposes, Figures 2, 3 and 4 present the resulting expression profiles for the 22 genes that were validated by QPCR, whereas profiles for the remaining 43 genes are available as additional material; see below for details.

#### Expression patterns in the developing cerebral cortex

Since proper arealisation of the neocortex may be governed by differential expression of regional genes at several stages of development, we aimed at exploring to what degree the genes in question were active during cortical development. Stead *et al. *have used transcriptional profiling to examine expression levels of 9,955 genes in rat frontal cortex, hippocampus and hypothalamus across up to four stages of prenatal development and seven stages of postnatal development [10]. We were able to map 32 of the 65 regionally enriched genes to probes represented in the Stead *et al. *data set, of which 29 showed significant differential expression among developmental stages (ANOVA, p < 0.05, Additional file 5). Interestingly, about 70% of the mapped genes show a peak or an increase in expression level in frontal cortex around or just after birth (e.g. *Fxyd6 *and *Grp*; Figure 2 and Additional files 5 and 6). For comparison, only about 30% of the examined housekeeping genes (N = 39) show similar trends (Additional files 5 and 6).

#### Laminar and cellular expression patterns in adult cerebral cortex

The structure of the neocortex is highly complex, with numerous neuronal subtypes that are intermingled with various glial cell types and other non-neuronal cells, as well as its six cellular layers that are distinguished by their morphology and the connections that they make. Because gene expression profiles obtained from entire cortical sub-regions lack spatial resolution, we wanted to examine whether the regional enrichment of our set of 65 genes was a shared feature among many cell types, or comprised by certain layers or cells. It is important to note that laminar and cellular information was only available for the mouse brain and not for the rat, although one can expect a high overlap in preferences between these two species.

In order to study laminar expression patterns of the set of cortex genes, we examined the Allen Mouse Brain Atlas, which is a comprehensive resource presenting *in situ *hybridisation images of coronal and sagittal sections of about 20,000 genes in the mouse brain [32]. We found that 56 of our 65 cortical genes were represented and that 35 of these showed laminar-restricted patterns of expression (Figures 2, 3 and 4 and Additional files 5 and 7).

To study cellular preferences of the 65 genes, we also re-analysed a data set comparing gene expression in differentiating as well as mature astrocytes, oligodendrocytes and neurons [28]. Forty-two genes were represented in this data set and, interestingly, 18 of these were markedly enriched in neurons rather than in astrocytes and oligodendrocytes (e.g. *Grp *and *Gpr88*; Figures 2 and 3 and Additional files 5 and 8). In comparison, only one of the 42 represented genes showed significant enrichment in oligodendrocytes (i.e. *Sulf2*), whereas seven were enriched in astrocytes (e.g. *Lxn *and *Rorb*; Figure 3).

There are many different subtypes of neurons and Sugino *et al. *examined gene expression among 12 different populations of neurons in the adult mouse forebrain [30]. We explored the seven neocortical populations represented there; three GABAergic and two glutamatergic from the cingulate cortex, together with one GABAergic and one glutamatergic population from the somatosensory cortex. Fifty-one of the 65 regionally enriched genes were represented in this data set, of which 38 showed significant differential expression across the seven neocortical neuronal populations (e.g., *C1ql3*, *Lmo4 *and *Odz3*; Figures 2 and 4 and Additional files 5 and 9). Seventeen of the represented genes were enriched in glutamatergic populations, and interestingly, eight of these were among the FMCx-enriched genes (e.g., *C1ql3*, and *Lmo4*). In comparison, only one of the FMCx genes was enriched in GABAergic populations (i.e. *Hap1*). No such tendencies were observed for the TCx- and OCx genes.

## Discussion

### General characteristics of genes enriched in specific cortical regions

Recent microarray studies on mammalian brain have demonstrated very high similarities in global gene expression across different areas of the adult cortex, and in human and primate brain, the inter-individual transcriptome variation in samples from the same area tends to be much larger than variation between different cortical regions in one individual [17-20]. Still, various areas of the cortex have quite distinct functions, suggesting that certain genes are differentially expressed in each area to support its specific tasks, although few such factors have been identified so far.

We here present 65 genes that display enriched expression in the frontomedial- (FMCx), temporal- (TCx) or occipital (OCx) cortex of the adult rat brain. These highly selected genes were identified by the use of two independent microarray platforms, applying a strict statistical approach, combined with manual re-inspection of the resulting gene expression profiles and in part validation by QPCR. In support of our findings, several of the identified regionally enriched genes have previously been described to display patterned expression in developing or adult cortex, e.g. *Lxn, Rorb, Nr2f1, Lmo4 *and *Odz3 *(see below) [8,14,33-35].

It is important to note that the concept of cortical subregion-enriched expression does not equal cortex-specific expression; the genes identified in this study may show co-expression or even co-enrichment in other regions of the brain outside the cerebral cortex and other tissues. Thus, the genes enriched in the different cortical areas also differ in their expression patterns in other regions of the brain, illustrated here by the co-enrichment of several genes in FMCx and OCx as well as in FMCx and hippocampus. In line with this situation, ten of the regionally enriched cortical genes identified by us (e.g. *Ldb2*, *Gpr88 *and *Mab21|l*) were also reported to show differential expression in human and mouse motor cortex, caudate nucleus and cerebellum [36]. Furthermore, *Gpr88*, a rat TCx gene showing strong co-expression in striatum (Figure 3), was identified as a human caudate- and mouse striatum-specific gene by Strand *et al. *[36].

The largest contrasts in gene expression levels were found when comparing FMCx and TCx. It is possible that the genes co-enriched in FMCx and OCx in reality could represent medially enriched genes, perhaps with a high medial - low lateral gradient-like distribution. If this is the case, then it is equally possible that TCx-enriched genes are laterally enriched rather than being specific for the TCx. *In situ *images from the Allen Brain Atlas suggest this situation for at least *Lxn *and *Rorb*. Gradient-like distribution of gene expression in the adult cortex has previously been discussed as an alternative to region-specific expression [37].

### Functional roles of sub-regionally expressed genes in the cortex

The majority of the 65 sub-regionally enriched genes were involved in signal transduction processes, including cell communication, cell surface receptor mediated signal transduction and intracellular signalling cascades, but also in developmental processes, such as embryogenesis and neurogenesis (see Figure 6). This highly significant over-representation of certain biological functions suggests that the selected genes have important roles in maintaining specialised neuronal functioning in the cortical areas, supported by the fact that strikingly many of the 65 genes already have been classified and annotated. The importance of cell signalling genes in cortex has previously been reported when comparing human and primate cortical areas [18], and genes coding for proteins involved in signalling processes also constitute a large percentage of genes differentially expressed between other brain regions [20,38].

In line with this, a majority of the genes were markedly enriched in neurons rather than in astrocytes and oligodendrocytes, and about two-thirds displayed laminar expression by analysing *in situ *images in the Allen Mouse Brain Atlas, as was observed for e.g. *Fxyd6 *and *Lmo4 *(see Figure 2). Our finding suggests that the genes may have specific functions at certain layers of the relevant cortical areas, e.g. by being expressed in only one or a few types of neurons.

This notion is further supported by our re-analysis of data from Sugino and co-workers, who have examined gene expression among 12 different populations of neurons in the adult mouse forebrain [30]. A high proportion of the 65 genes were represented in their data set, and interestingly, about three-fourths of these showed significant differential expression across seven neocortical neuronal populations (e.g., *C1ql3*, *Lmo4 *and *Odz3*). Many of the represented genes were enriched in glutamatergic populations, of which eight were among the FMCx-enriched genes (e.g., *C1ql3 *and *Lmo4*). The functional specificity of an area may be conferred by certain cells or layers within that region. It is therefore plausible that many of our genes may be involved in the execution of region-specific tasks.

It is also worth noticing that according to re-analysis of data from Stead *et al. *[10], more than two-thirds of the regionally enriched genes examined show a peak or an increase in expression level in rat frontal cortex around or just after birth (e.g. *Fxyd6 *and *Grp*), as compared to less than one-third of a representative selection of house-keeping genes.

We have further mapped the 65 genes to studies comparing gene expression in different regions and at different stages of the developing mouse cerebral cortex [5,6,12-14], but we were unable to detect much overlap in regional enrichment (not shown). It has been indicated that "cortical" regions with unclear borders and/or immature laminar structures may not be converted to area-specific cytoarchitectures with sharp limits until the postnatal stages of development [5], thus regional enrichment of our 65 genes might not yet have been established at these stages.

### Regionally enriched cortex genes and brain function: Selected examples

Many of the 65 genes presented here have previously been shown as involved in, or required for, normal development and function of the brain. Their preferential expression in certain parts of the cortex could be imprints of gradient like transcription patterns or related to execution of locus-specific tasks. Transgenic or mutant mouse lines have been generated for several of the regionally enriched cortex genes. Many of the knock-out mutants are lethal or have severe phenotypes in multiple tissues, especially within the brain and CNS (see Additional file 10).

*Nr2f1*, a well known transcription factor playing a crucial role in patterning of the neocortex, is highly expressed in the caudate part of the murine neocortex during development [8,39], corresponding well with our finding of regional enrichment in the OCx in the rat. Cortical knock-out of the murine *Nr2f1 *was shown to induce massive expansion of frontal areas, including the motor cortex. These areas then occupied most of the neocortex, paralleled by a marked compression of the sensory areas to caudal OCx. These findings demonstrate that *Nr2f1 *is required for balancing the patterning of the neocortex into frontal/motor and sensory areas by repressing frontal/motor area identities and to specify sensory area identities [40]. Another gene thought to be involved in neocortical arealisation and displaying high caudal to low rostral graded expression, *Odz3 *[35], was also identified to be enriched in the OCx in our study.

The genes encoding the LIM-related proteins Lmo4 and Ldb2 were both found to be enriched in the FMCx. The LIM domain is an approximately 55-residue cysteine-rich zinc-binding motif mediating protein-protein interactions. Lmo4 has two LIM domains and may play a role as a transcriptional regulator, whereas Ldb2 has a LIM-binding domain that interacts with Lmo4 [41]. Both of them most likely function as enhancers to bring together diverse transcription factors [42]. The two interaction partners Lmo4 and Ldb2 seem to follow each other closely in most samples analysed in the present study (Figure 2). *Ldb2 *appears to be more prominent in OCx than *Lmo4*, but both genes show a strong preference for neurons rather than oligodendrocytes and astrocytes, and both are enriched in glutamatergic neurons of the cingulate cortex (Figure 2). Interestingly, in situ hybridisations indicate an enrichment of *Ldb2 *in deeper layer neurons, while *Lmo4 *appears more prominent in layers 2/3 (Figure 2). *LMO4 *has previously been shown to be differentially expressed between left and right perisylvian cortex at early stages of human cortical development [14]. This asymmetry disappears at later stages, and our data do not indicate asymmetrical expression in the adult rat cortex (Additional files 3 and 5). Lmo4-mutant mice die *in utero *[43], while in a cortical knock-out model, the boundaries of cortical functional areas were perturbed [44]. Expression of cortical regional markers was changed and the somatosensory barrel subfield was shrinked. Thus, *Lmo4 *is essential for cortical development in mice, correlating well with its dramatic increase in expression level at the day of birth (Figure 2).

The FMCx-enriched gene *Grp *(also known as bombesin) is apparently important for the normal function of both the human and the mouse brain, and it has been shown that blockade of bombesin-like peptide receptors impair inhibitory avoidance learning in mice [45]. In the developing rat frontal cortex, *Grp *expression peaks around postnatal day 7 (Figure 2). A similar increase is not observed in hippocampus or hypothalamus, suggesting that *Grp *might be important for late cortical development.

The TCx-enriched gene *Lxn *has been identified as a molecular marker for regional specification in the rat neocortex, and is produced in a subset of neurons in the lateral but not dorsal neocortex, more specifically in glutamatergic neurons in the infragranular layer [46]. It has been suggested that the area- and lamina-specific distribution of the *Lxn*-expressing subpopulation of glutamatergic neurons is a distinctive feature that may contribute to the functional specialisation of the lateral cortical areas [46]. This regional specification occurs very early in cortical development, prior to thalamocortical interactions and the completion of neurogenesis [33]. We found that expression levels of *Lxn *increase dramatically around postnatal day 1 in frontal cortex, hippocampus and hypothalamus (Figure 3), despite its reported regional specificity early in cortical development. *In situ *images from the Allen Brain Atlas confirm the regional specificity in deeper layer neurons of the lateral cortex (Figure 3). Knowing that *Lxn *has a highly restricted pattern of expression in cortical neurons, it is interesting to note that it seems to be highly enriched in astrocytes compared to neurons (Figure 3).

Despite enormous differences in size and complexity between the neocortex of rodents and humans, certain brain functions are organised in the same areas throughout the line of mammalian species [47,48]. This fact could imply that such functionally distinct areas have developed unique structures to cope with the need for different information processing and that expression of area specific genes is associated with the functional specialisation. When analysing a sub-set of the 65 regionally enriched genes in a pilot study of eight human cortical regions (dorso-lateral prefrontal cortex, primary motor cortex, primary sensory cortex, primary visual cortex, Broca's area, superior temporal gyrus, Heschl's gyrus and Wernicke's area) from three individuals, we were unable to observe the same regional enrichment as seen in adult rat (data not shown). This is in agreement with previous findings on the global level, where the variation across individuals appears far more pronounced than that observed across cortical regions in the same subject [18].

## Conclusions

To conclude, we have identified and validated the regional enrichment of 65 cortical genes within the FMCx, TCx or OCx of adult rat brain. We have further explored these genes and demonstrated specific patterns of expression in cortical development, -layers and -cell types. Such specific patterns of transcription are often linked to equally specialised functions and we therefore suggest that future in-depth characterization of the cortex sub-region enriched genes may shed new light on the functional divergence between cortical areas.

## Abbreviations

FMCx: fronto-medial cortex; TCx: temporal cortex; OCx: occipital cortex; SAM: significance analysis of microarrays; FDR: false discovery rate; QPCR: quantitative real-time PCR

## Authors' contributions

CS was responsible for the design of the study, the microarray experiments, the microarray data analysis, the QPCR validations, the analysis of external data and drafting the manuscript. KME was involved in the QPCR validations, the interpretation of the results and the drafting of the manuscript. PvdV was responsible for the human tissue dissections and involved in the interpretation of the results. VMS conceived of the study, was responsible for its design, participated in its coordination and data analysis and helped to draft the manuscript. All authors have read and approved the final manuscript.

## Supplementary Material

Additional file 1 **Numbers of regionally enriched genes**. This file presents the number of genes enriched in rat cortical regions according to the two microarray platforms and the different comparisons used in the study Click here for file

Additional file 2 **Gene expression profiles of regionally enriched genes on the AB1700 system**. This file displays the AB1700 gene expression profiles of all 65 regionally enriched genes in rat FMCx, TCx, OCx, hippocampus, striatum, cerebellum, liver, spleen and kidney. Individual samples are placed along the x-axis. The y-axis indicates quantile normalised signal intensities for each gene in each individual sample. Click here for file

Additional file 3 **Gene expression profiles of regionally enriched genes on the Illumina system**. This file displays the Illumina gene expression profiles of the 59 available probes of regionally enriched genes in rat FMCx, TCx and OCx. Individual samples are placed along the x-axis. The y-axis indicates quantile normalised signal intensities for each gene in each individual sample. Click here for file

Additional file 4 **Full list of regionally enriched genes identified in this study**. This file presents the full list of 65 genes enriched in FMCx, TCx or OCx, including gene ID, gene symbol, gene name, fold differences, AB1700-, Illumina- and TaqMan probe IDs and significance of differential expression according to each of the three platforms. Click here for file

Additional file 5 **Features of regionally enriched genes observed in other data sets**. This file lists expression features such as regional co-enrichment and preferences for certain cortical layers or cell types as well as tests for differential expression of regional genes in the different data sets used in the study. Each experiment has a separate data sheet; 1) regional co-enrichment in the rat brain [20], 2) developmental stages of the cortex [10], 3) cortical layers (Allen Mouse Brain Atlas), 4) cortical cell types [28] and 5) neuronal subtypes of the cortex [30]. Click here for file

Additional file 6 **Gene expression of regionally enriched genes throughout development of the rat cerebral cortex**. This file displays gene expression profiles of regionally enriched genes across pre- and postnatal stages of the developing rat cerebral cortex. Individual samples are placed along the x-axis; Cortex E16, E17, E18, E20, P01, P07, P14, P21, P30 and P90, Hippocampus P0, P07, P14, P21, P30 and P90, Hypothalamus E18, E20, P01, P07, P14, P21, P30 and P90; see original publication for details. The y-axis indicates quantile normalised signal intensities for each gene in each individual sample. Raw microarray data were obtained from [10]. 32 of our genes were represented in this data set. Click here for file

Additional file 7 **Laminar expression profiles of regionally enriched genes**. This file presents in situ hybridisation images demonstrating laminar expression patterns of the 56 regionally enriched genes that were represented in the Allen Mouse Brain Atlas. For FMCx and OCx genes, images presented in this study represent sagittal sections near midline (lateral ~0.7-1.4 mm). For TCx genes, coronal sections between Bregma -3.08 and -3.38 are presented where available. Only sagittal sections were available for *Arhgap9, Cabp1, Col13a1, Hhatl, Ikbke *and *Mox2r*, hence the lateral-most sections are presented for these genes. Click here for file

Additional file 8 **Expression of regionally enriched genes in astrocytes, oligodendrocytes and neurons**. This file shows gene expression profiles of regionally enriched genes across differentiating as well as mature astrocytes, oligodendrocytes and neurons. Individual samples are placed along the x-axis; Cultured Astroglia, Astrocytes P1, P7-P8 and P17, Astrocytes Gray P17, Oligodendrocyte progenitor cells, premyelinating, postmitotic oligodendrocytes (Myelin Oligos), Oligodendrocytes, Neurons P7n, P7, P16n and P16, see original publication for details. The y-axis indicates quantile normalised signal intensities for each gene in each individual sample. Raw microarray data were obtained from Cahoy et al [28]. 42 of our genes were represented in this data set. Click here for file

Additional file 9 **Expression of regionally enriched genes in different neuronal subtypes**. This file shows gene expression profiles of regionally enriched genes across different subtypes of neocortical neurons. Individual samples are placed along the x-axis; layer 6 glutamatergic neurons from cingulate cortex (CT6 strain), layer 5 glutamatergic neurons from cingulate cortex (YFPH strain), layer 5-6 glutamatergic neurons from somatosensory cortex (YFPH strain), layer 1-6 GABAergic neurons from cingulate cortex (G30 strain), layer 4-6 GABAergic neurons from cingulate cortex (G43 strain), layer 2-4 GABAergic neurons from cingulate cortex (GIN strain) and layer 1-6 GABAergic neurons from somatosensory cortex (G30 strain); see original publication for details. The y-axis indicates quantile normalised signal intensities for each gene in each individual sample. Raw microarray data were obtained from Sugino et al [30]. 51 of our genes were represented in this data set. Click here for file

Additional file 10 **Available knock-out mutants and transgenic lines**. This file lists available mouse knock-out mutants and transgenic lines and resulting phenotypes for the regionally enriched cortex genes. Click here for file
