# Supplementary material for: Gene expression in the rat brain: High similarity but unique differences between frontomedial-, temporal- and occipital cortex
Source: BMC Neurosci. 2011 Jan 26;12:15. doi: 10.1186/1471-2202-12-15 (PMC3040714; doi:10.1186/1471-2202-12-15)
Supplement: Additional file 2 — Gene expression profiles of regionally enriched genes on the AB1700 system. This file displays the AB1700 gene expression profiles of all 65 regionally enriched genes in rat FMCx, TCx, OCx, hippocampus, striatum, cerebellum, liver, spleen and kidney. Individual samples are placed along the x-axis. The y-axis indicates quantile normalised signal intensities for each gene in each individual sample. [file 1471-2202-12-15-S2.PPT]

## Slide 1
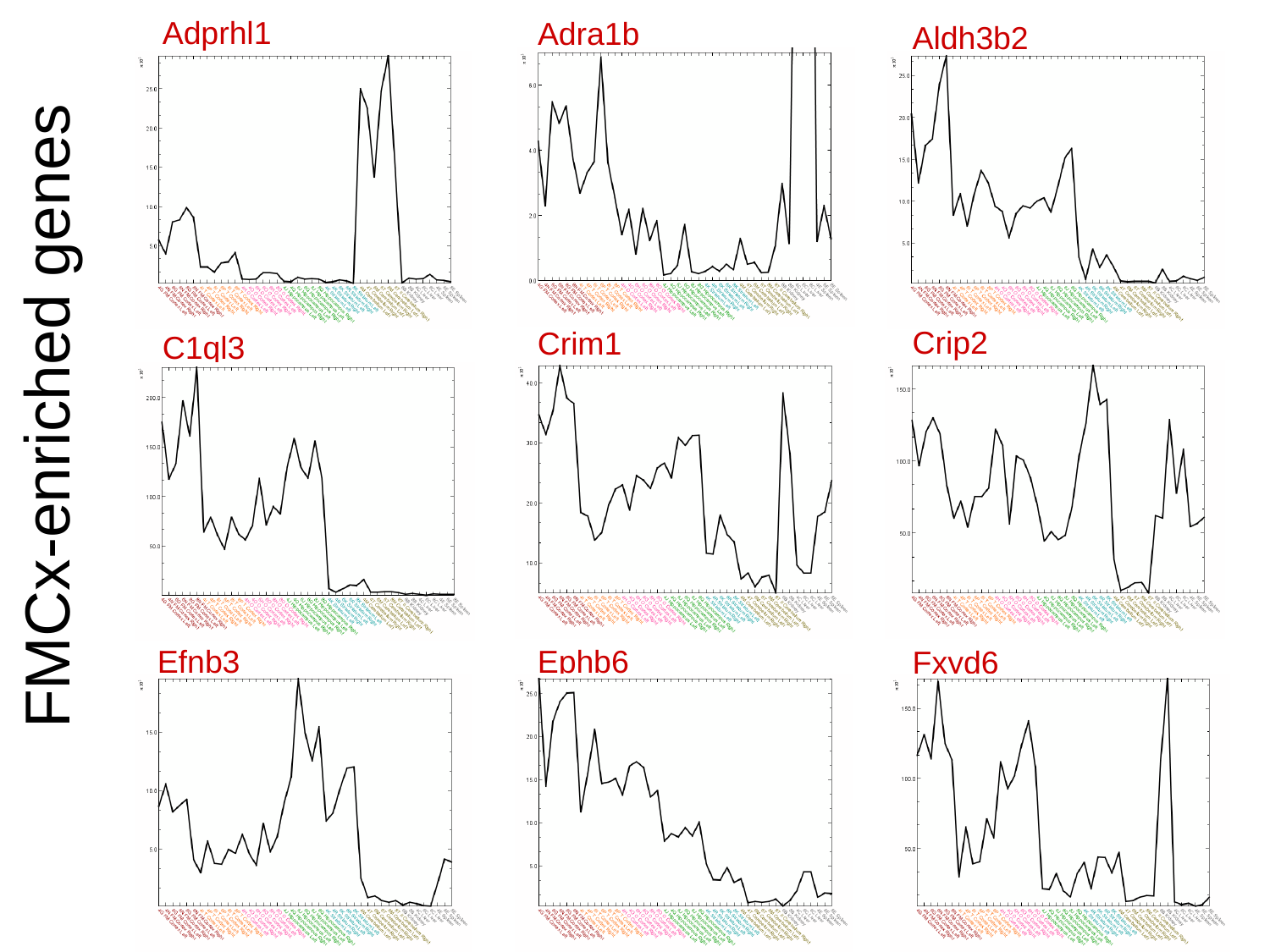

Adprhl1
Adra1b
Aldh3b2
Crip2
Crim1
C1ql3
FMCx-enriched genes
Efnb3
Ephb6
Fxyd6

## Slide 2
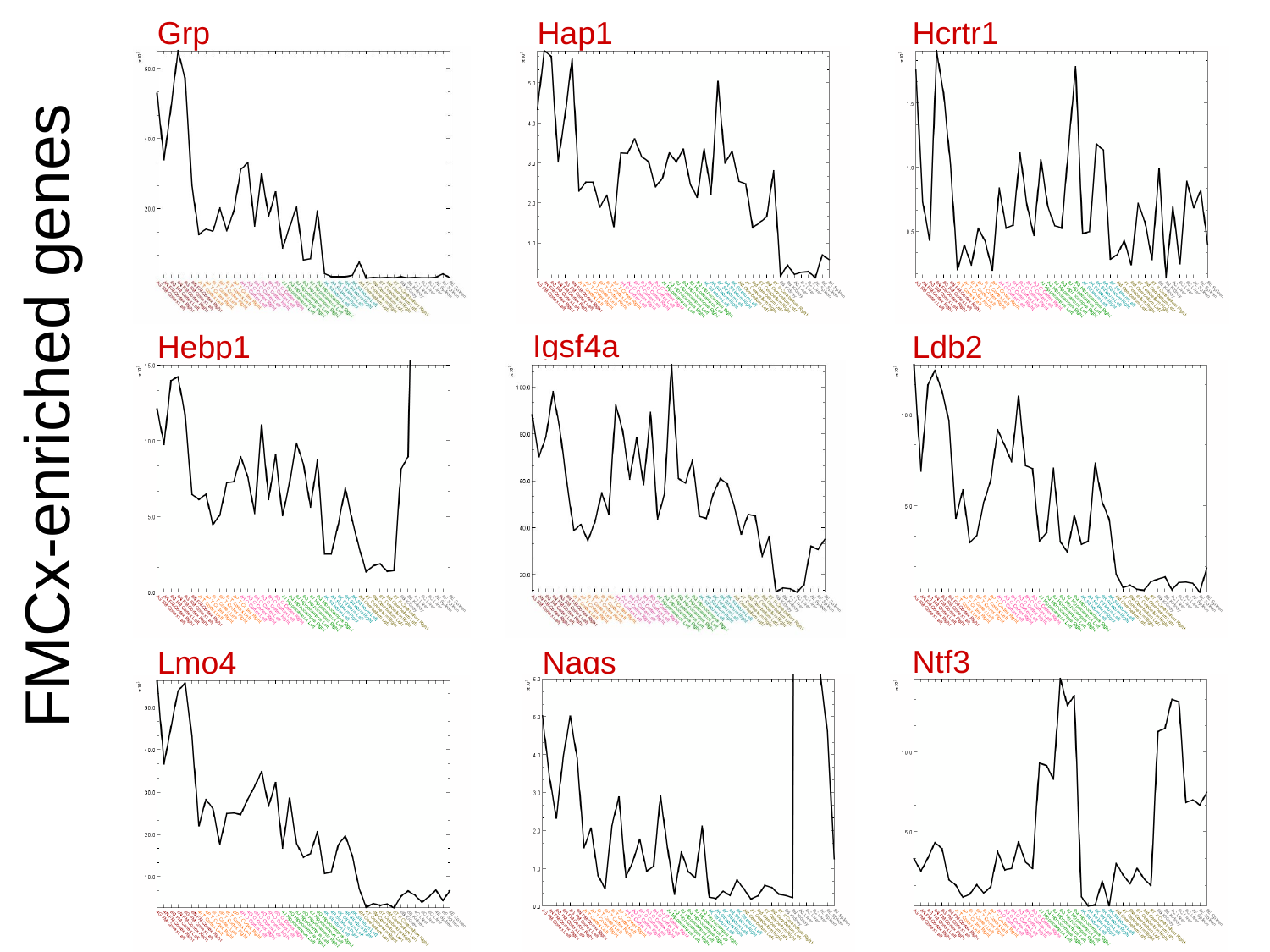

Grp
Hap1
Hcrtr1
Igsf4a
Hebp1
Ldb2
FMCx-enriched genes
Ntf3
Lmo4
Nags

## Slide 3
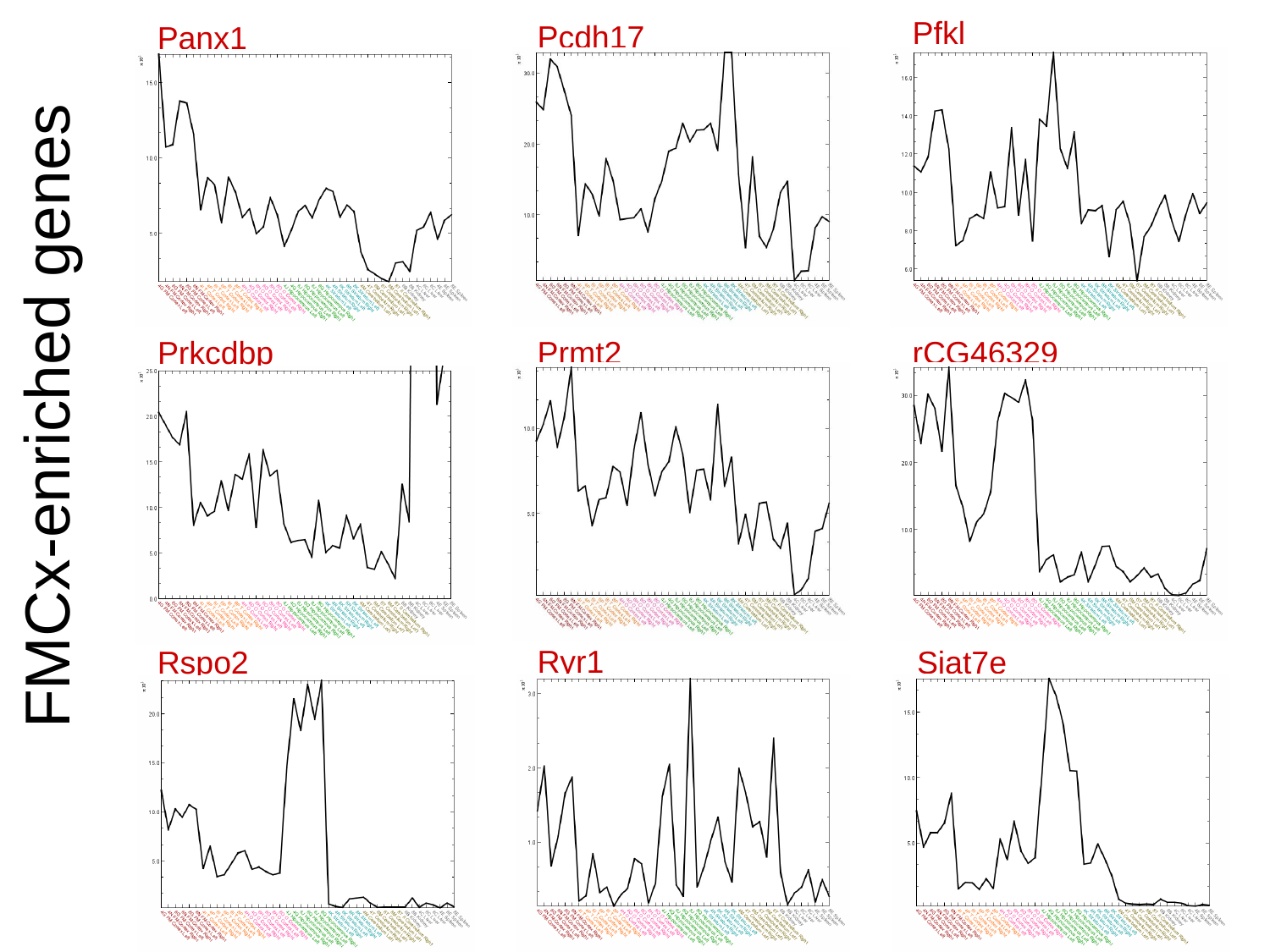

Pfkl
Pcdh17
Panx1
Prkcdbp
Prmt2
rCG46329
FMCx-enriched genes
Ryr1
Rspo2
Siat7e

## Slide 4
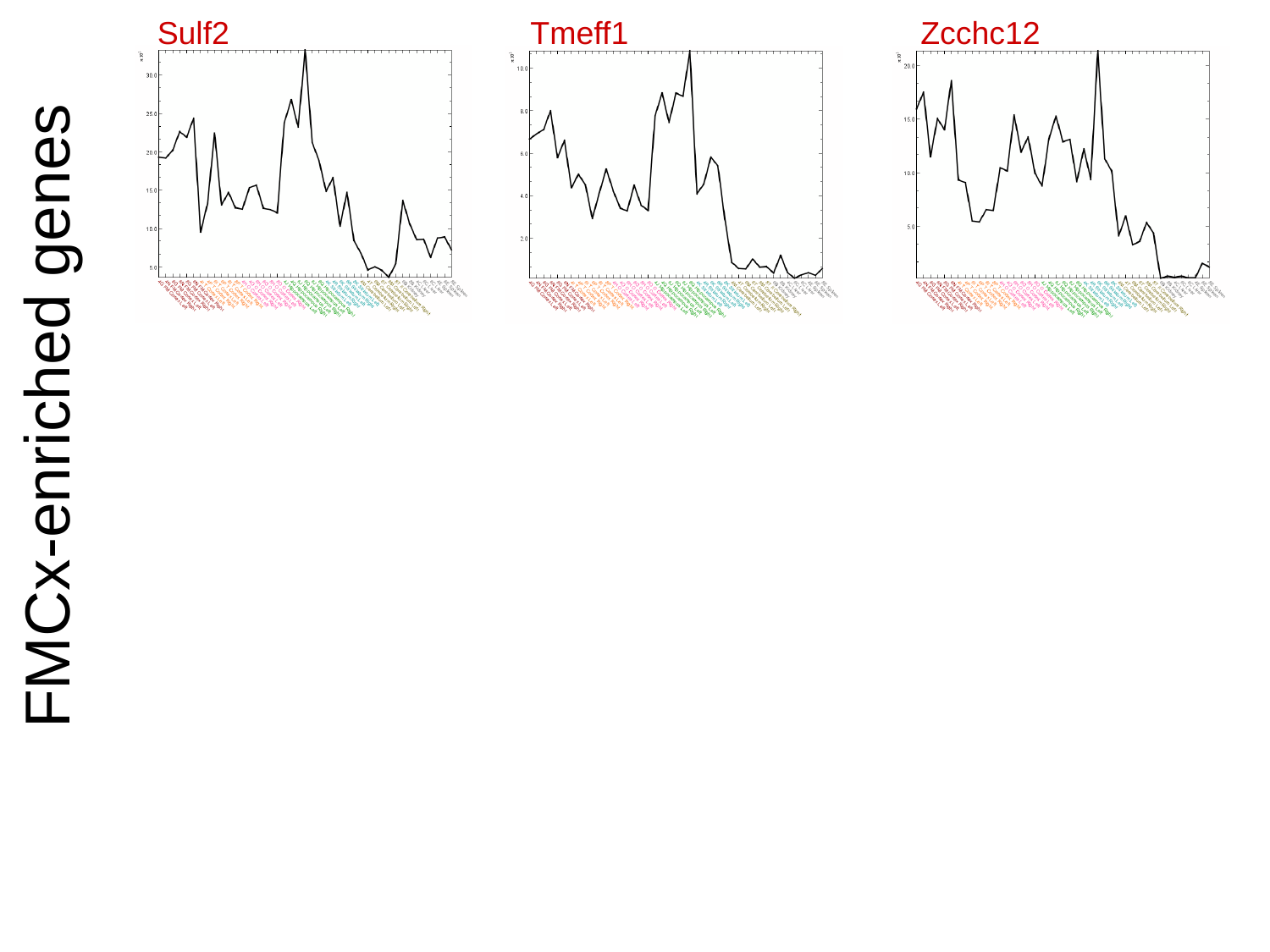

Sulf2
Tmeff1
Zcchc12
FMCx-enriched genes

## Slide 5
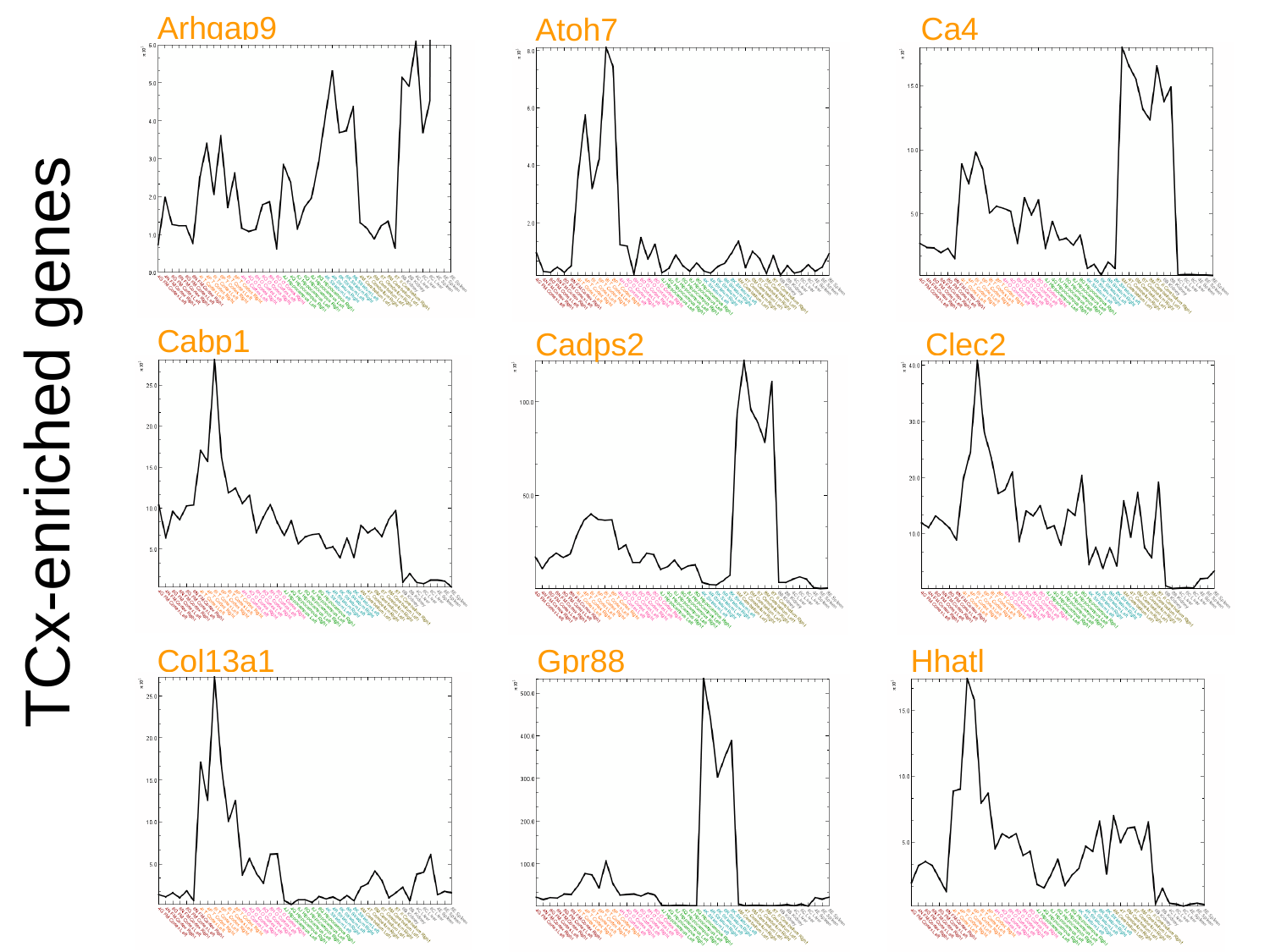

Arhgap9
Ca4
Atoh7
Cabp1
Cadps2
Clec2
TCx-enriched genes
Col13a1
Gpr88
Hhatl

## Slide 6
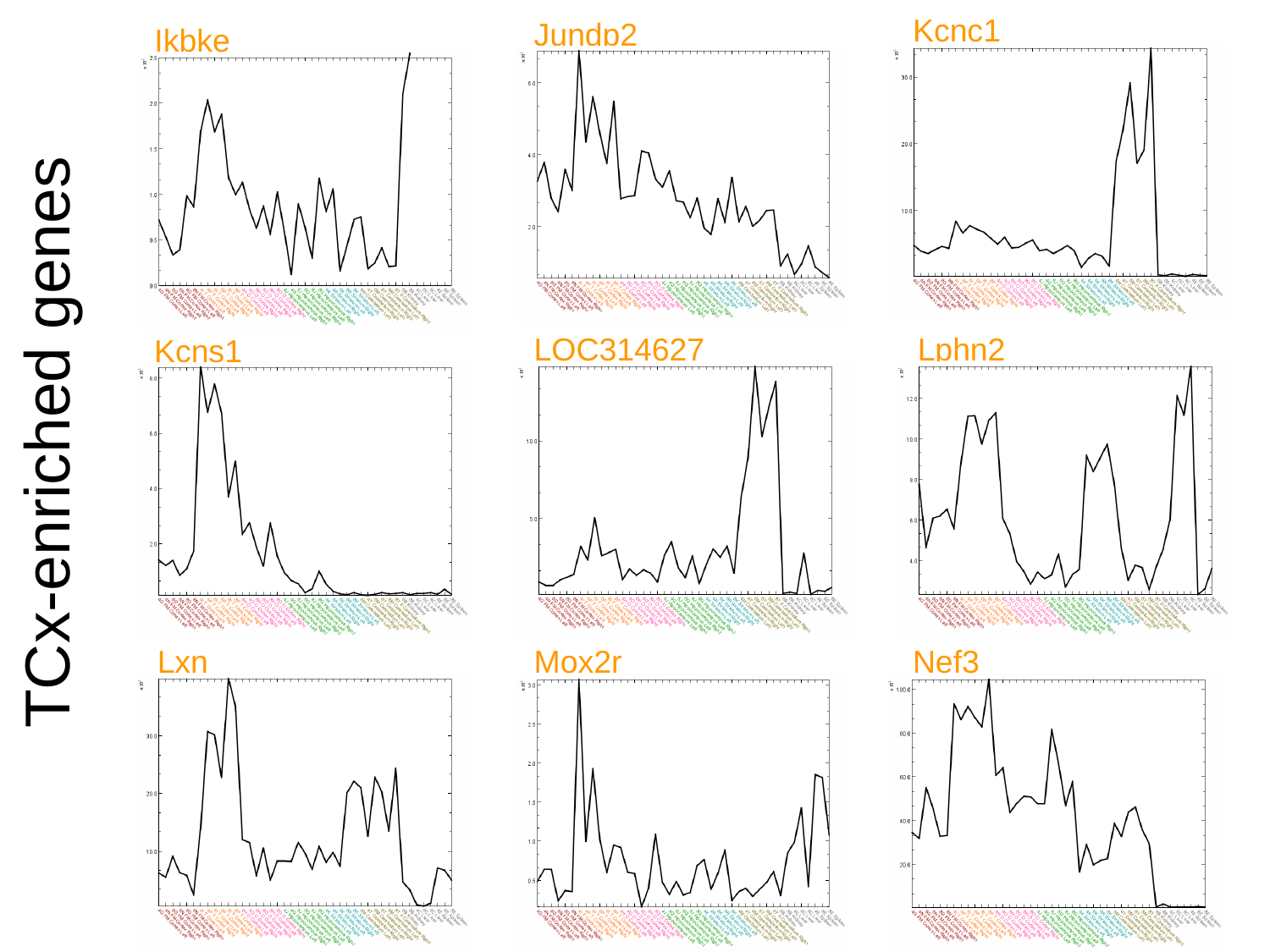

Kcnc1
Jundp2
Ikbke
LOC314627
Lphn2
Kcns1
TCx-enriched genes
Lxn
Mox2r
Nef3

## Slide 7
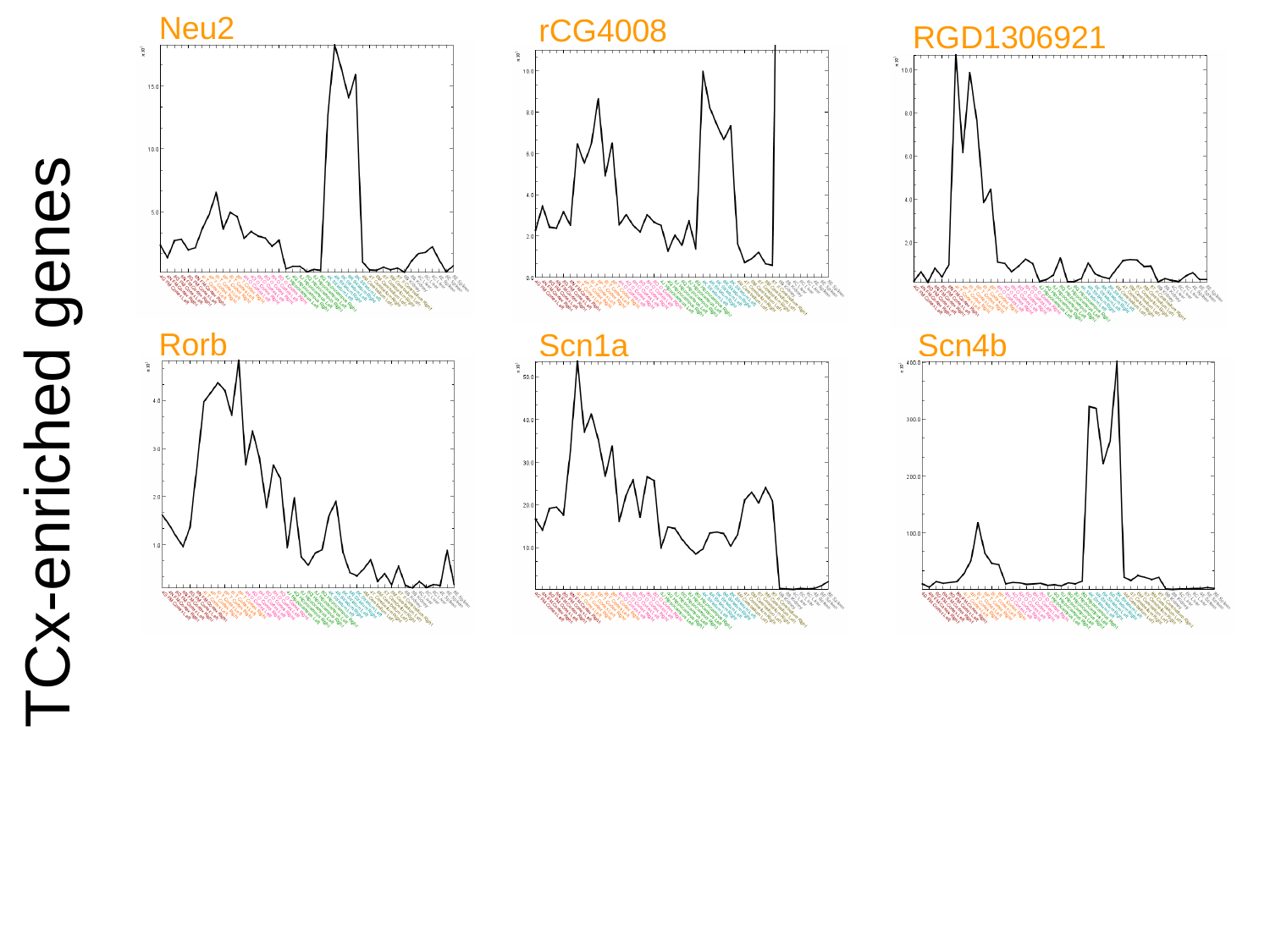

Neu2
rCG4008
RGD1306921
Rorb
Scn1a
Scn4b
TCx-enriched genes

## Slide 8
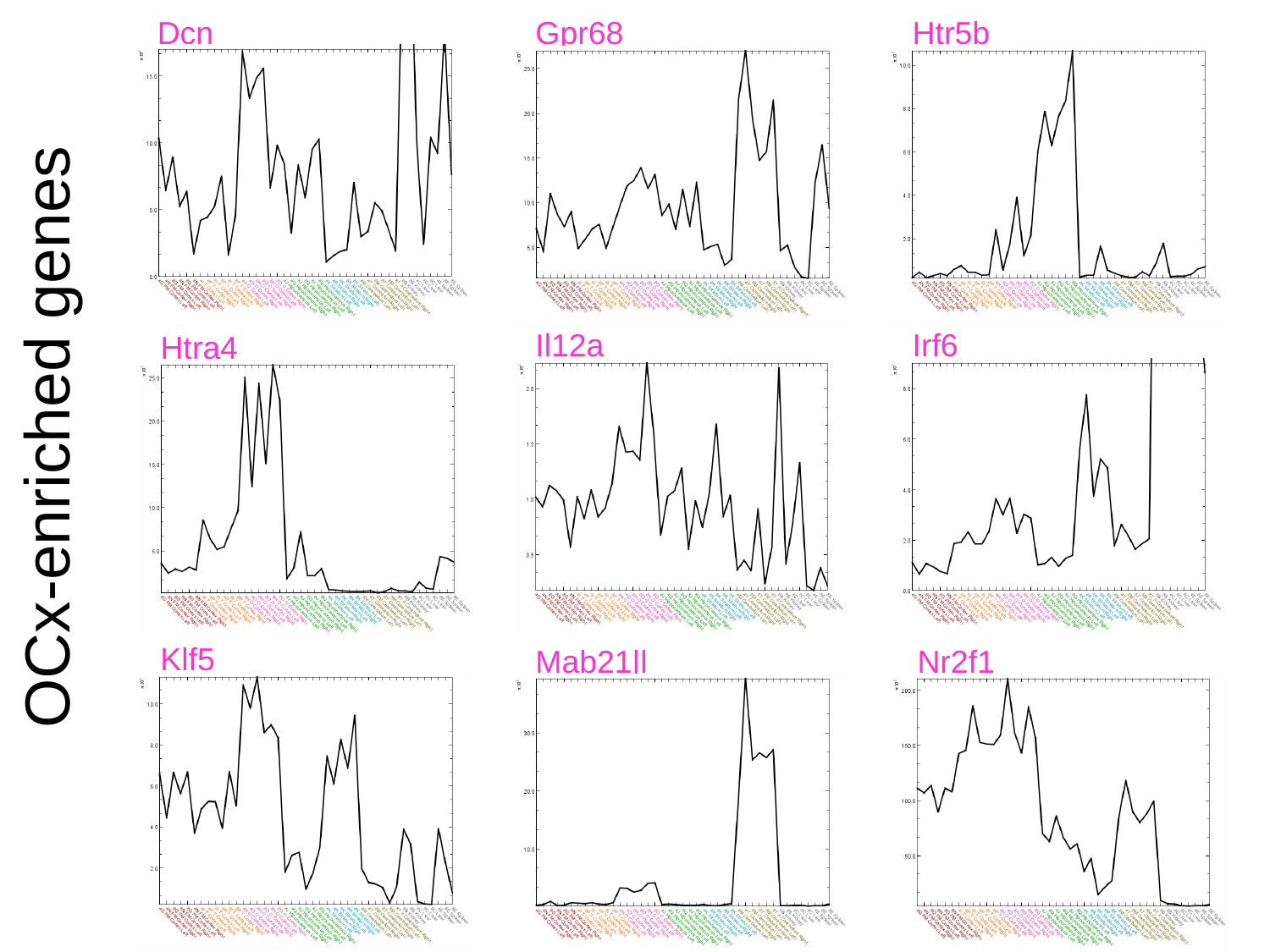

Dcn
Gpr68
Htr5b
Il12a
Irf6
Htra4
OCx-enriched genes
Klf5
Mab21|l
Nr2f1

## Slide 9
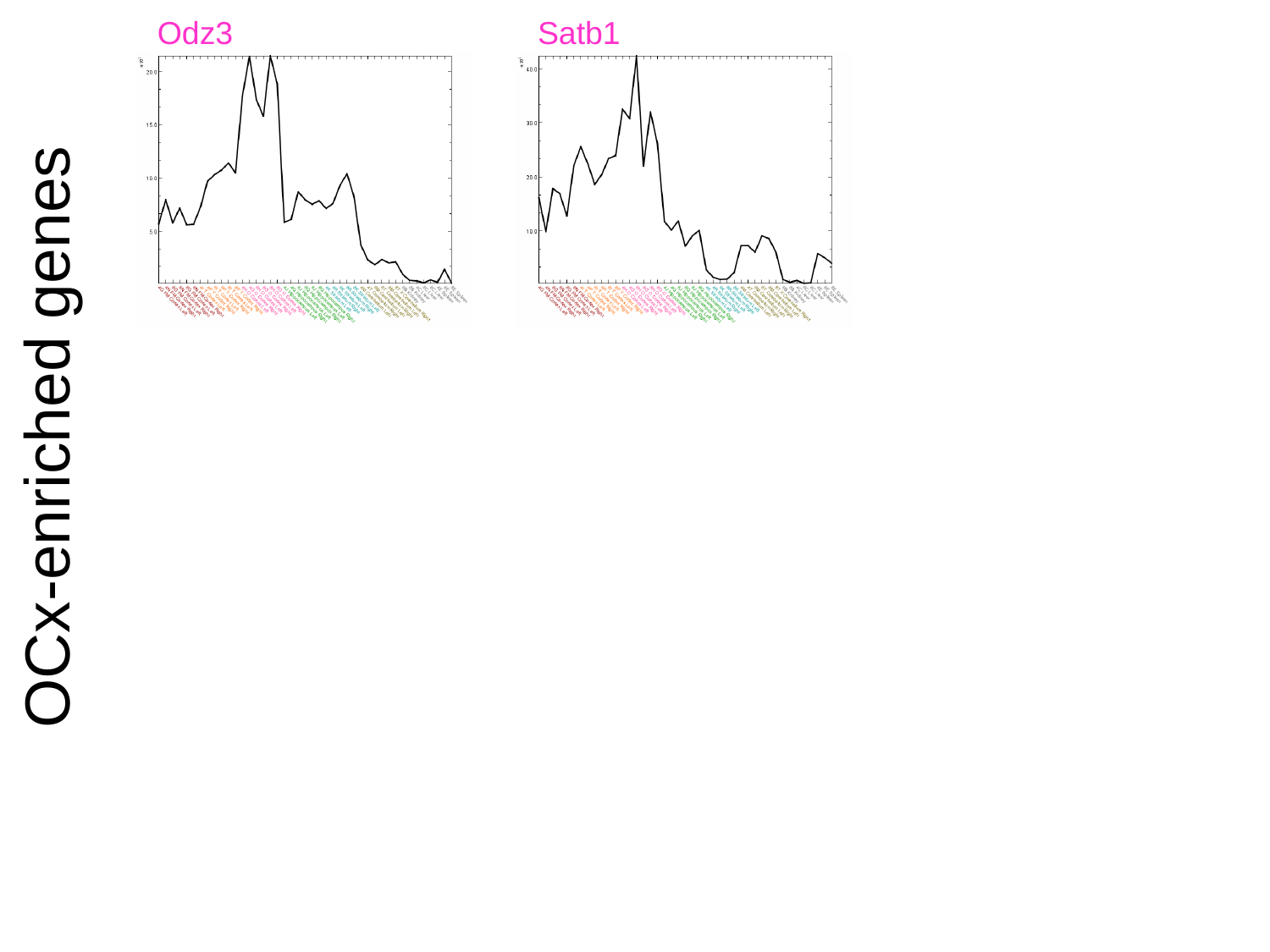

Odz3
Satb1
OCx-enriched genes
