# Supplementary material for: Gene expression in the rat brain: High similarity but unique differences between frontomedial-, temporal- and occipital cortex
Source: BMC Neurosci. 2011 Jan 26;12:15. doi: 10.1186/1471-2202-12-15 (PMC3040714; doi:10.1186/1471-2202-12-15)
Supplement: Additional file 3 — Gene expression profiles of regionally enriched genes on the Illumina system. This file displays the Illumina gene expression profiles of the 59 available probes of regionally enriched genes in rat FMCx, TCx and OCx. Individual samples are placed along the x-axis. The y-axis indicates quantile normalised signal intensities for each gene in each individual sample. [file 1471-2202-12-15-S3.PPT]

## Slide 1
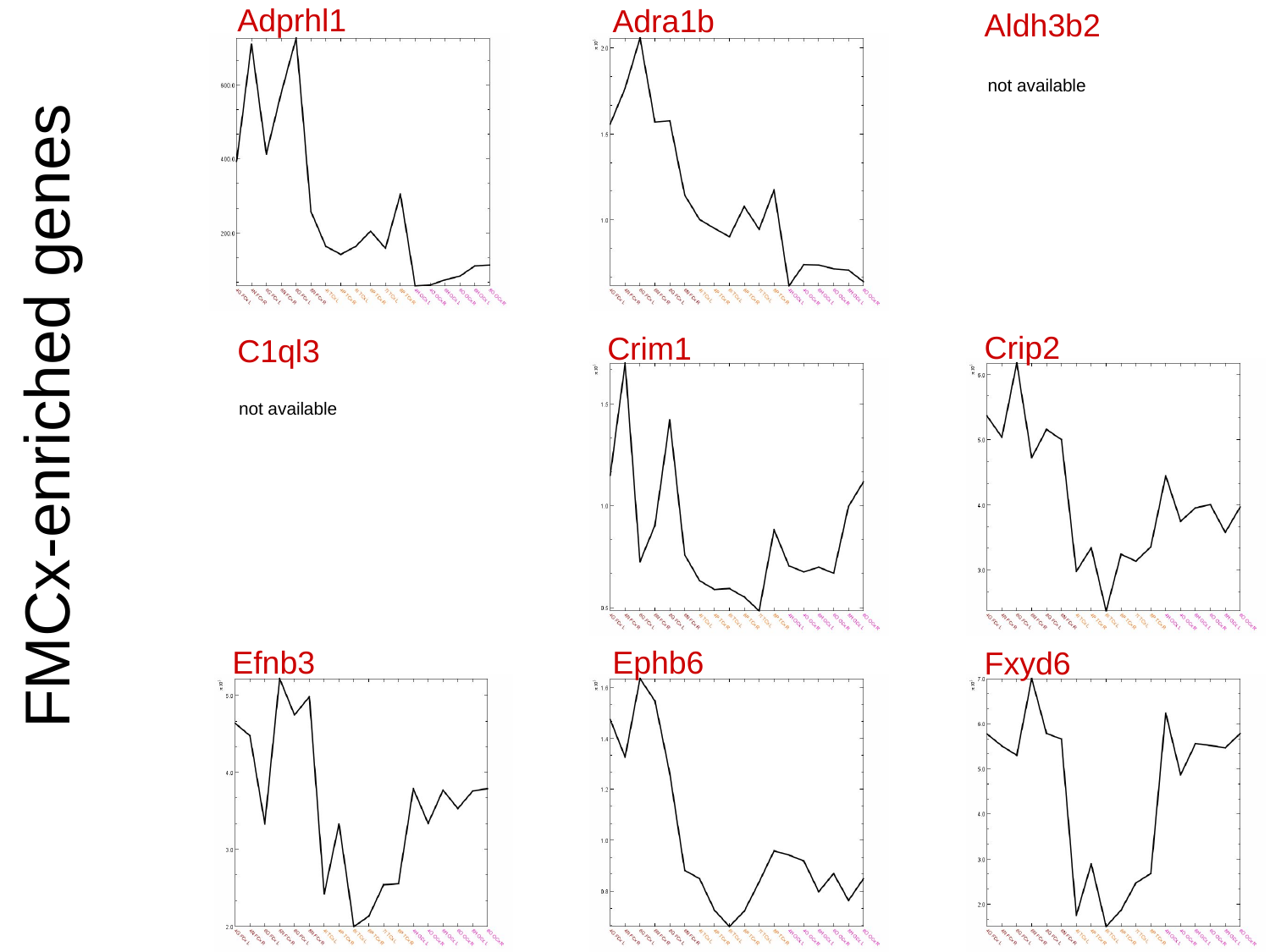

Adprhl1
Adra1b
Aldh3b2
not available
Crip2
Crim1
C1ql3
FMCx-enriched genes
not available
Efnb3
Ephb6
Fxyd6

## Slide 2
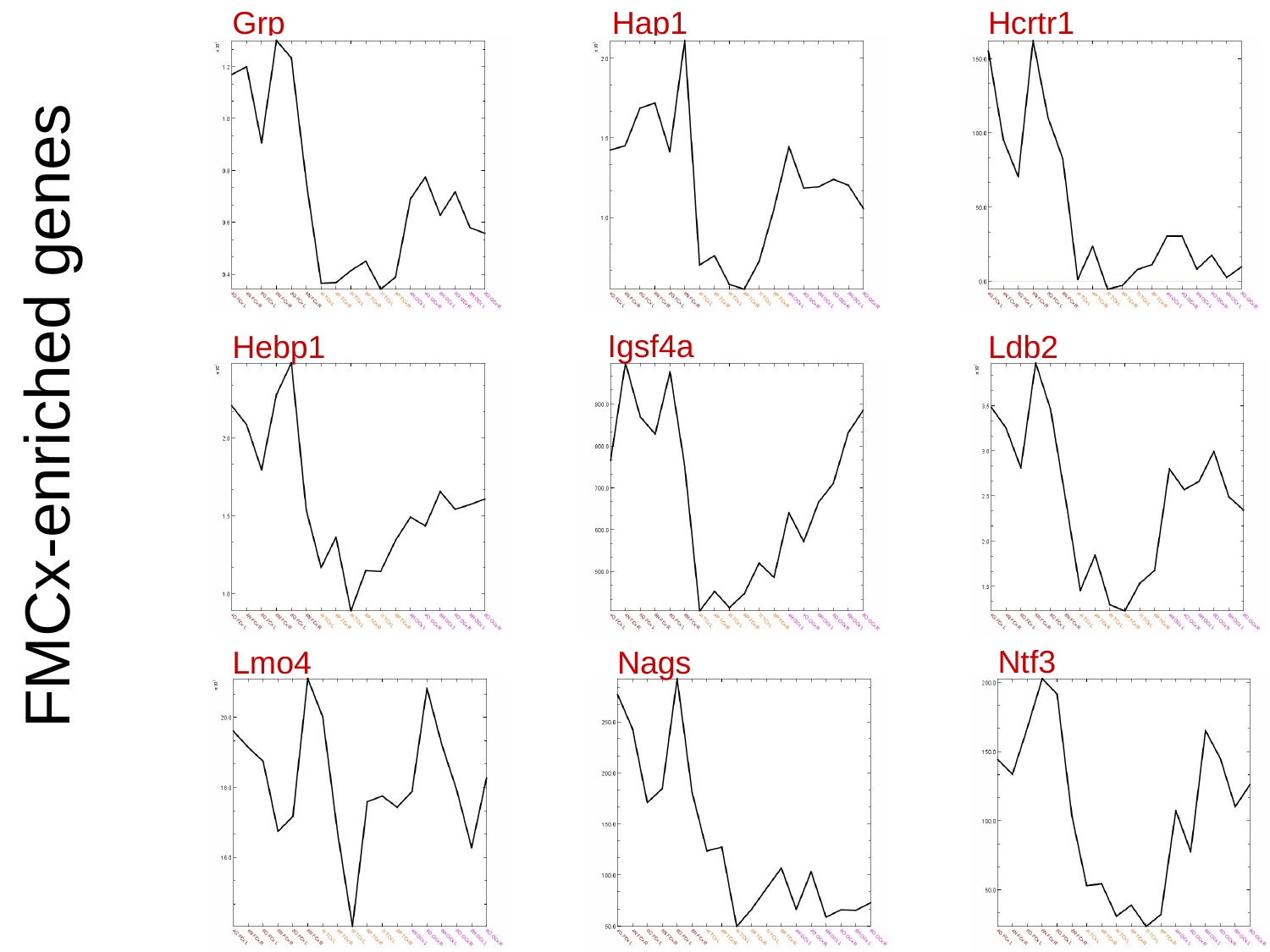

Grp
Hap1
Hcrtr1
Igsf4a
Hebp1
Ldb2
FMCx-enriched genes
Ntf3
Lmo4
Nags

## Slide 3
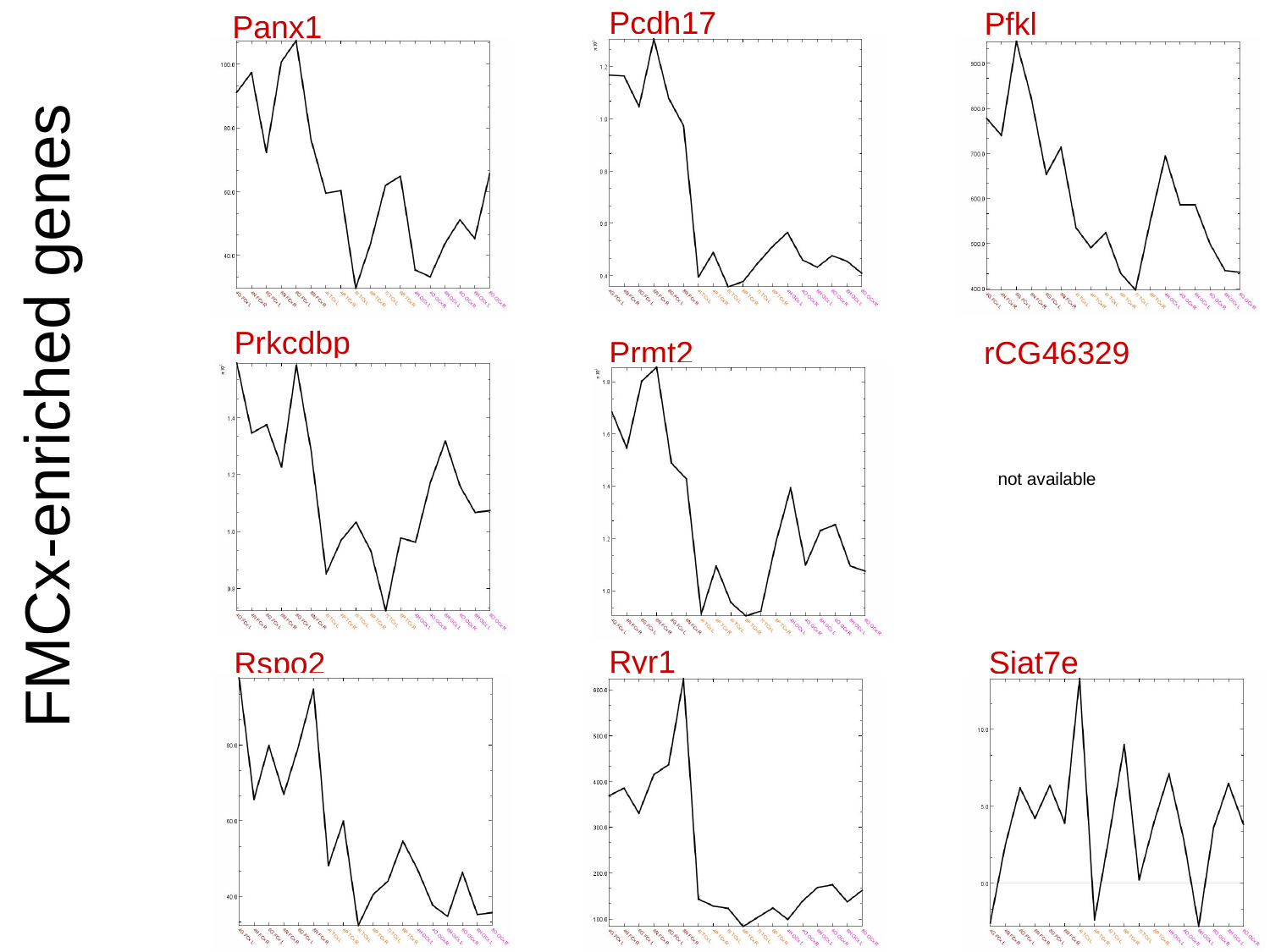

Pcdh17
Pfkl
Panx1
Prkcdbp
Prmt2
rCG46329
FMCx-enriched genes
not available
Ryr1
Siat7e
Rspo2

## Slide 4
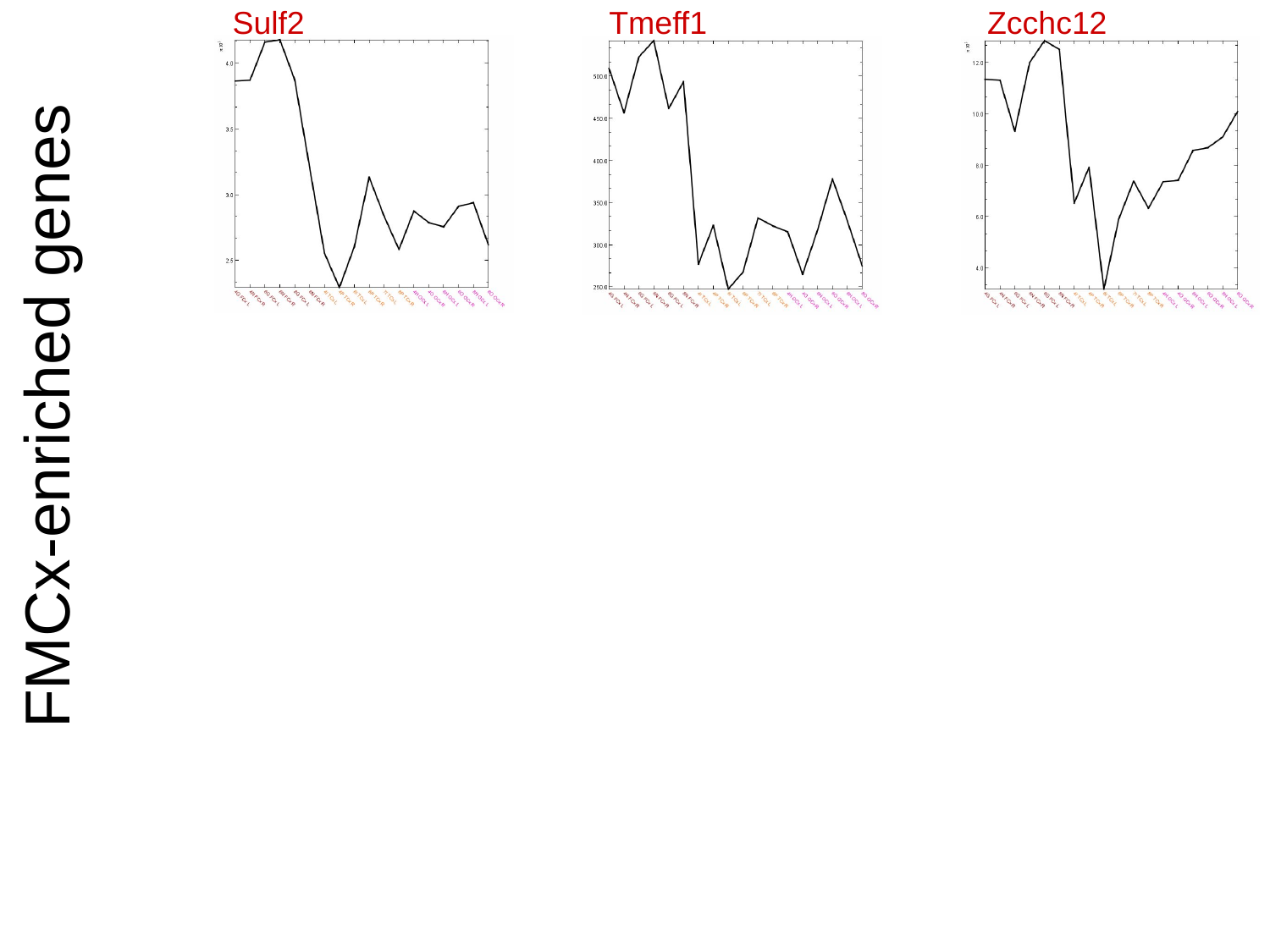

Sulf2
Tmeff1
Zcchc12
FMCx-enriched genes

## Slide 5
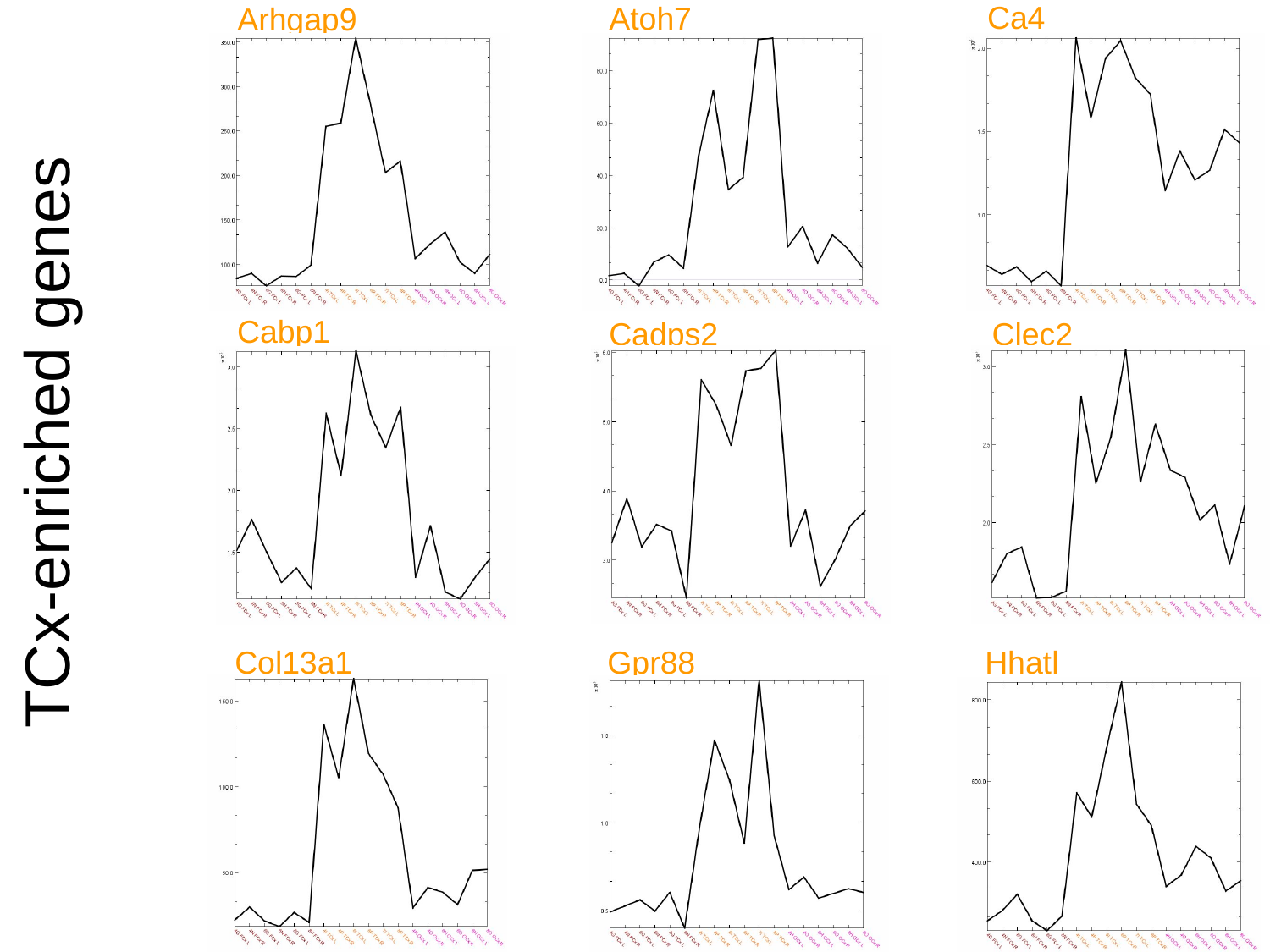

Ca4
Atoh7
Arhgap9
Cabp1
Cadps2
Clec2
TCx-enriched genes
Col13a1
Gpr88
Hhatl

## Slide 6
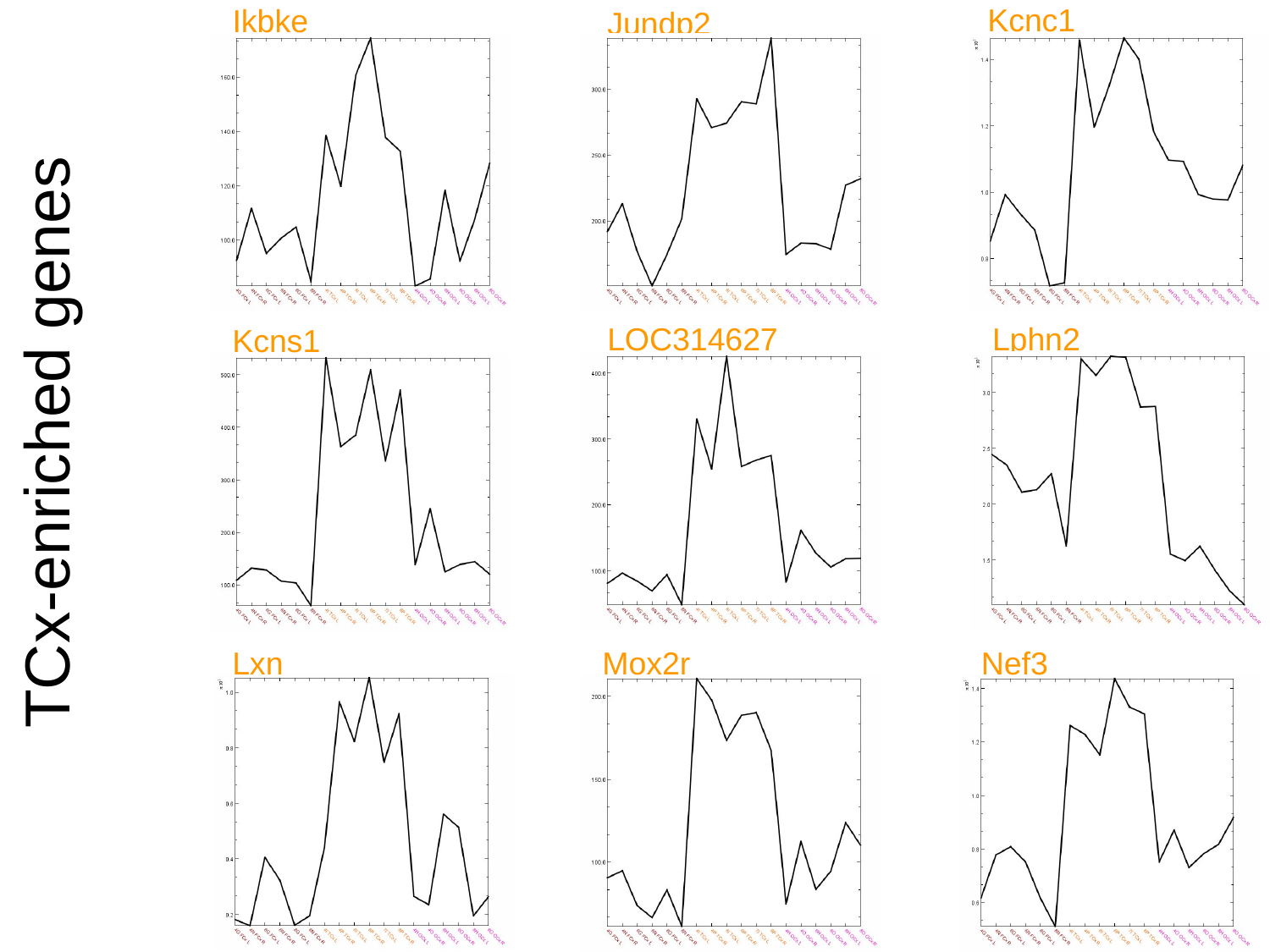

Kcnc1
Ikbke
Jundp2
LOC314627
Lphn2
Kcns1
TCx-enriched genes
Lxn
Mox2r
Nef3

## Slide 7
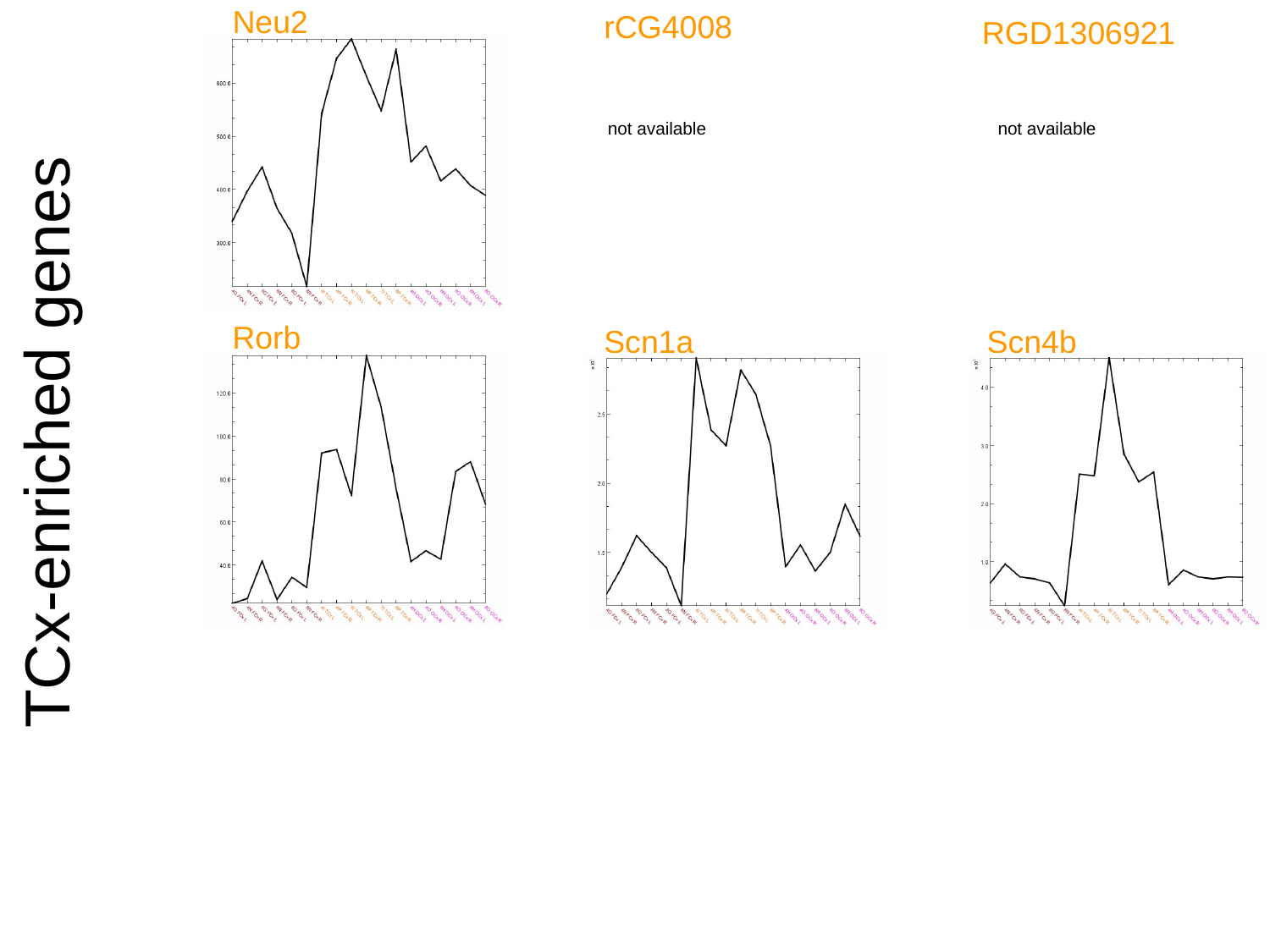

rCG4008
Neu2
RGD1306921
not available
not available
Rorb
Scn1a
Scn4b
TCx-enriched genes

## Slide 8
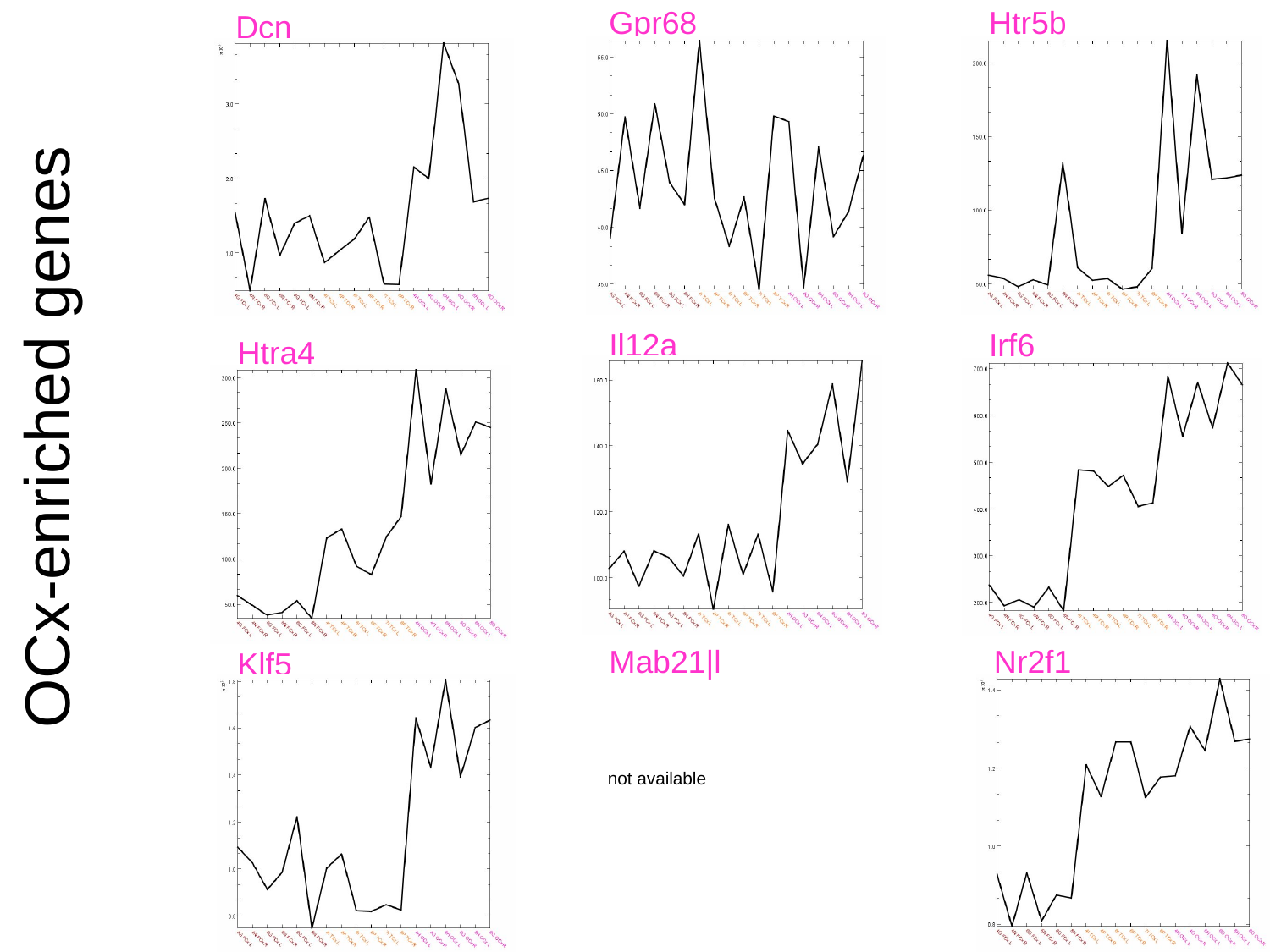

Gpr68
Htr5b
Dcn
Il12a
Irf6
Htra4
OCx-enriched genes
Mab21|l
Nr2f1
Klf5
not available

## Slide 9
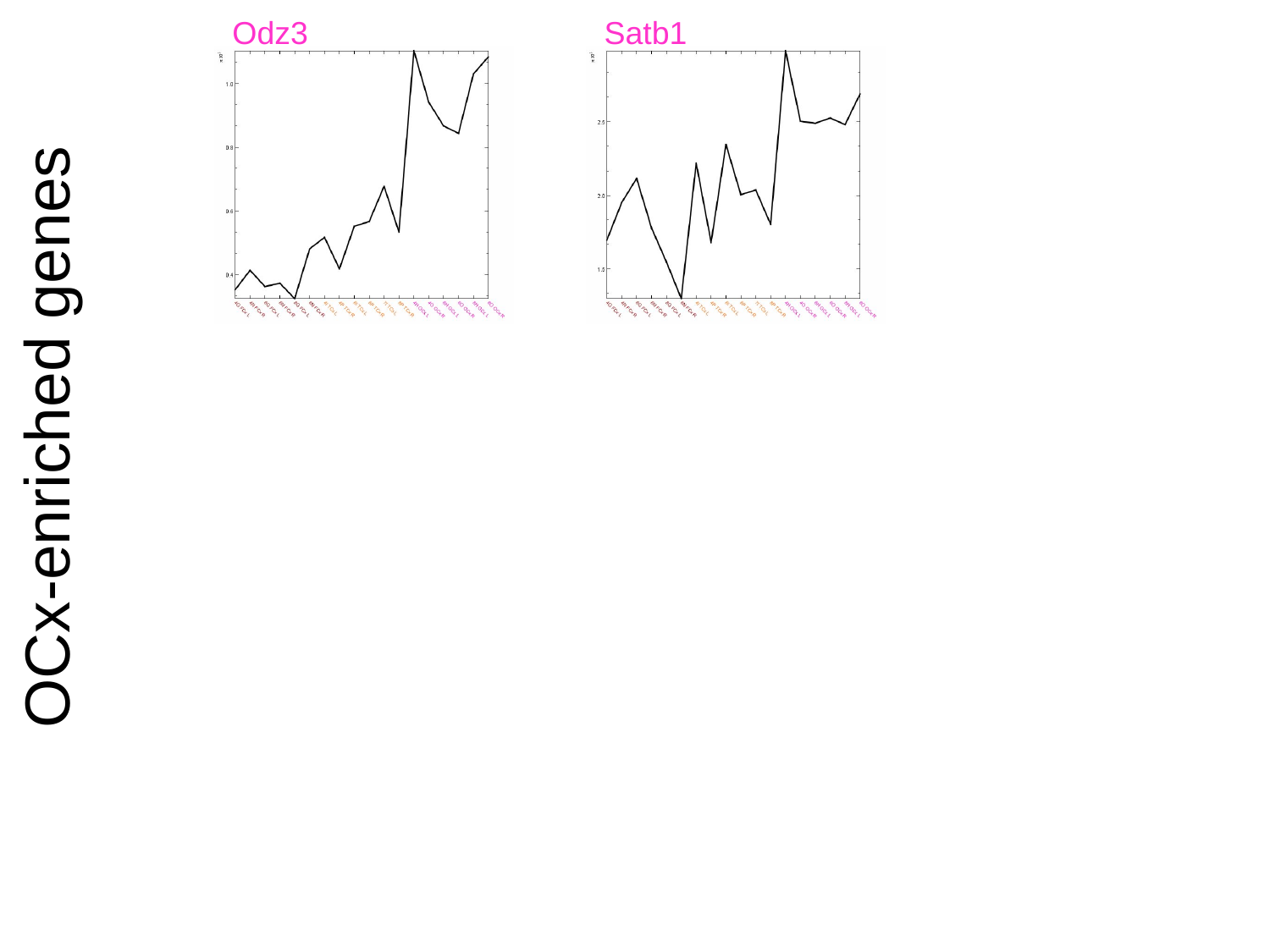

Odz3
Satb1
OCx-enriched genes
