# Supplementary material for: Gene expression in the rat brain: High similarity but unique differences between frontomedial-, temporal- and occipital cortex
Source: BMC Neurosci. 2011 Jan 26;12:15. doi: 10.1186/1471-2202-12-15 (PMC3040714; doi:10.1186/1471-2202-12-15)
Supplement: Additional file 6 — Gene expression of regionally enriched genes throughout development of the rat cerebral cortex. This file displays gene expression profiles of regionally enriched genes across pre- and postnatal stages of the developing rat cerebral cortex. Individual samples are placed along the x-axis; Cortex E16, E17, E18, E20, P01, P07, P14, P21, P30 and P90, Hippocampus P0, P07, P14, P21, P30 and P90, Hypothalamus E18, E20, P01, P07, P14, P21, P30 and P90; see original publication for details. The y-axis indicates quantile normalised signal intensities for each gene in each individual sample. Raw microarray data were obtained from [10]. 32 of our genes were represented in this data set. [file 1471-2202-12-15-S6.PPT]

## Slide 1
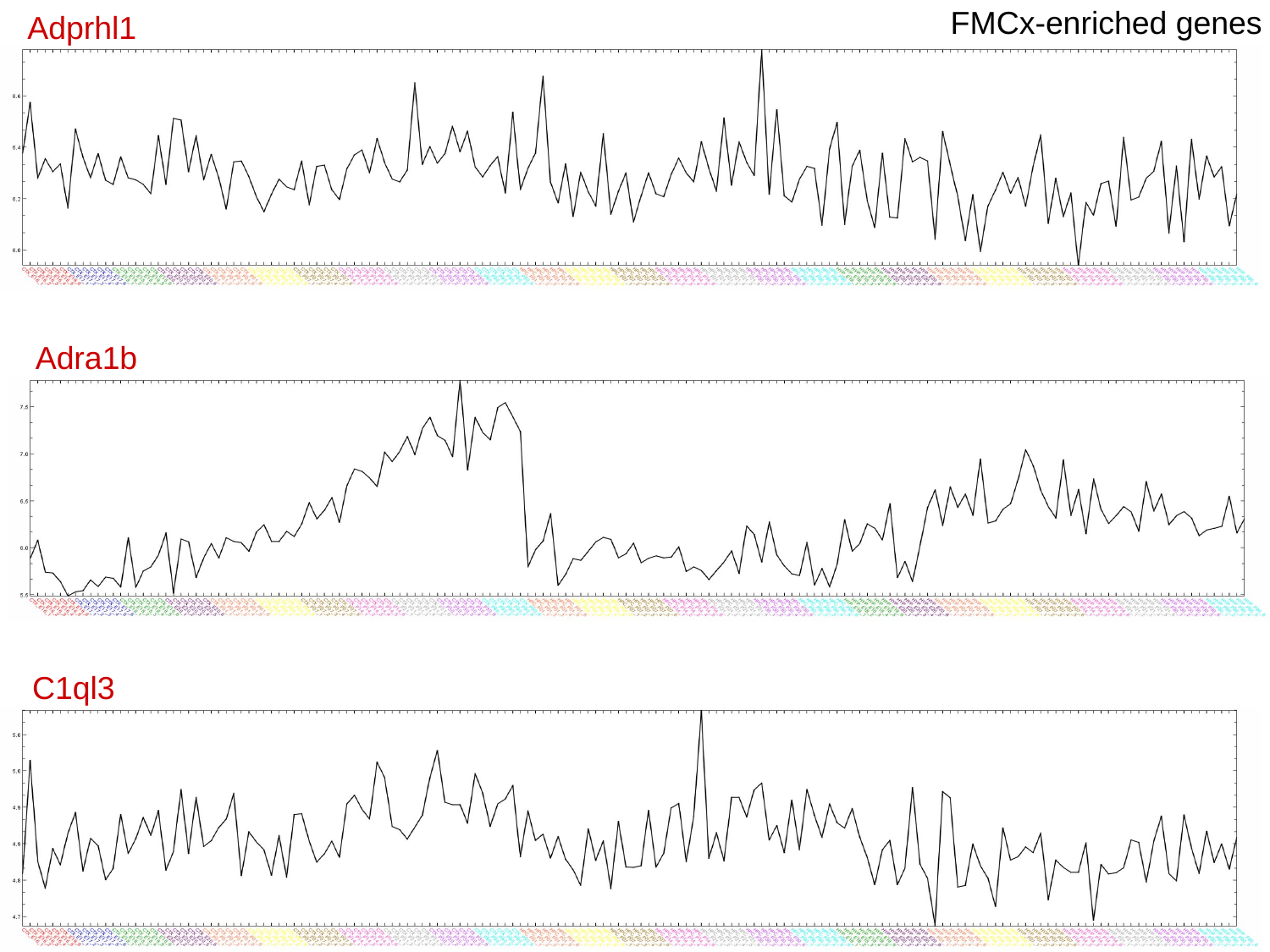

FMCx-enriched genes
Adprhl1
Adra1b
C1ql3

## Slide 2
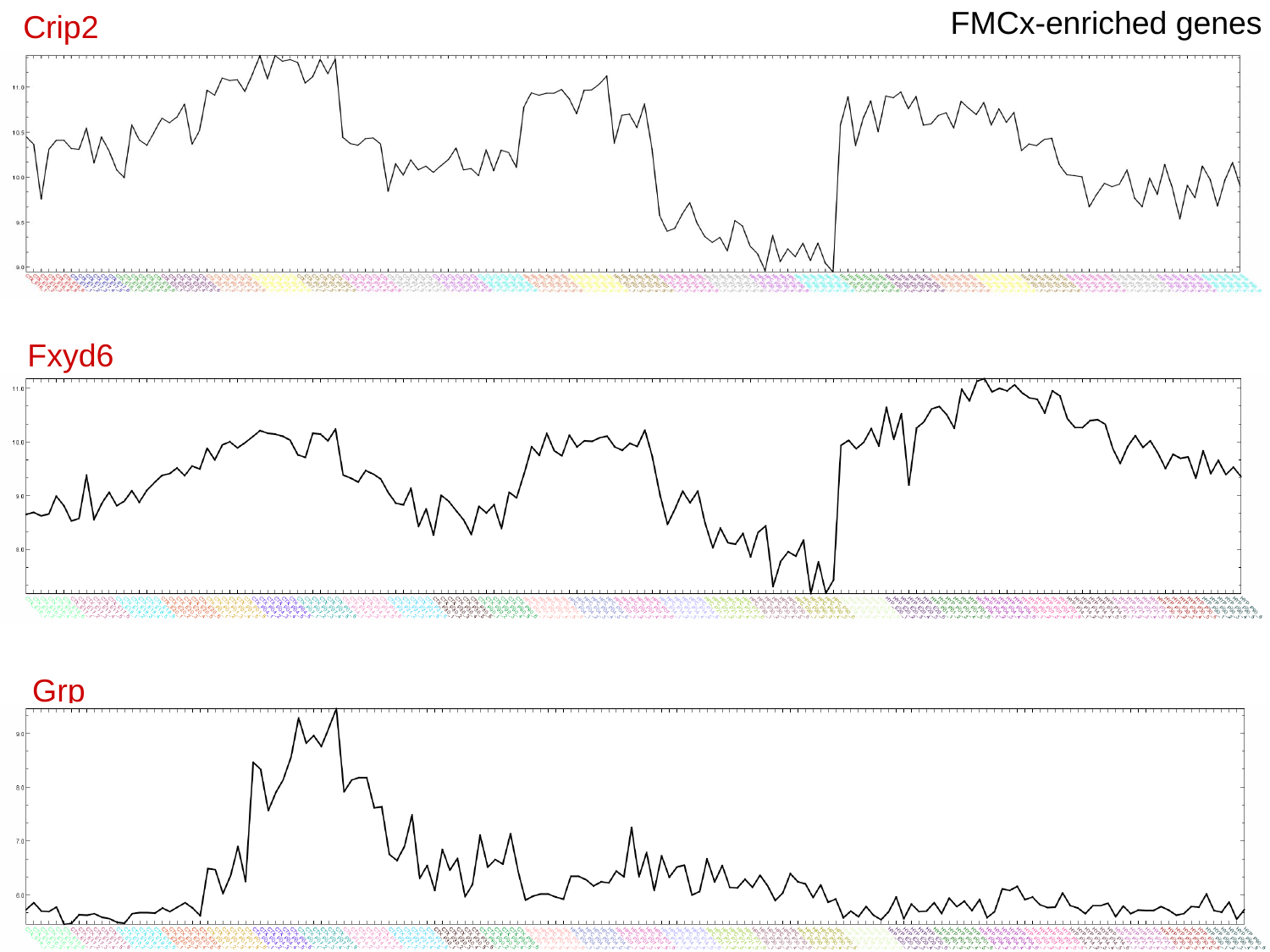

Crip2
FMCx-enriched genes
Fxyd6
Grp

## Slide 3
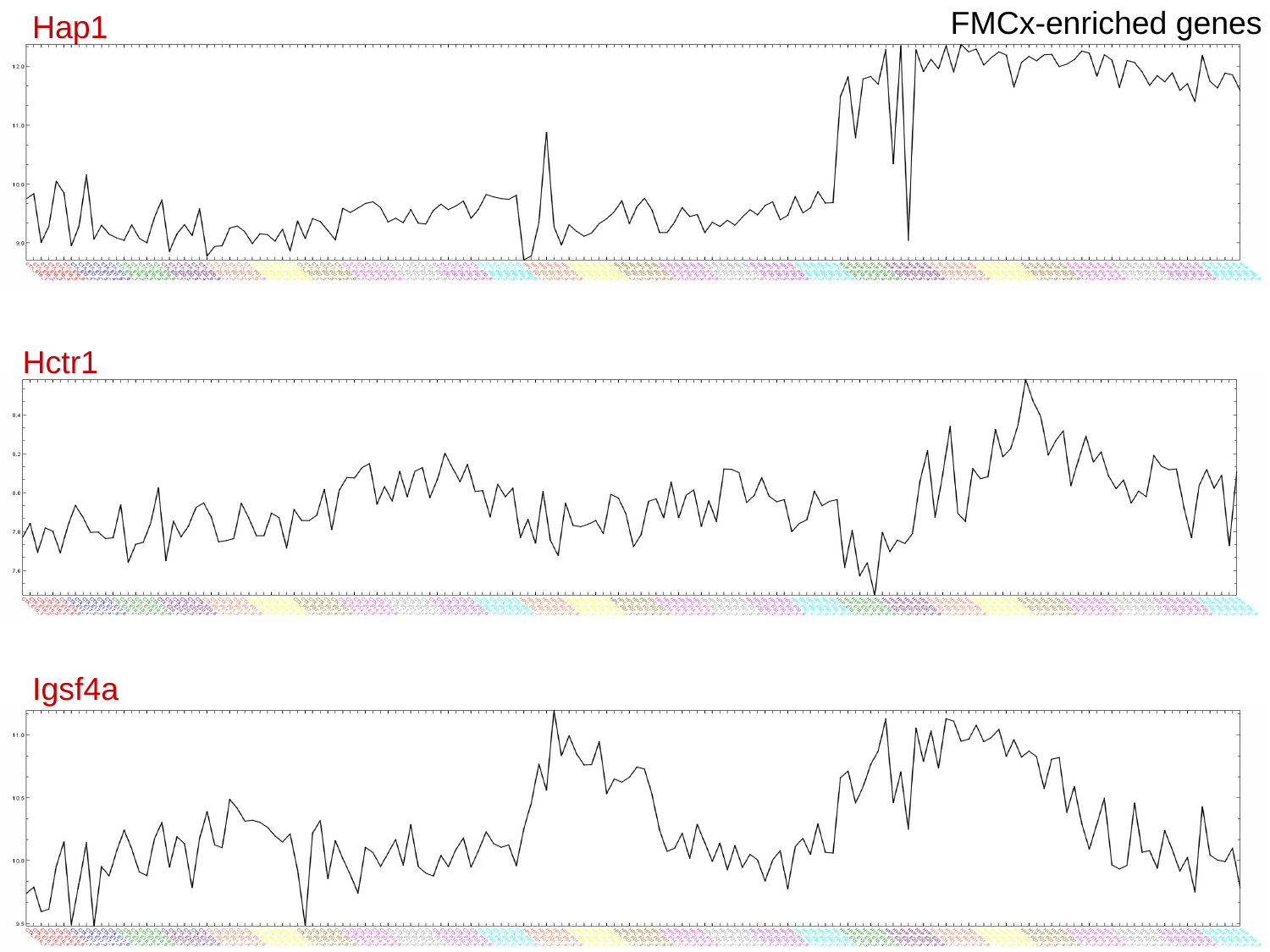

Hap1
FMCx-enriched genes
Hctr1
Igsf4a

## Slide 4
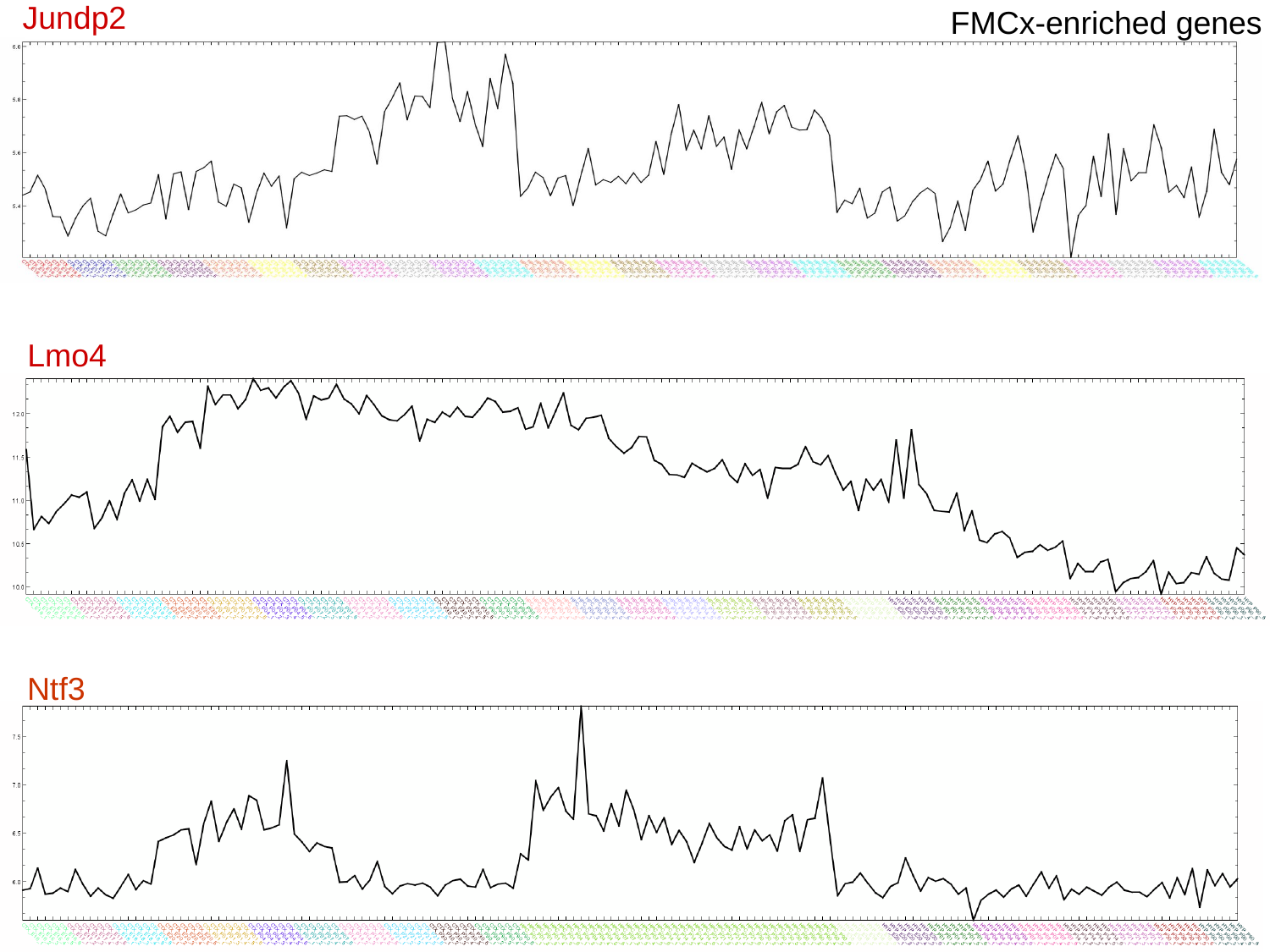

Jundp2
FMCx-enriched genes
Lmo4
Ntf3

## Slide 5
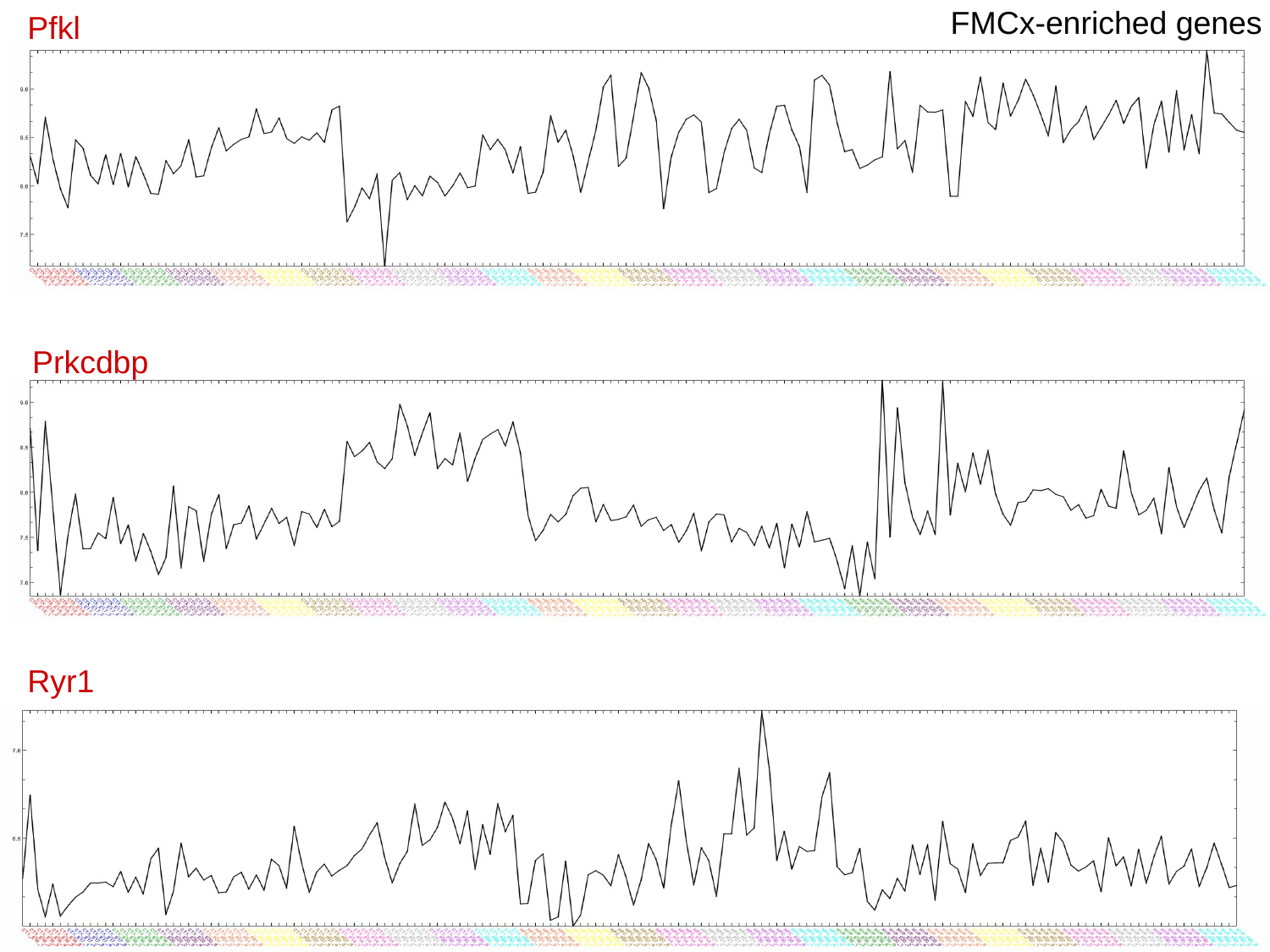

FMCx-enriched genes
Pfkl
Prkcdbp
Ryr1

## Slide 6
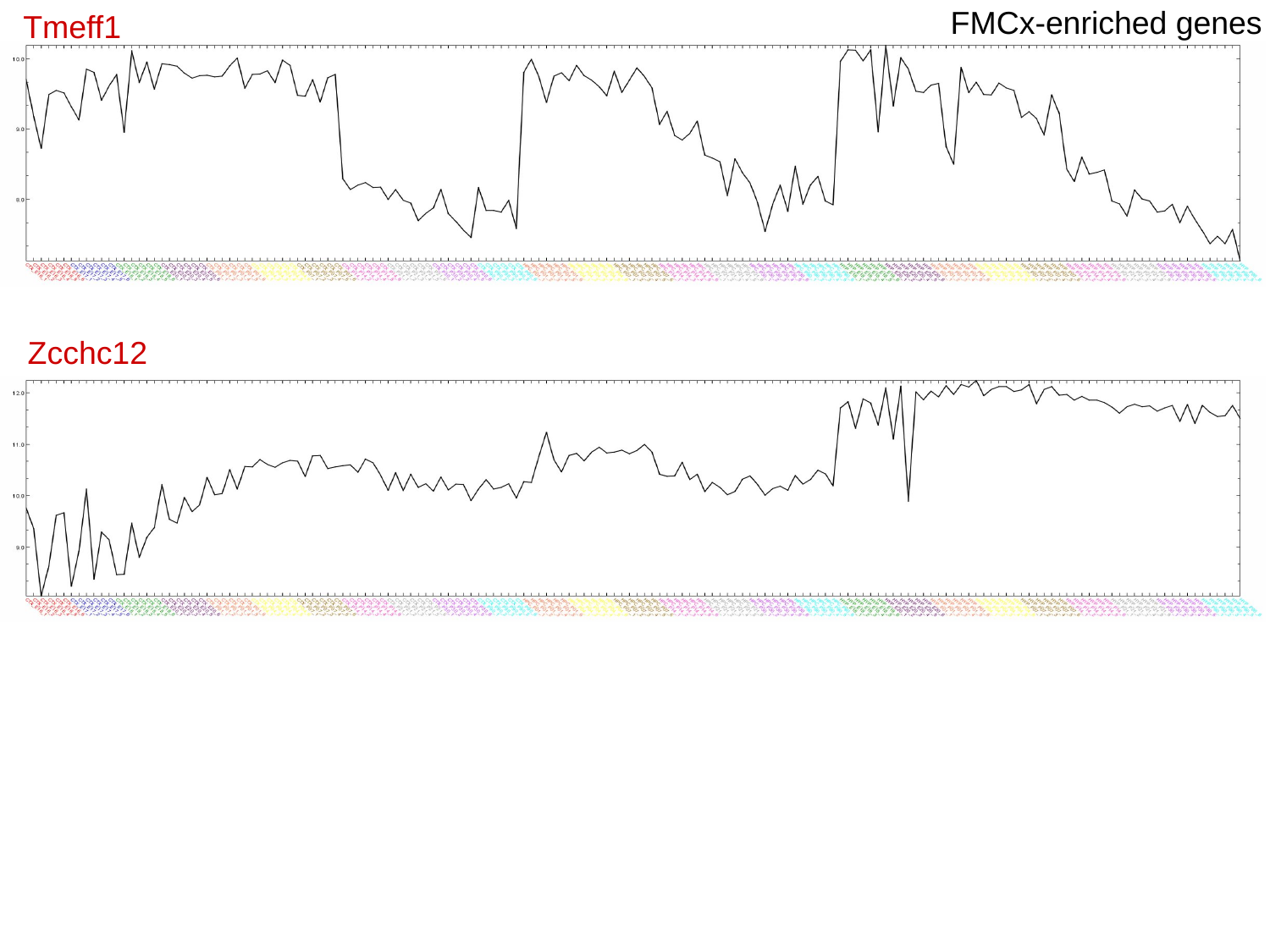

Tmeff1
FMCx-enriched genes
Zcchc12

## Slide 7
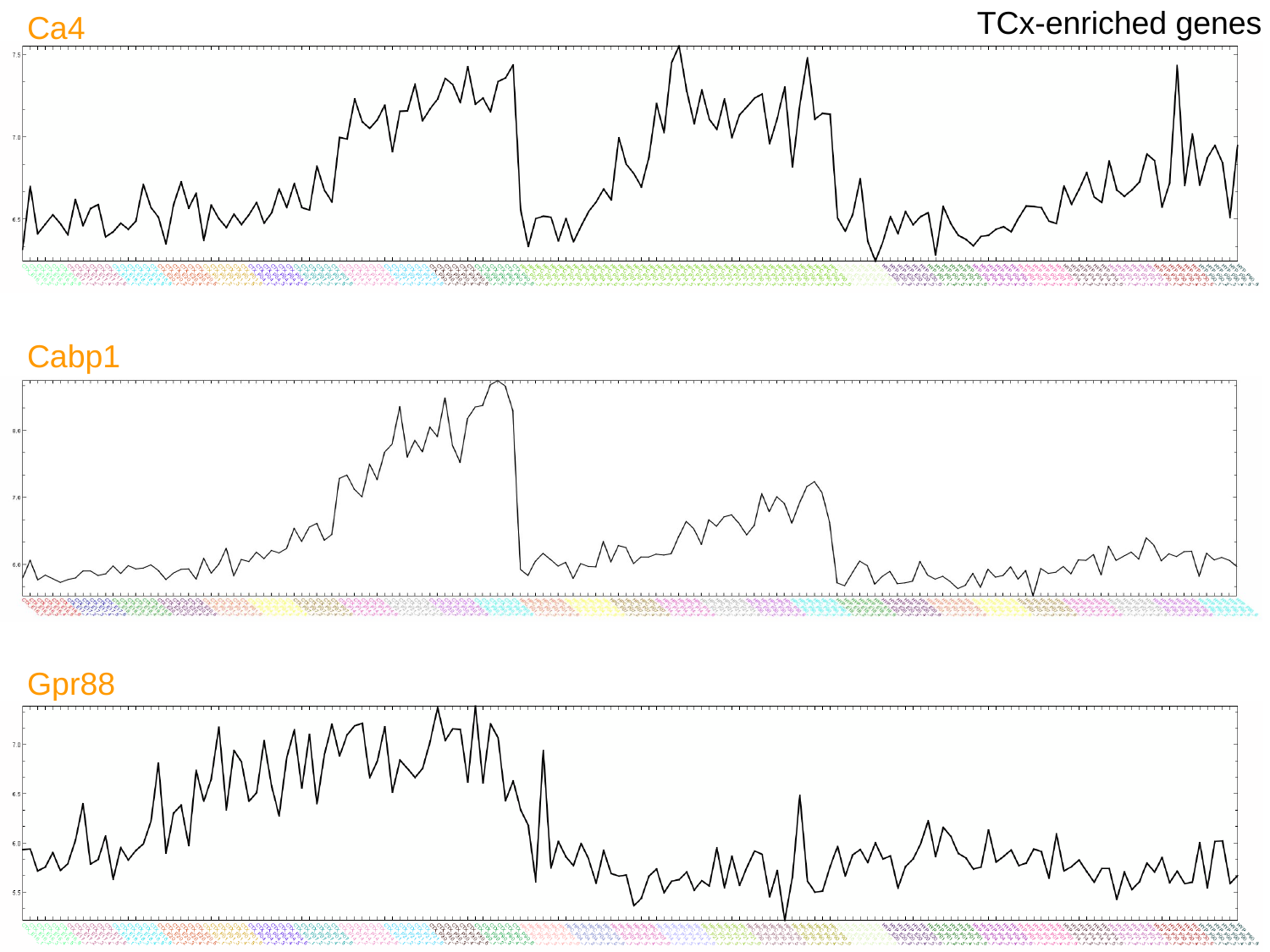

TCx-enriched genes
Ca4
Cabp1
Gpr88

## Slide 8
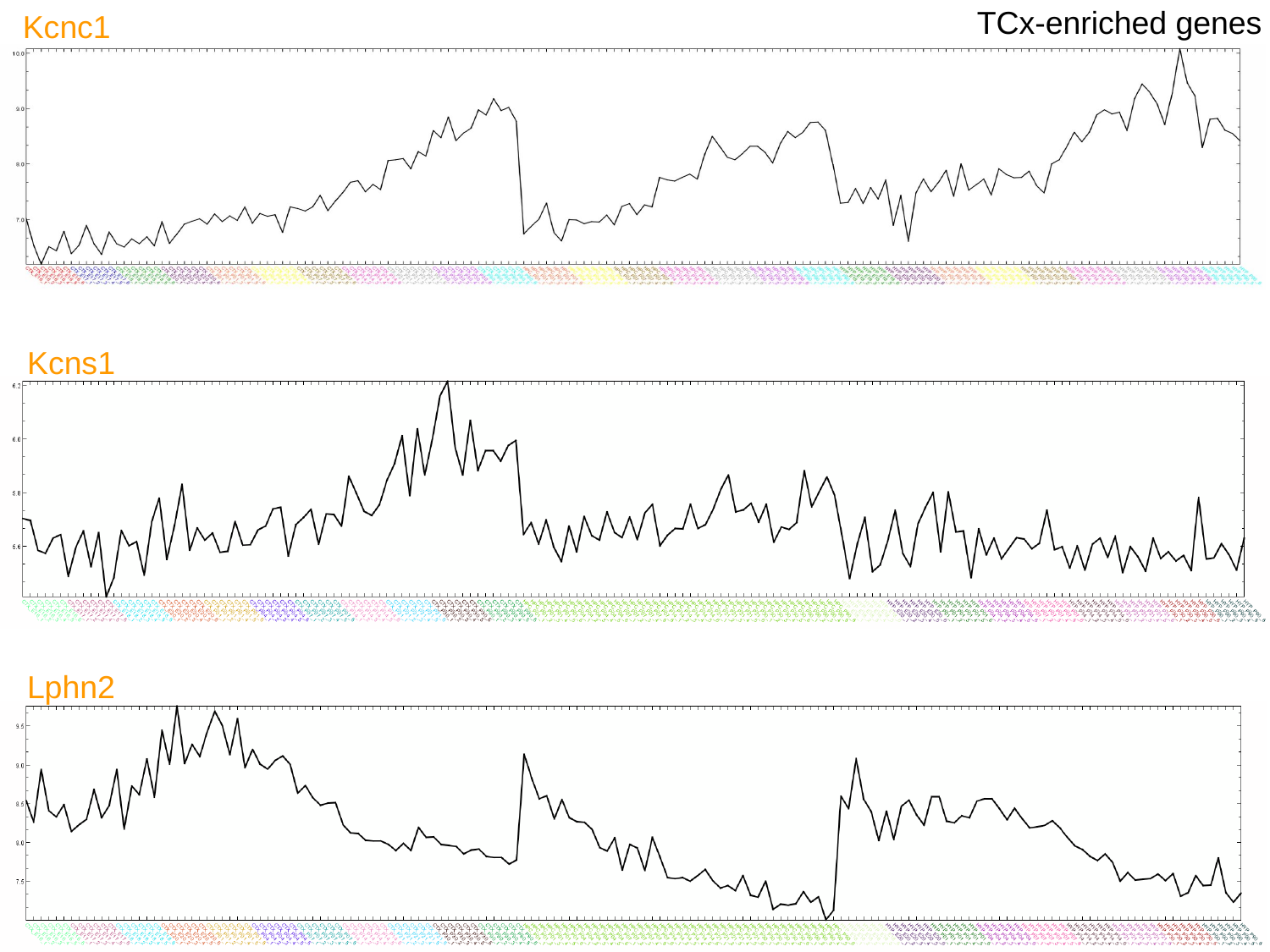

Kcnc1
TCx-enriched genes
Kcns1
Lphn2

## Slide 9
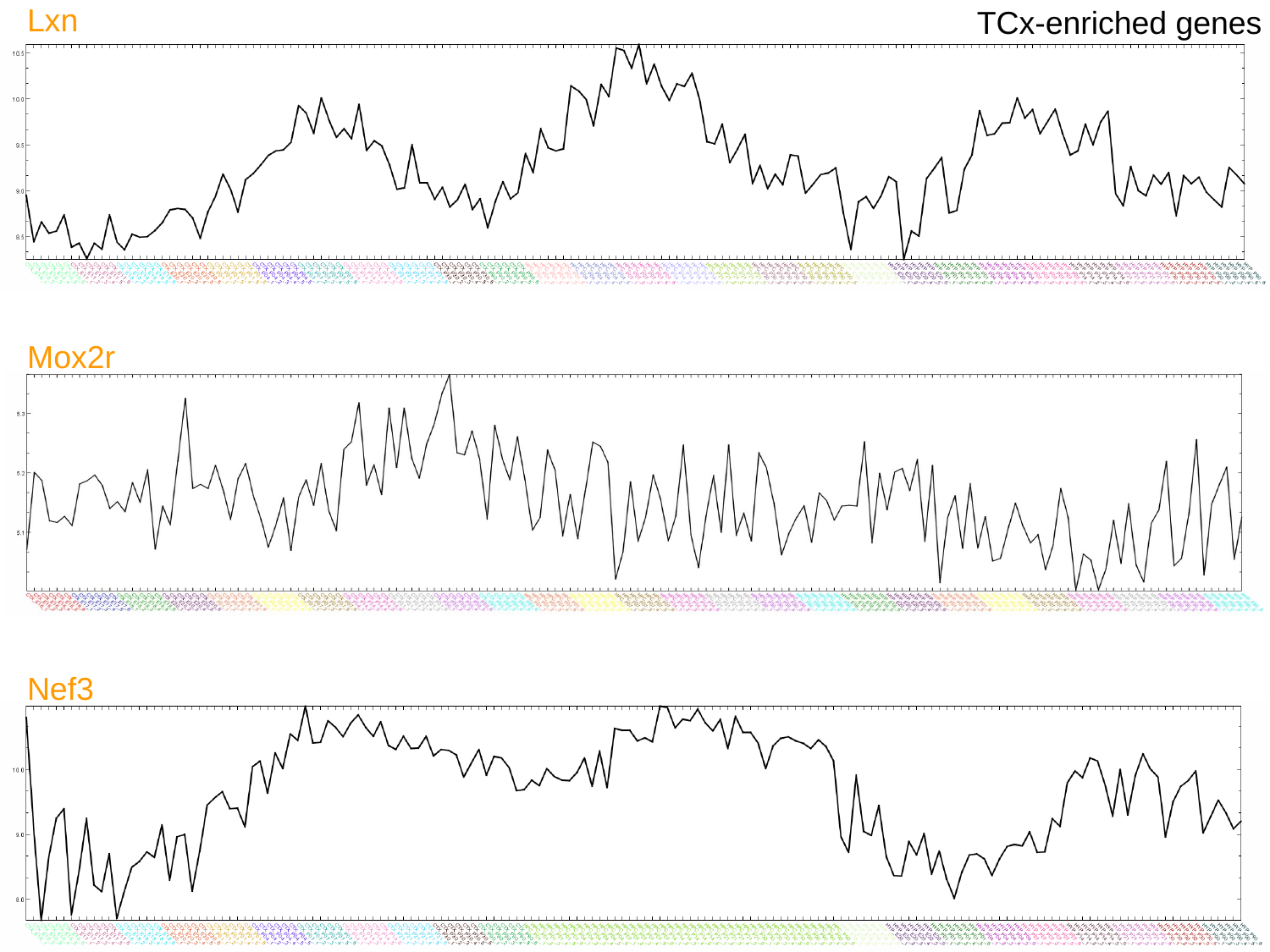

Lxn
TCx-enriched genes
Mox2r
Nef3

## Slide 10
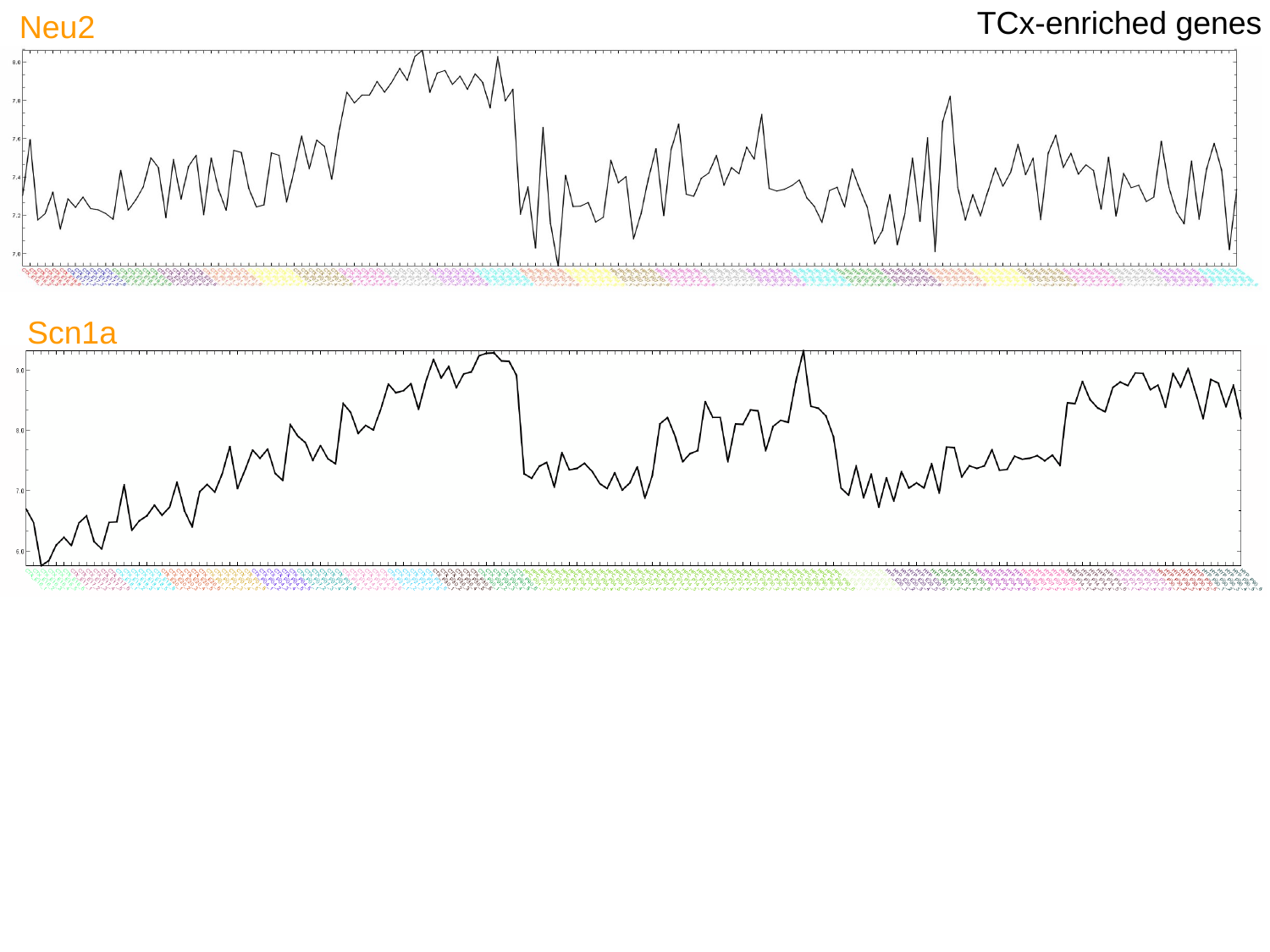

Neu2
TCx-enriched genes
Scn1a

## Slide 11
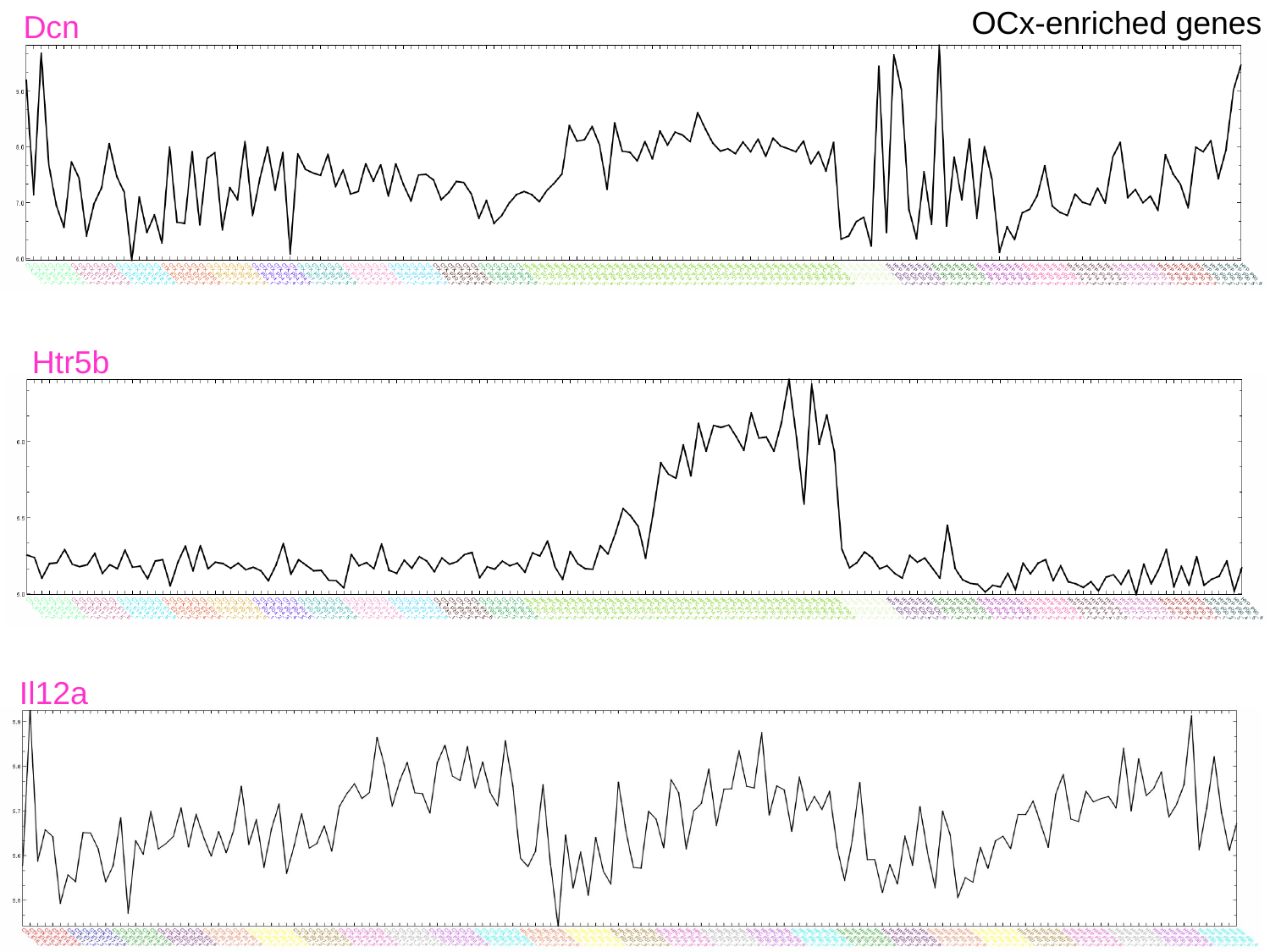

Dcn
OCx-enriched genes
Htr5b
Il12a

## Slide 12
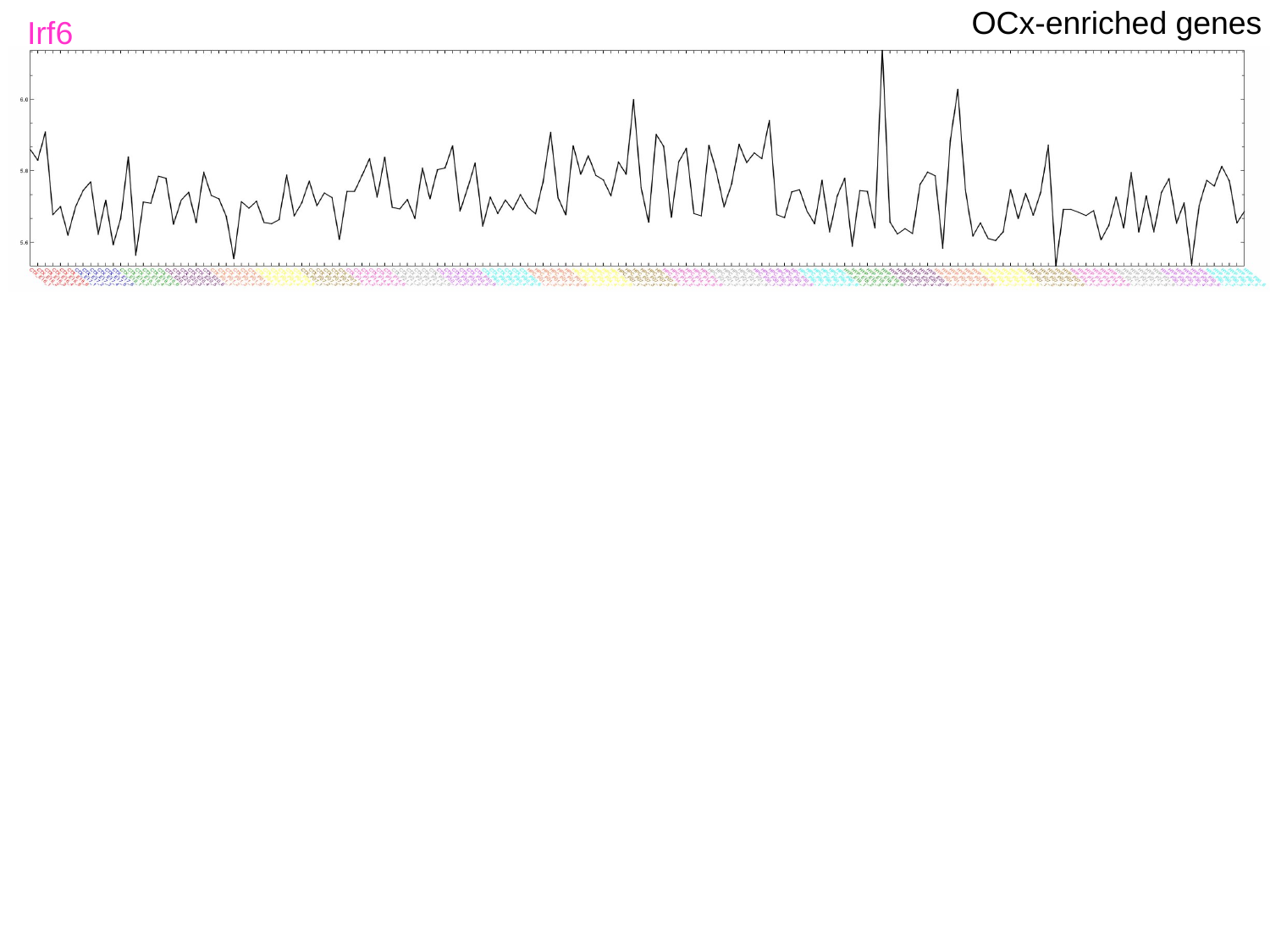

OCx-enriched genes
Irf6

## Slide 13
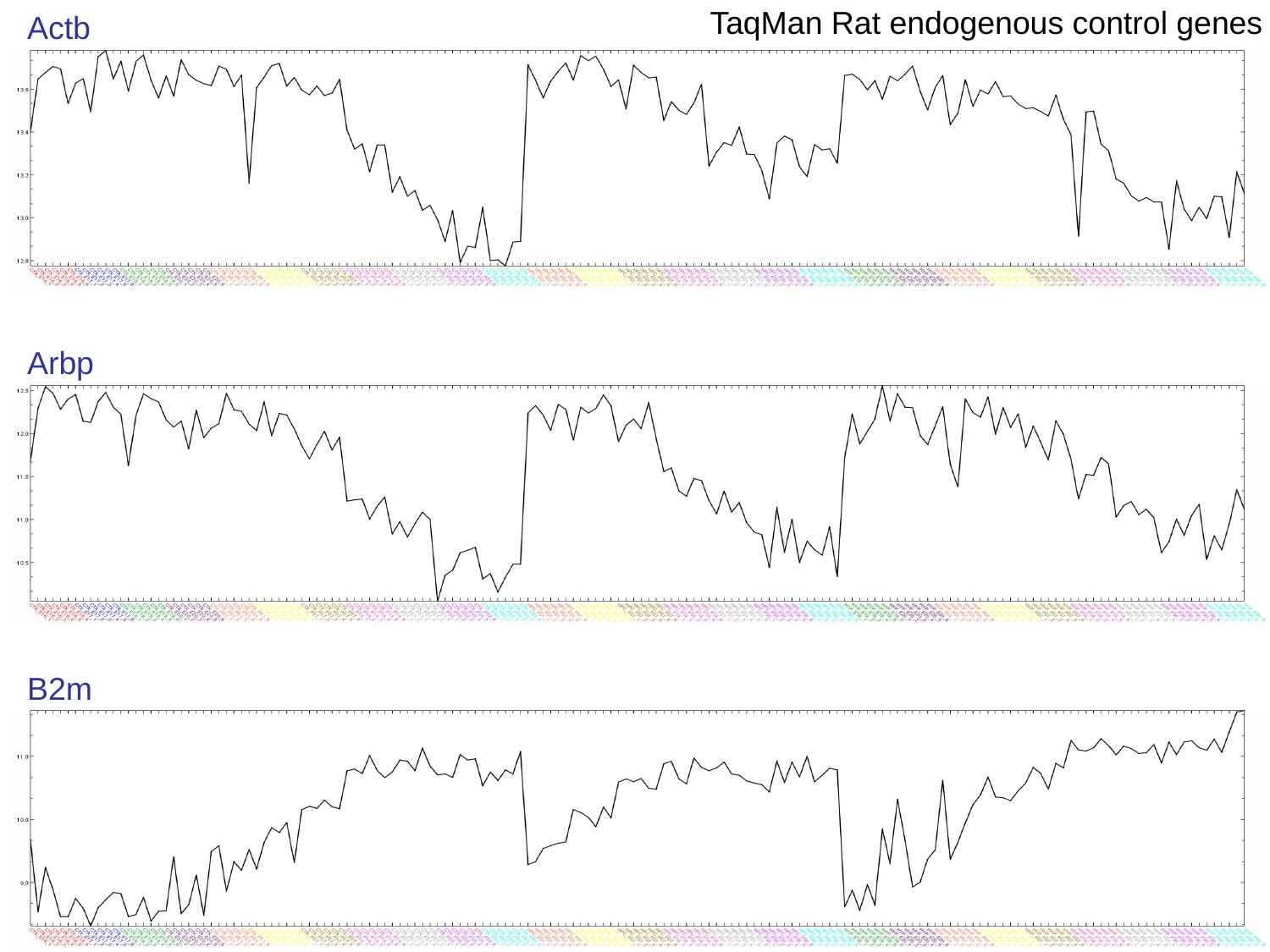

TaqMan Rat endogenous control genes
Actb
Arbp
B2m

## Slide 14
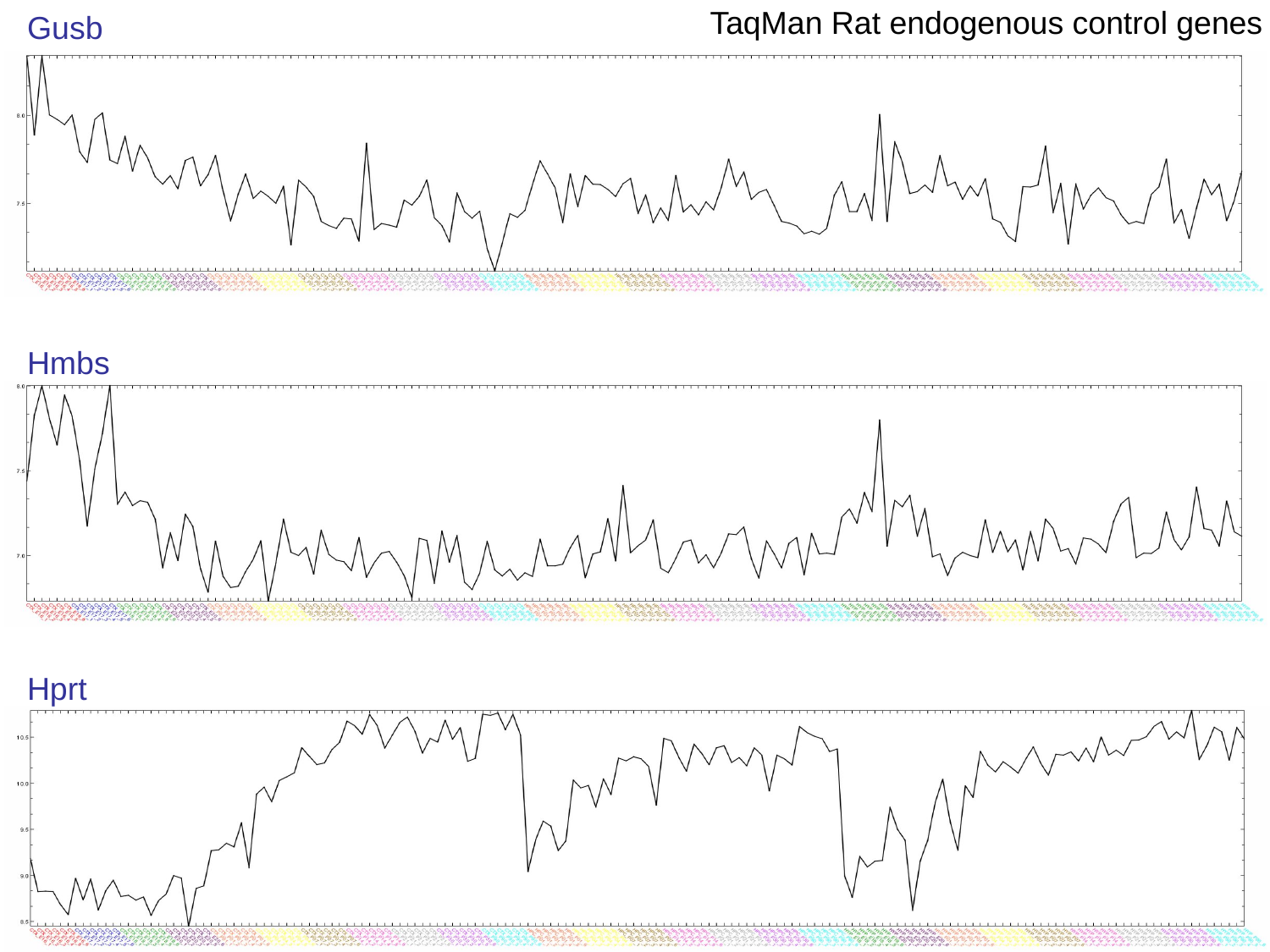

TaqMan Rat endogenous control genes
Gusb
Hmbs
Hprt

## Slide 15
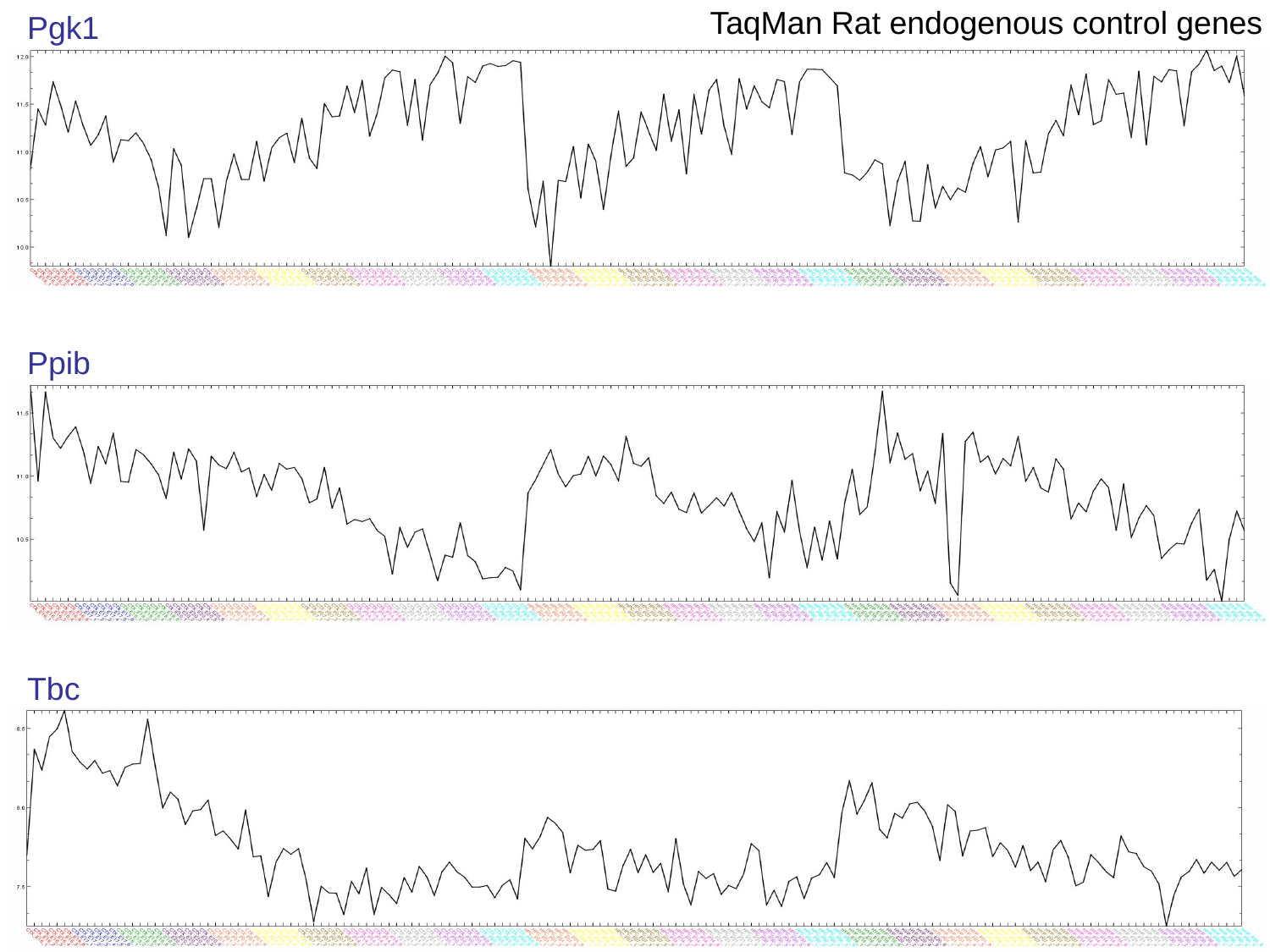

TaqMan Rat endogenous control genes
Pgk1
Ppib
Tbc

## Slide 16
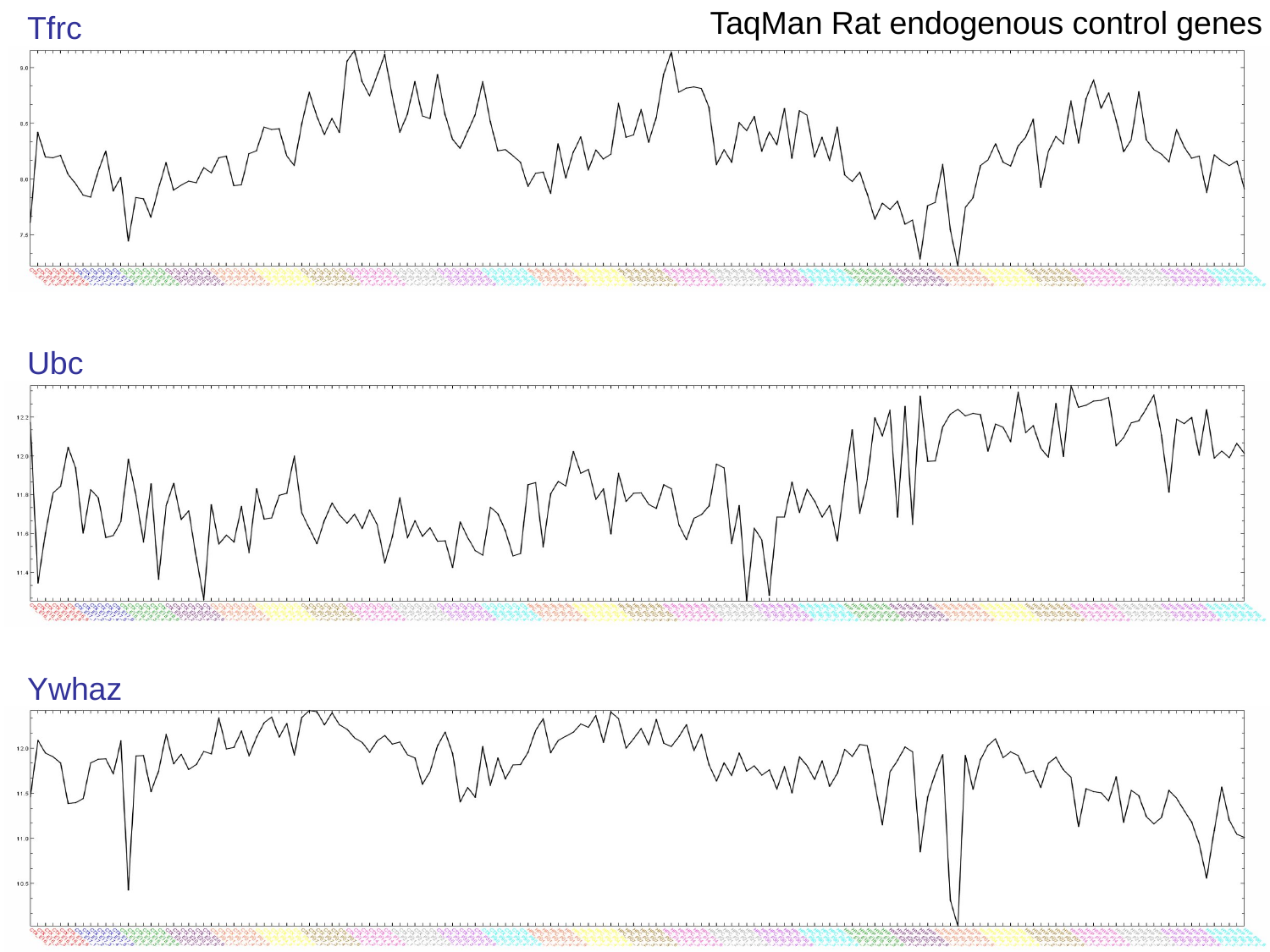

TaqMan Rat endogenous control genes
Tfrc
Ubc
Ywhaz

## Slide 17
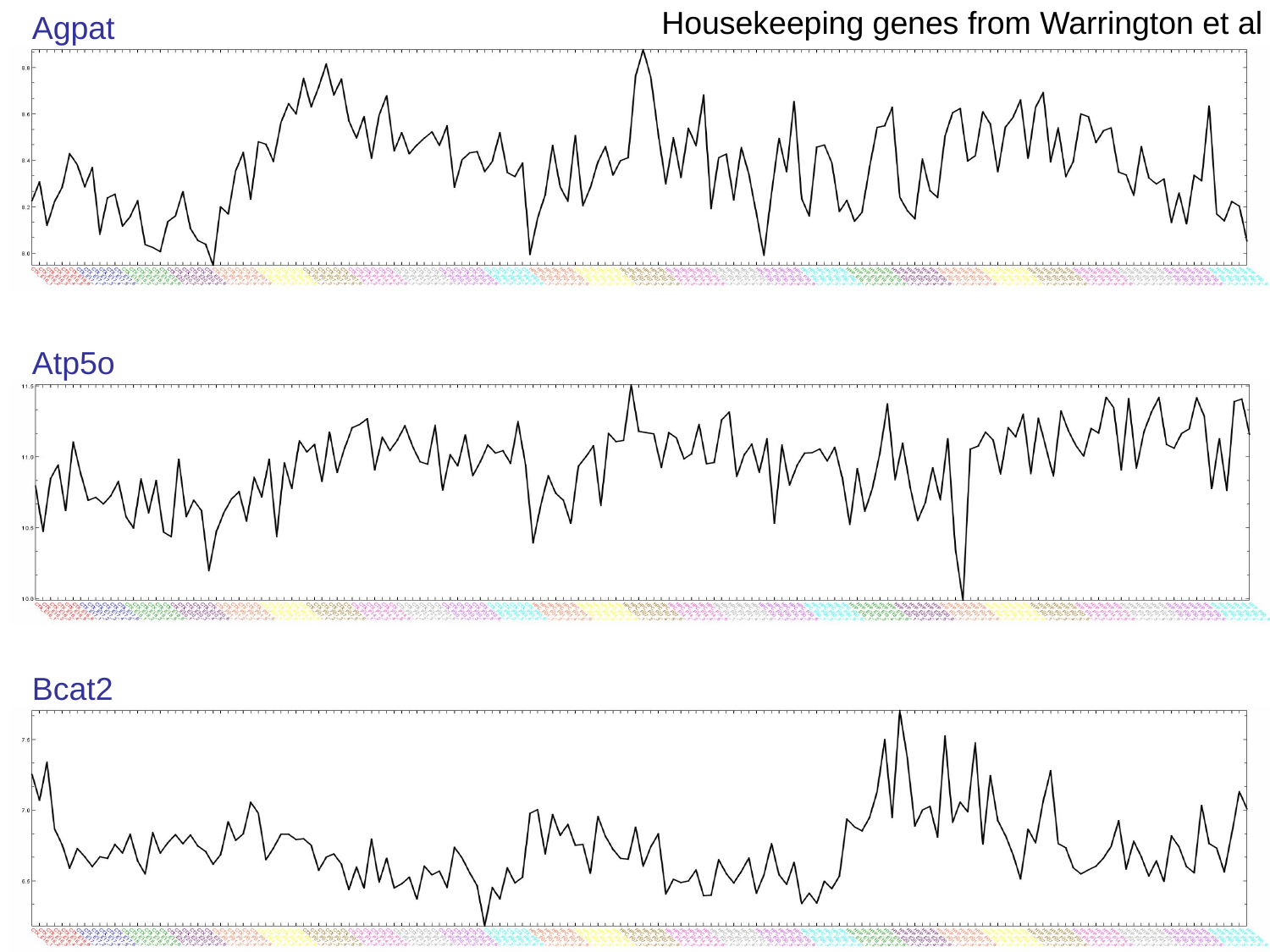

Housekeeping genes from Warrington et al
Agpat
Atp5o
Bcat2

## Slide 18
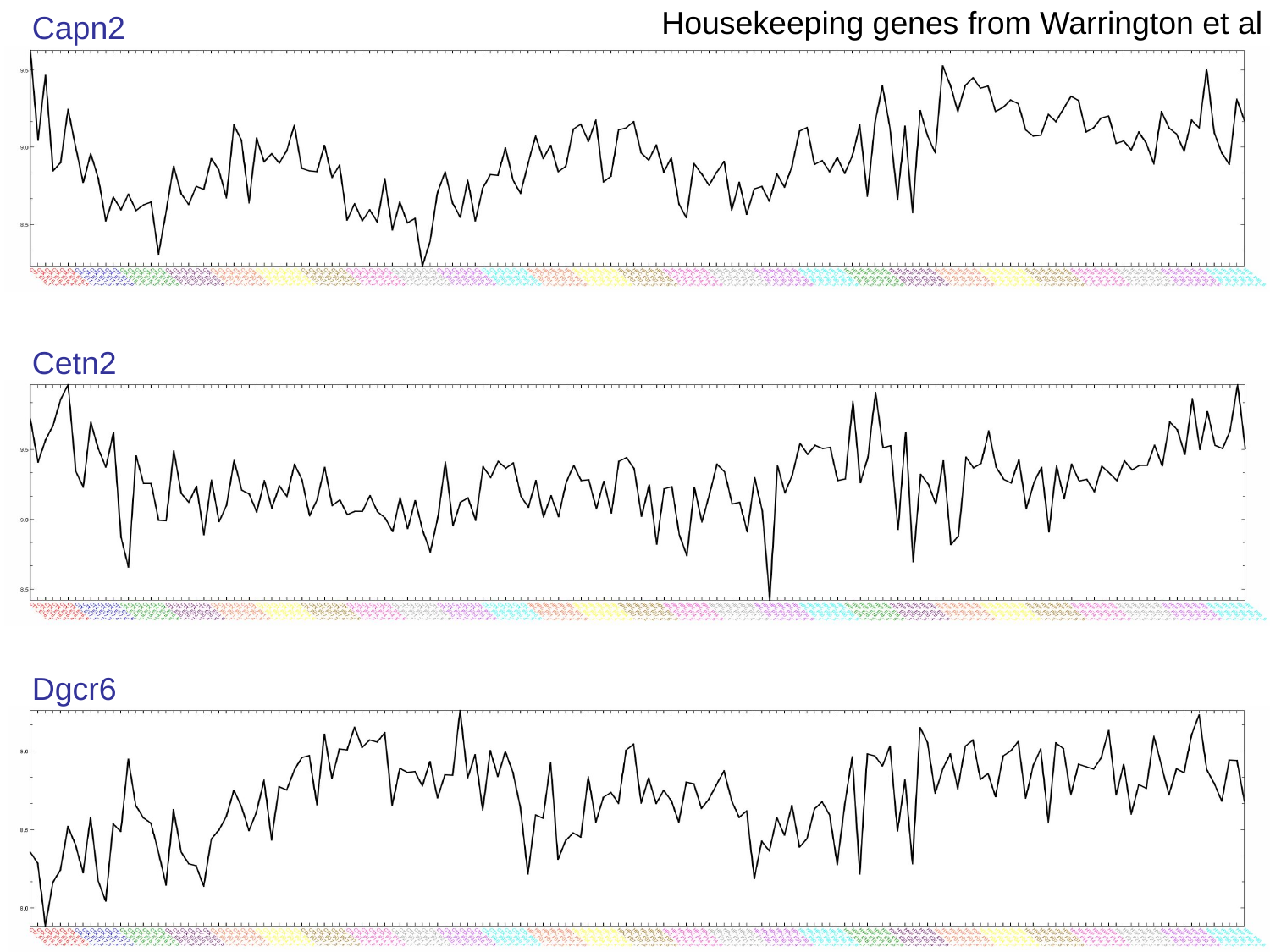

Housekeeping genes from Warrington et al
Capn2
Cetn2
Dgcr6

## Slide 19
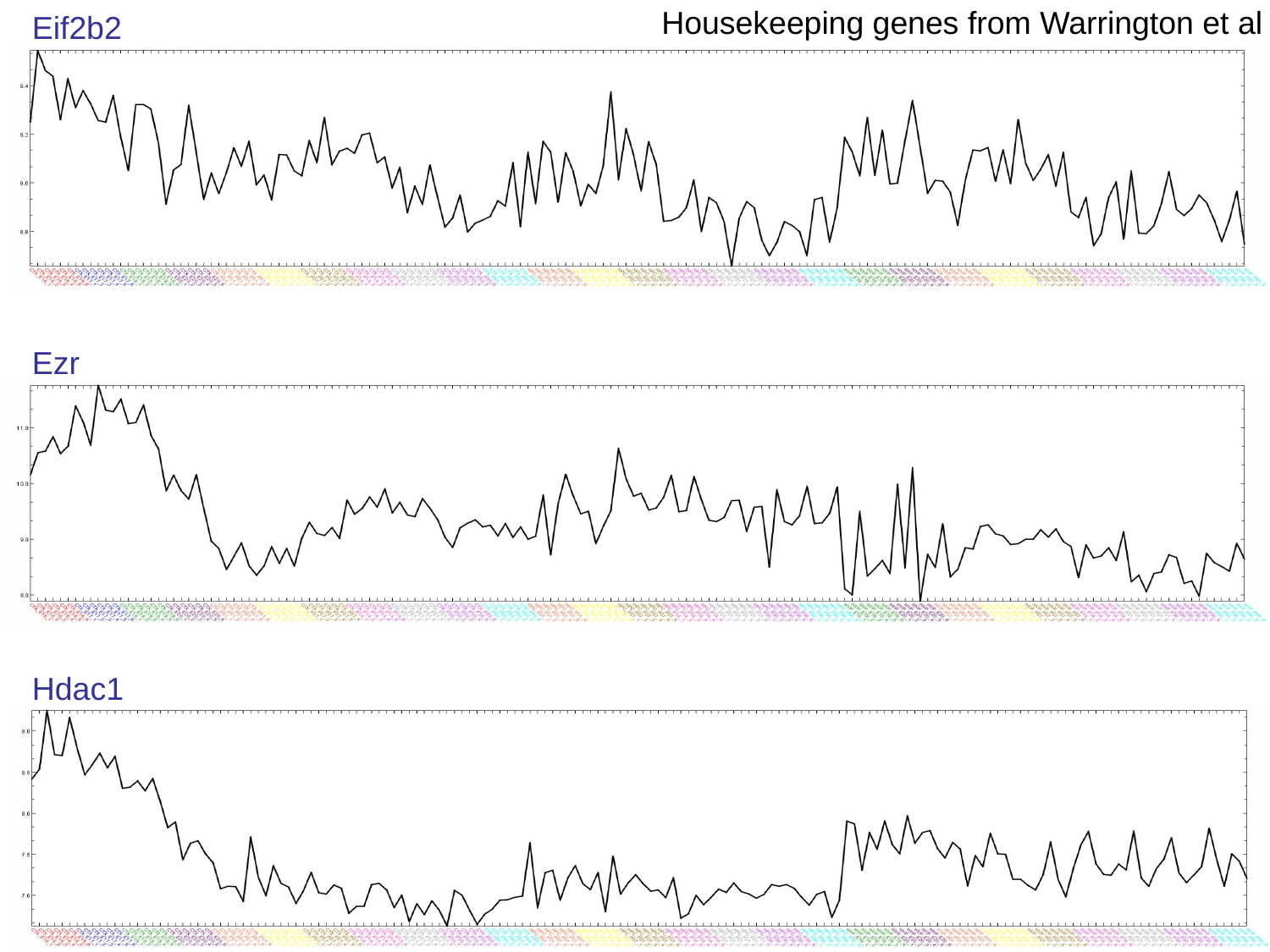

Housekeeping genes from Warrington et al
Eif2b2
Ezr
Hdac1

## Slide 20
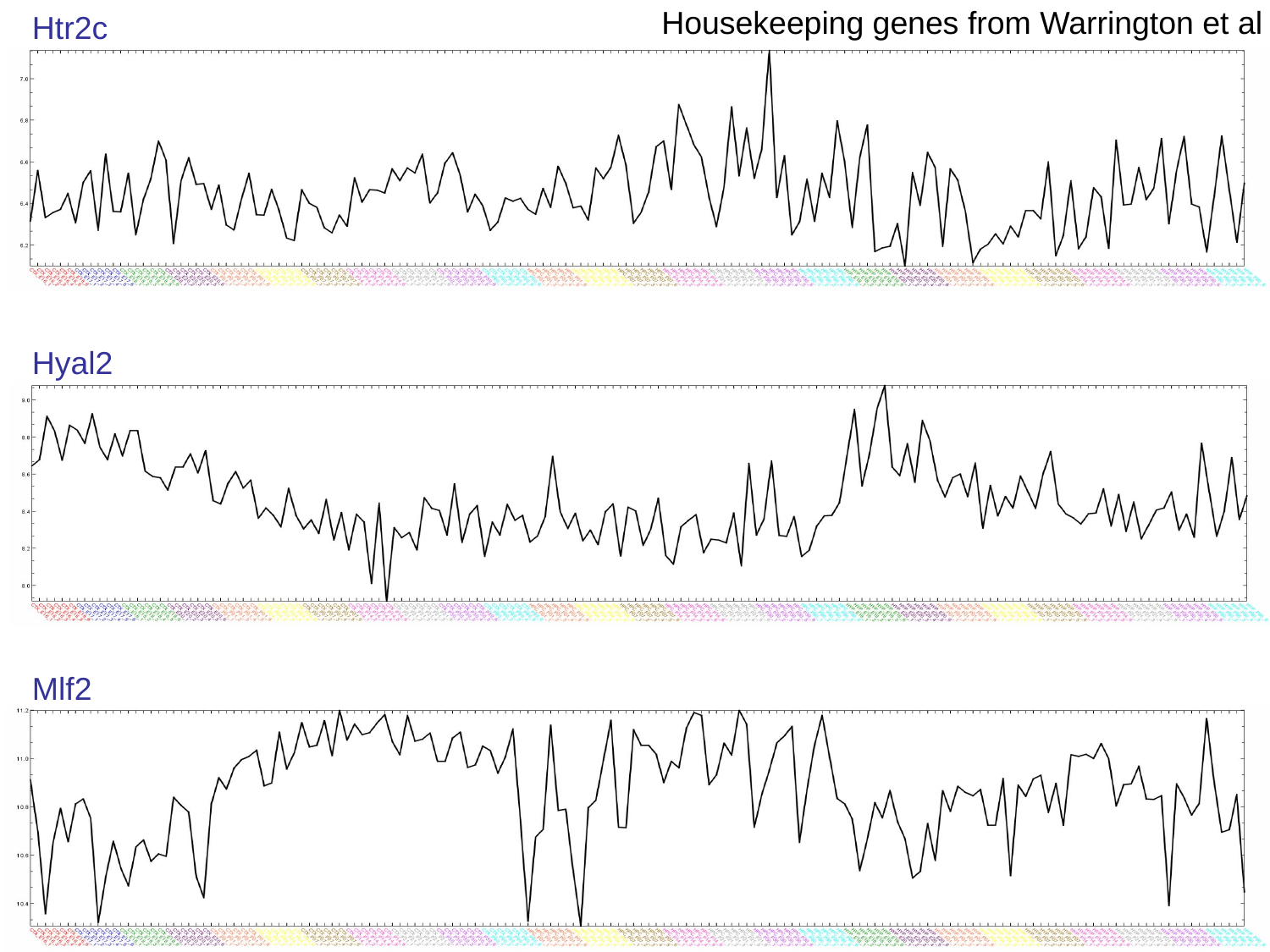

Housekeeping genes from Warrington et al
Htr2c
Hyal2
Mlf2

## Slide 21
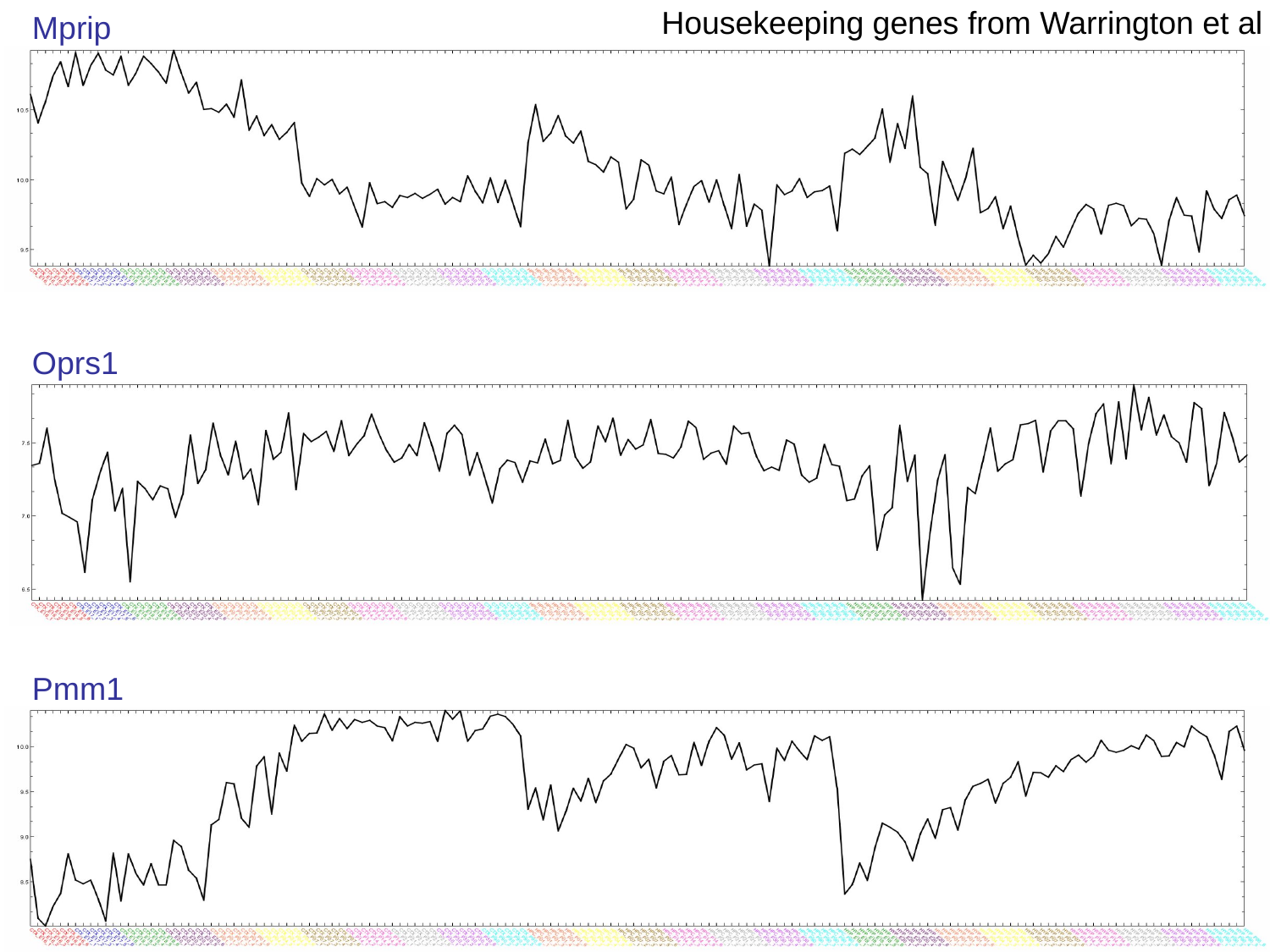

Housekeeping genes from Warrington et al
Mprip
Oprs1
Pmm1

## Slide 22
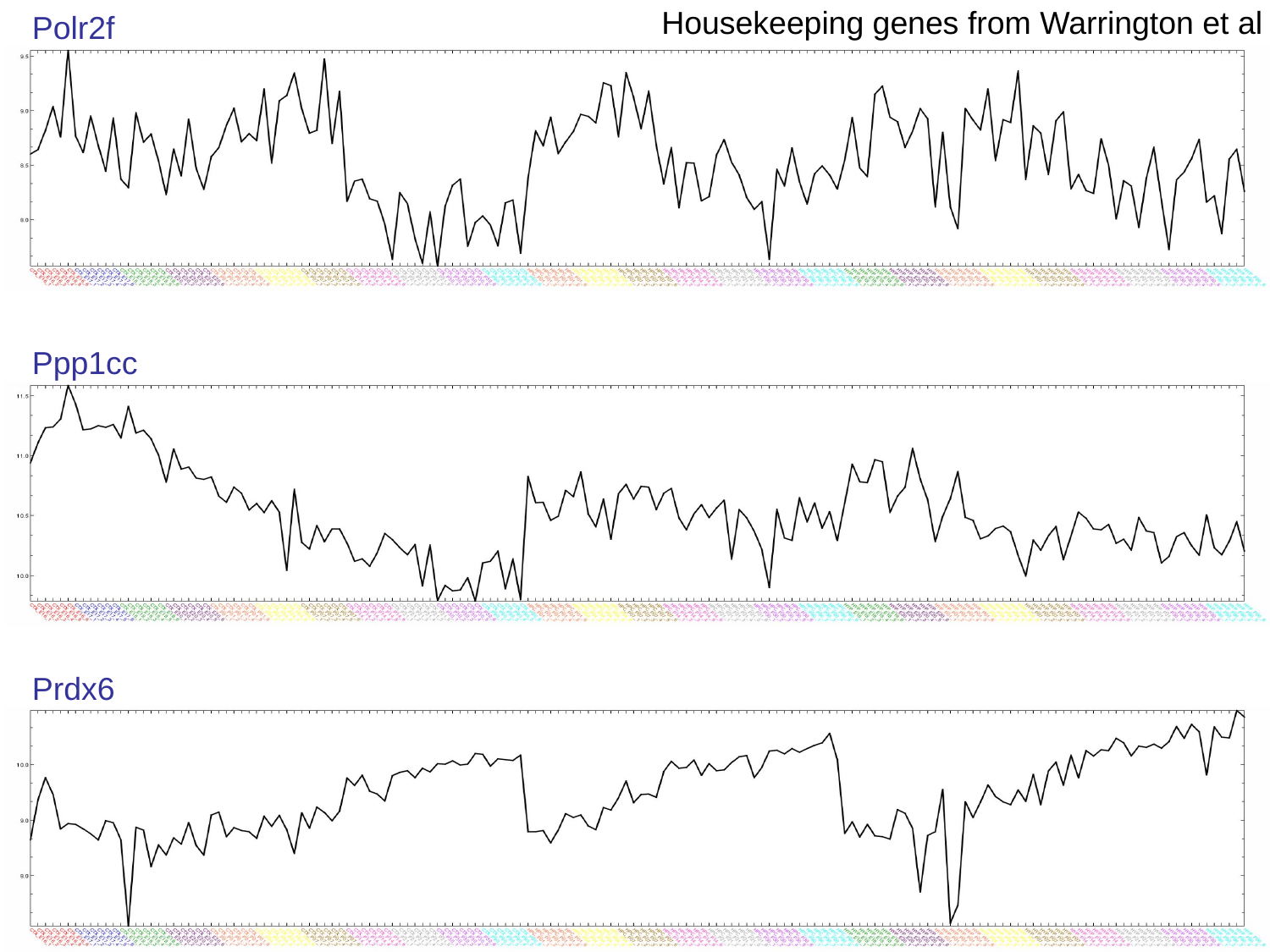

Housekeeping genes from Warrington et al
Polr2f
Ppp1cc
Prdx6

## Slide 23
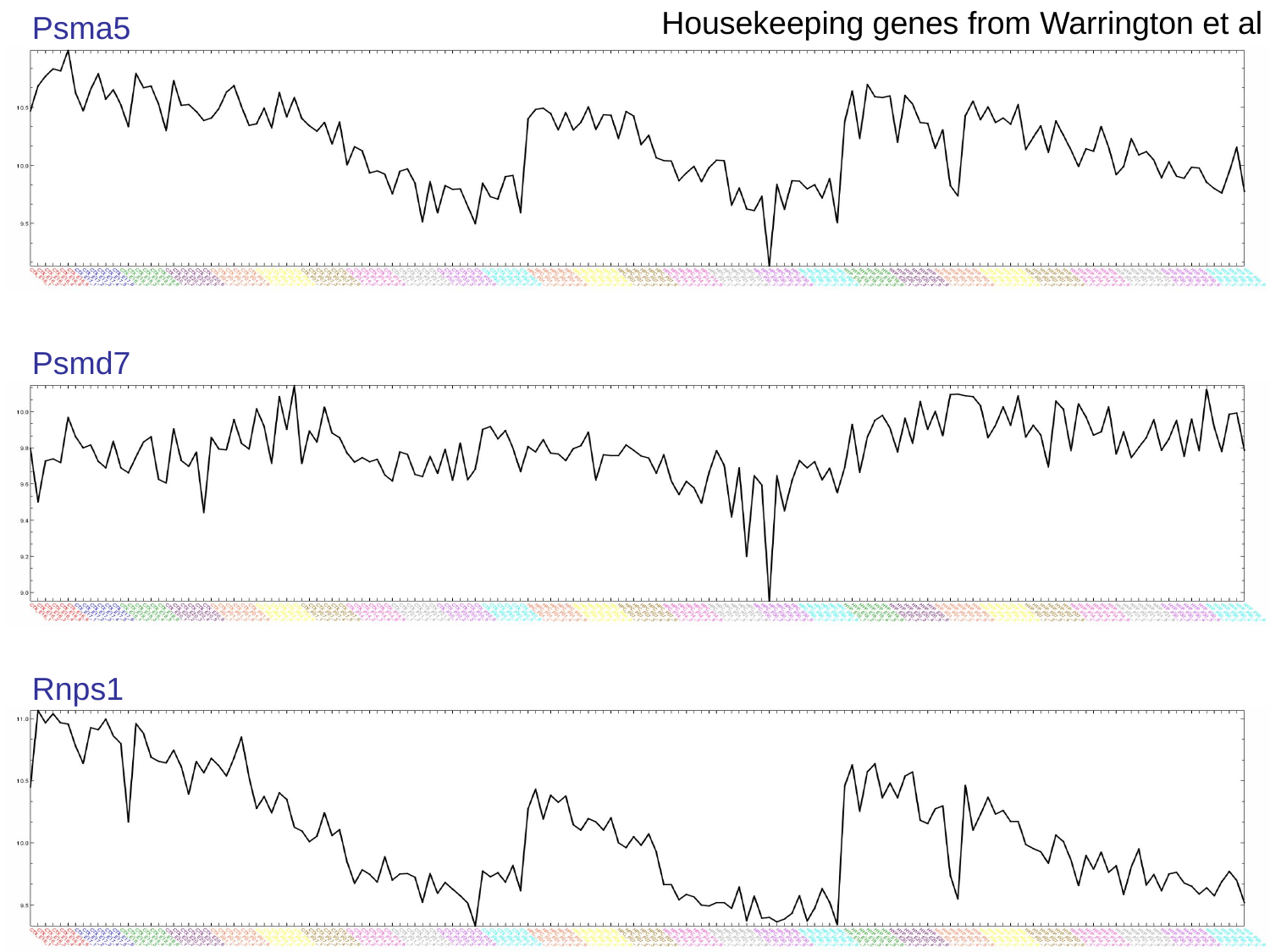

Housekeeping genes from Warrington et al
Psma5
Psmd7
Rnps1

## Slide 24
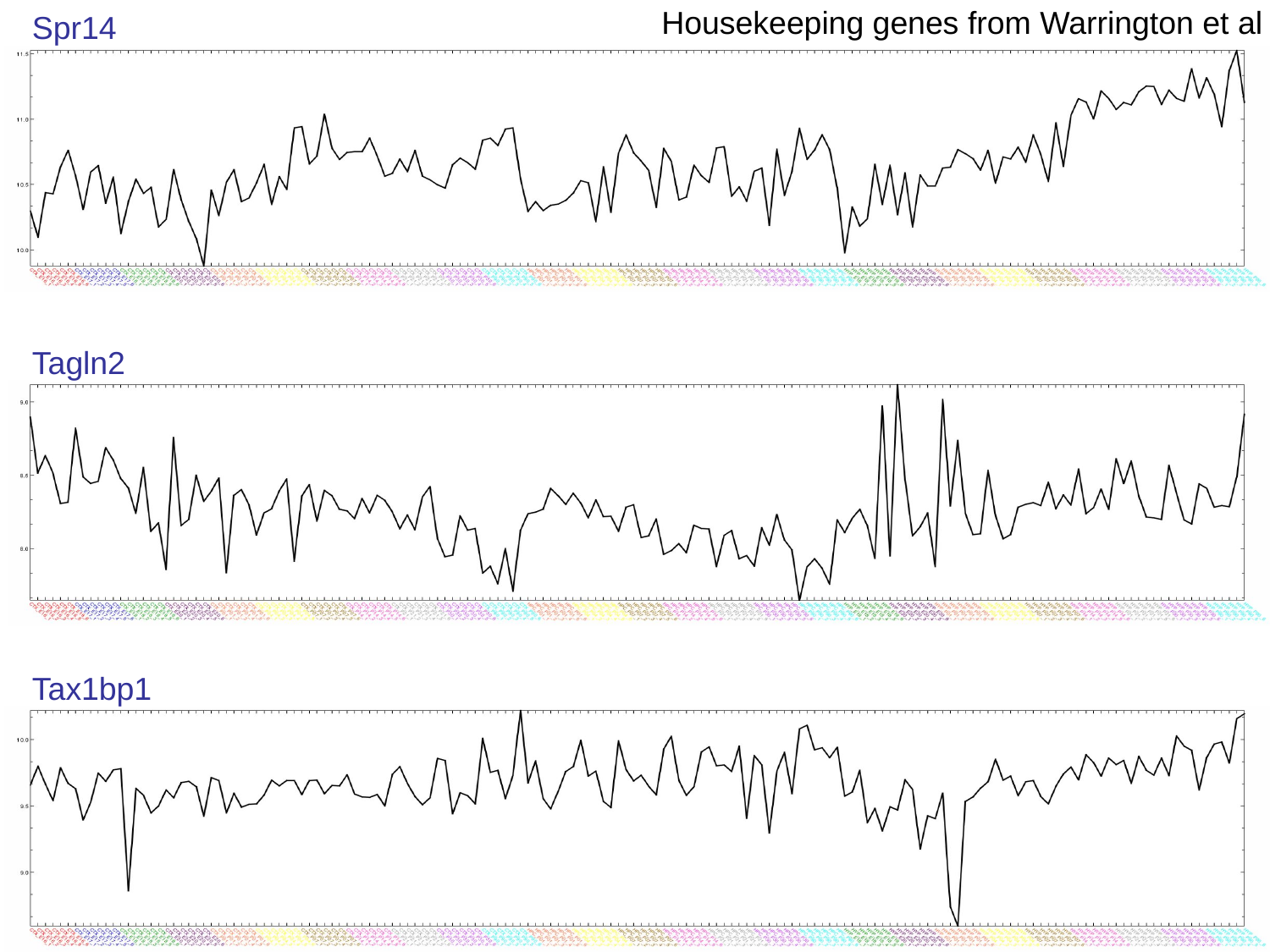

Housekeeping genes from Warrington et al
Spr14
Tagln2
Tax1bp1

## Slide 25
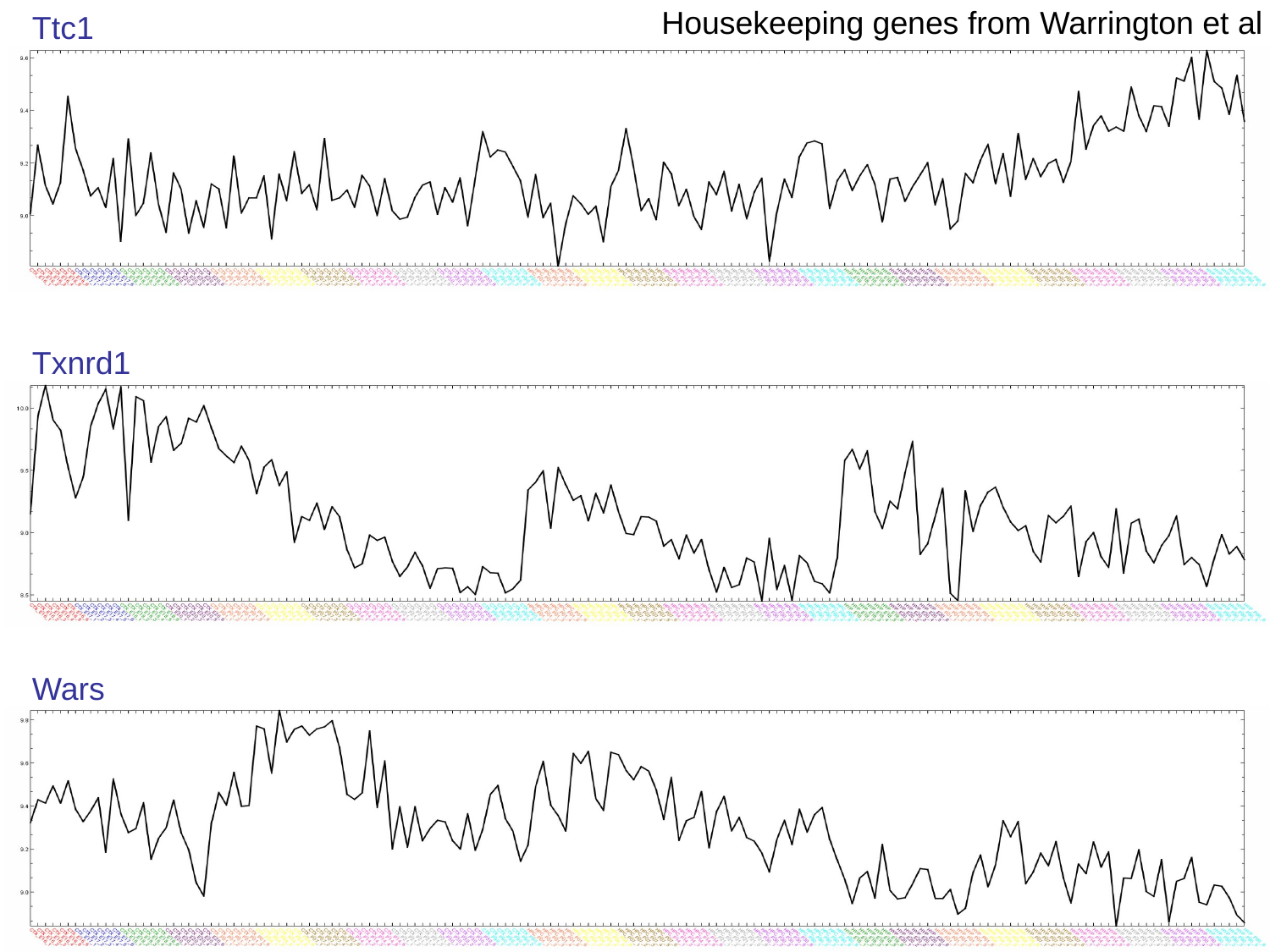

Housekeeping genes from Warrington et al
Ttc1
Txnrd1
Wars
