# Supplementary material for: Gene expression in the rat brain: High similarity but unique differences between frontomedial-, temporal- and occipital cortex
Source: BMC Neurosci. 2011 Jan 26;12:15. doi: 10.1186/1471-2202-12-15 (PMC3040714; doi:10.1186/1471-2202-12-15)

FMCx-enriched genes

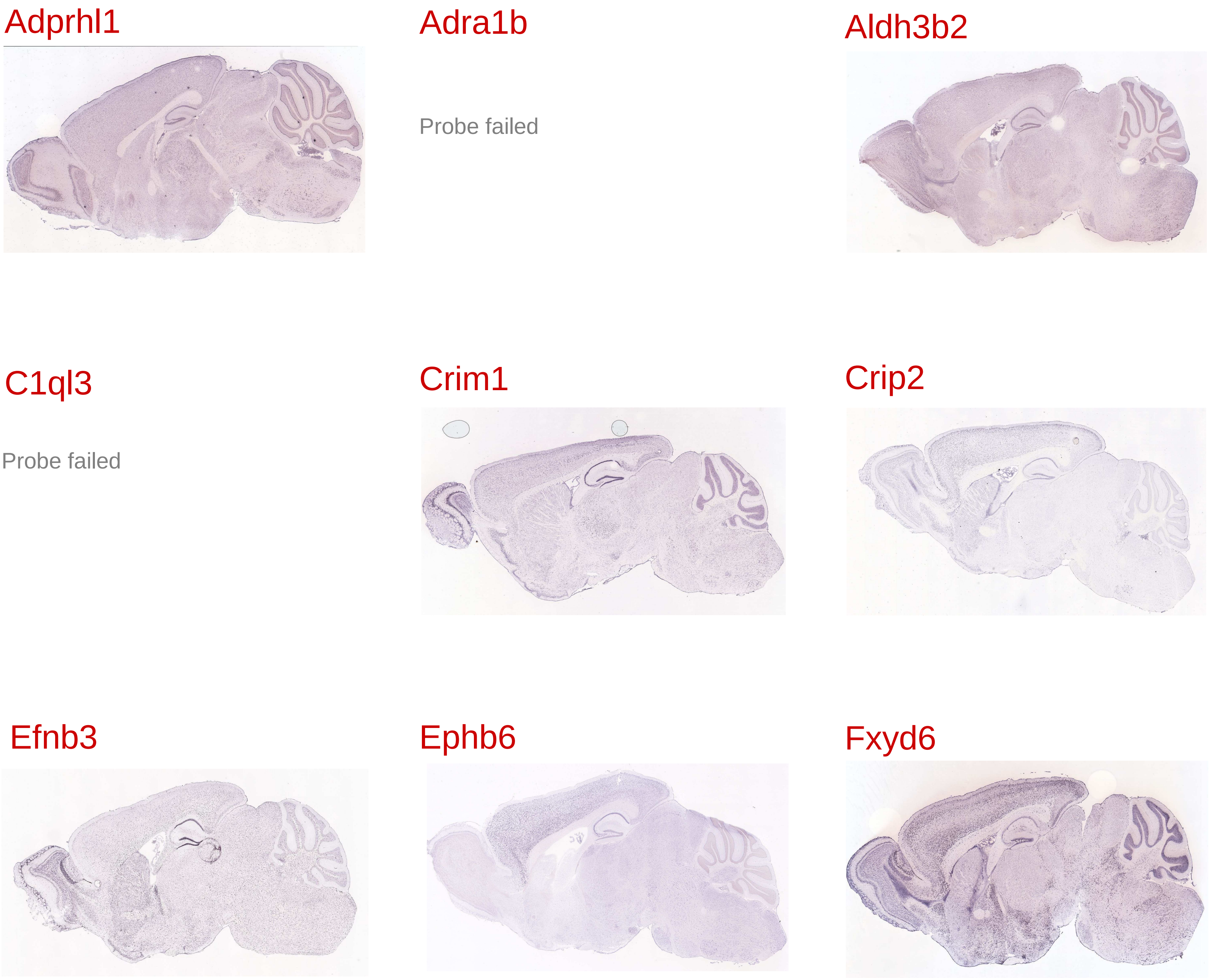

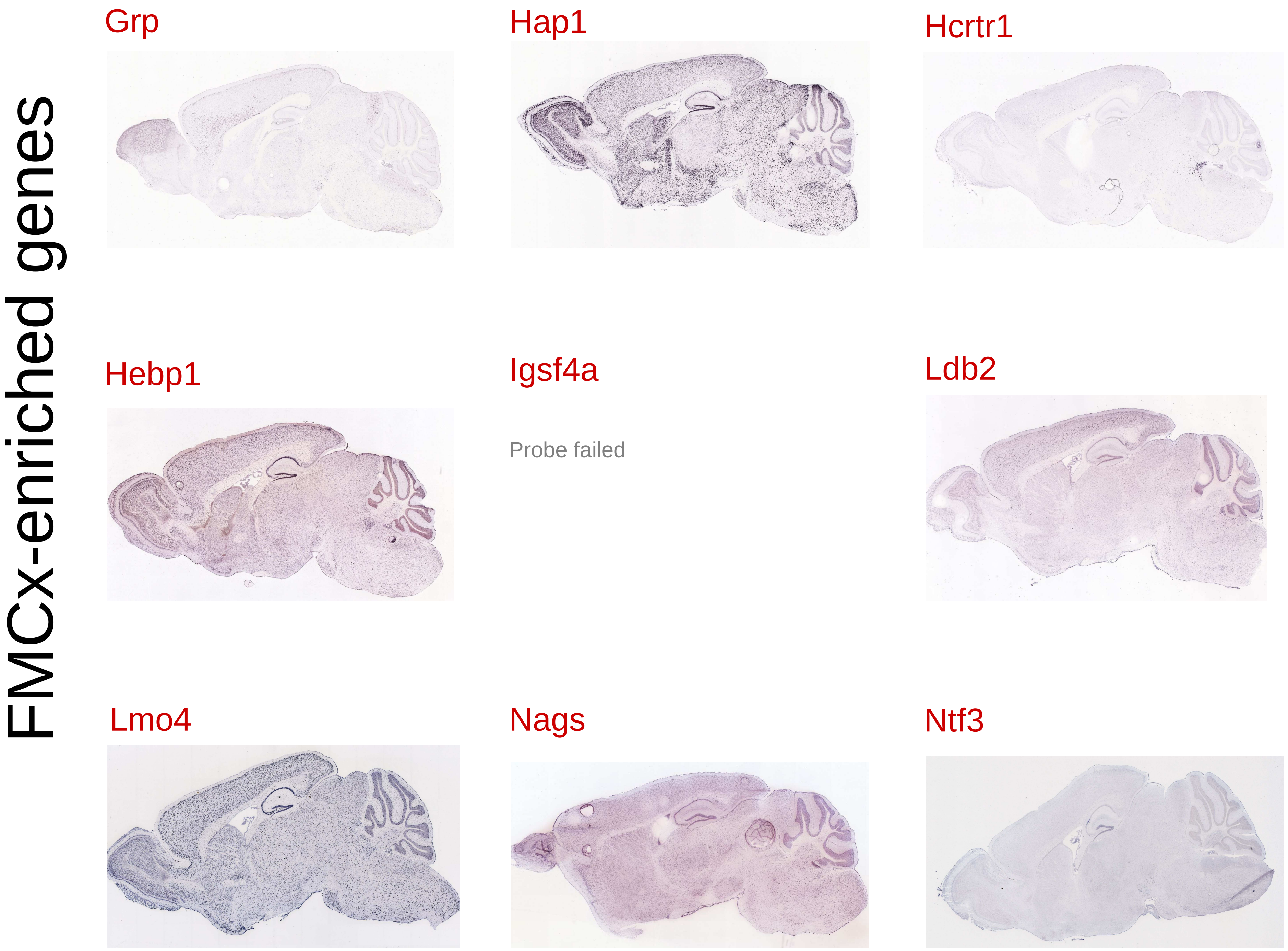
FMCx-enriched genes
Grp
Hap1
Hcrtr1
Hebp1
Igsf4a
Probe failed
Ldb2
Lmo4
Nags
Ntf3

FMCx-enriched genes

Panx1

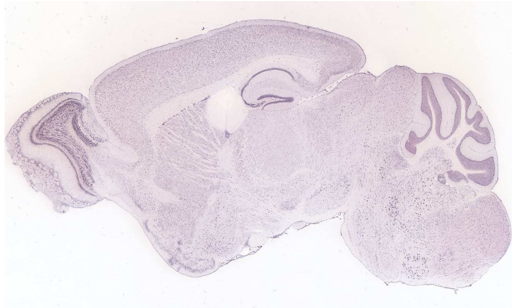

Pcdh17

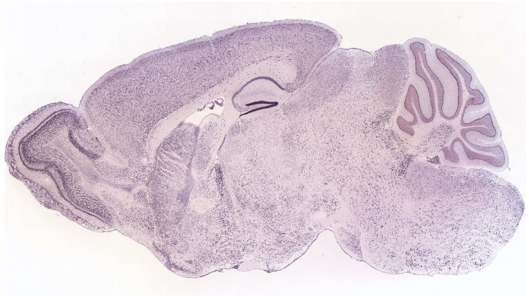

Pfkl

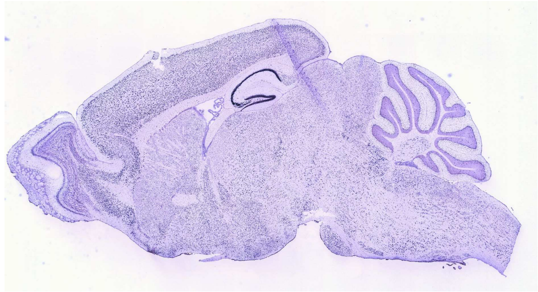

Prkcdbp

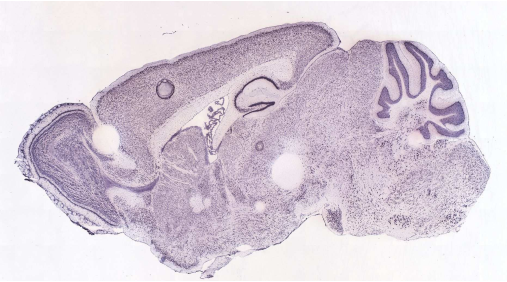

Prmt2

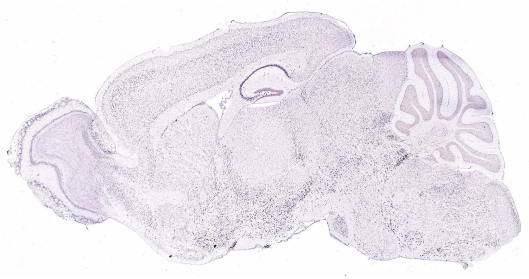

rCG46329

Not available

Rspo2

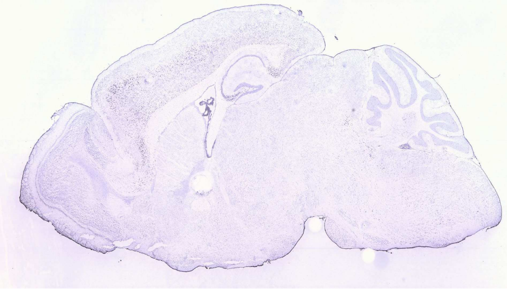

Ryr1

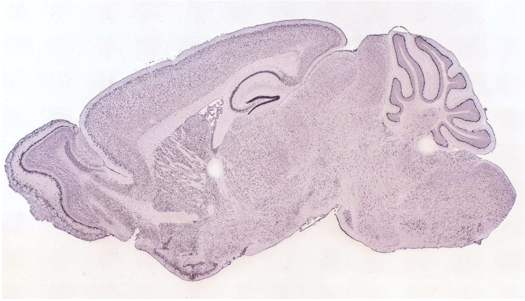

St6galnac5 (Siat7e)

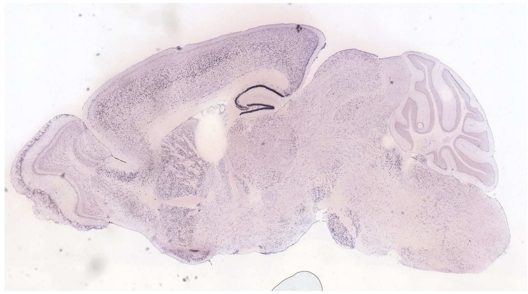

FMCx-enriched genes

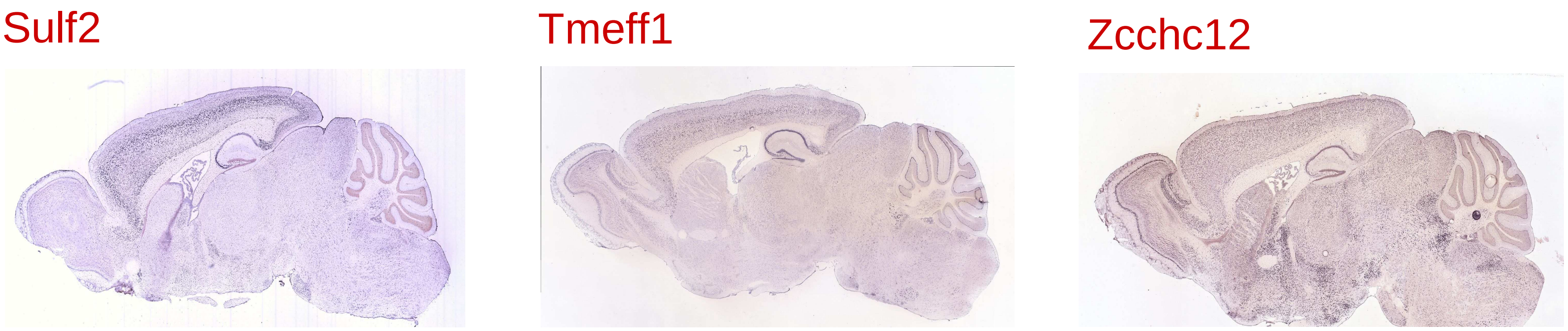

TCx-enriched genes

Arhgap9

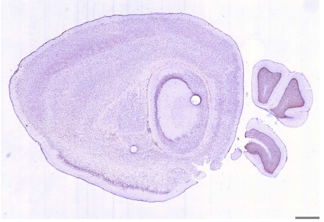

Coronal not available

Atoh7

Probe failed

Ca4

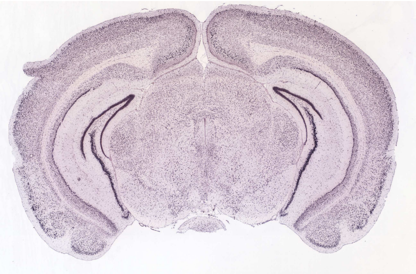

Cabp1

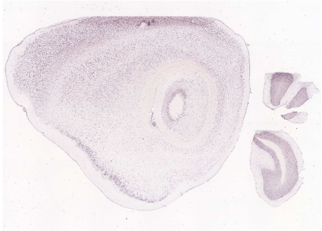

Coronal not available

Cadps2

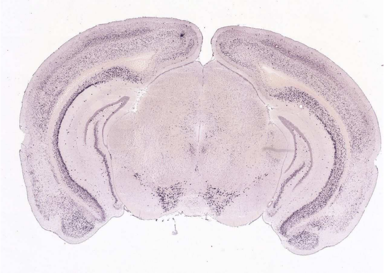

Clec2l

Probe failed

Col13a1

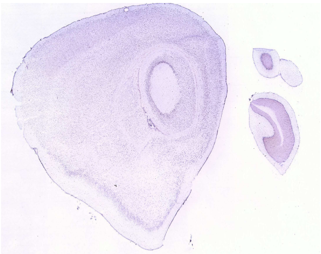

Coronal not available

Gpr88

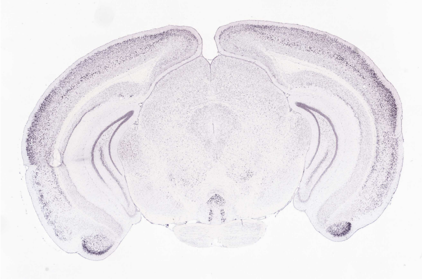

Hhatl

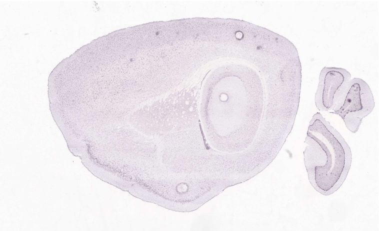

Coronal not available

TCx-enriched genes

Ikbke

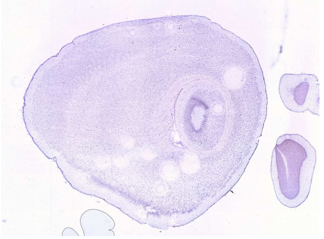

Coronal not available

Jundp2

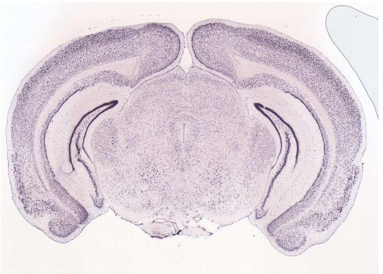

Kcnc1

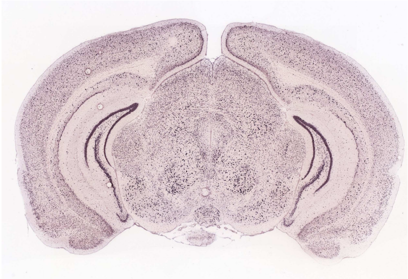

Kcns1

Probe failed

6330514A18Rik (LOC314627)

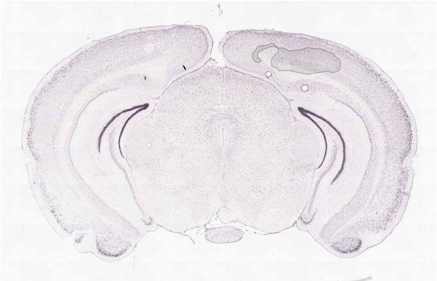

Lphn2

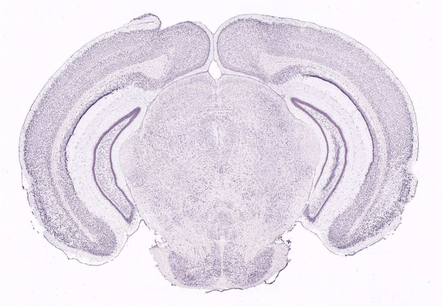

Lxn

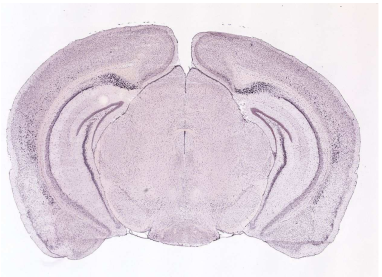

Mox2r

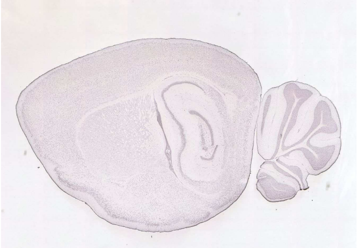

Coronal not available

Nef3

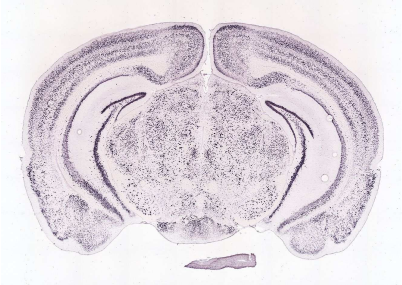

TCx-enriched genes

Neu2

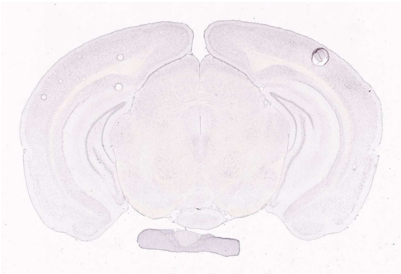

rCG41008

Not available

RGD1306921

Not available

Rorb

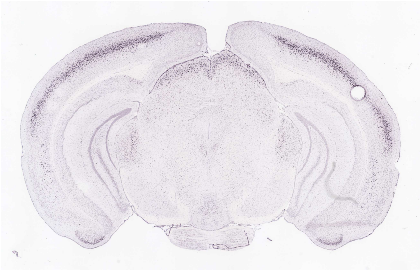

Scn1a

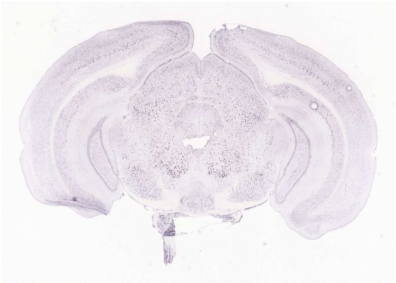

Scn4b

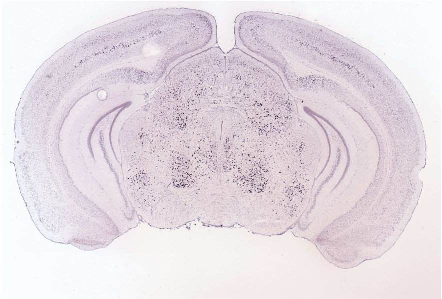

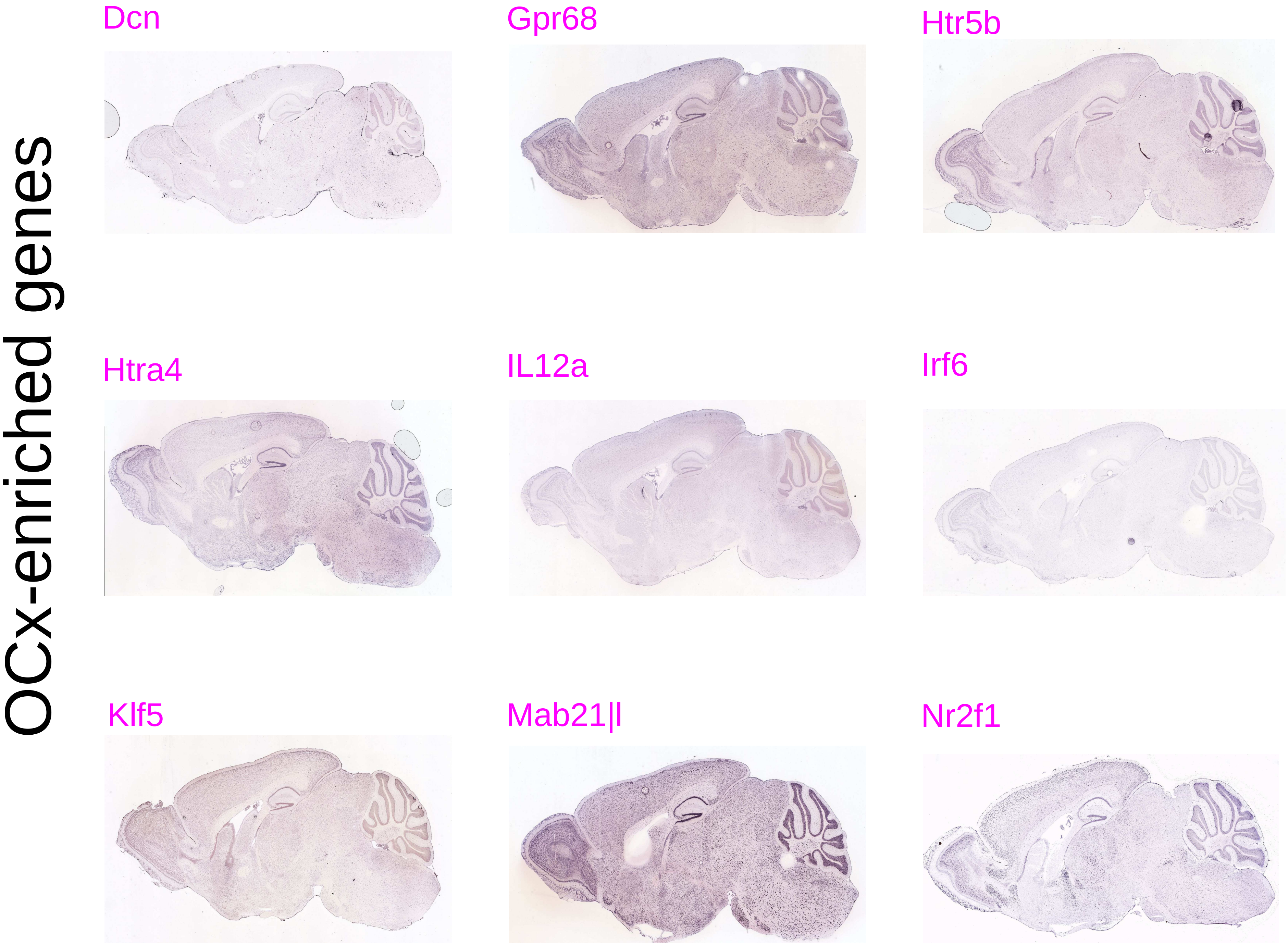
OCx-enriched genes
Dcn
Gpr68
Htr5b
Htra4
IL12a
Irf6
Klf5
Mab21|l
Nr2f1

OCx-enriched genes

Odz3

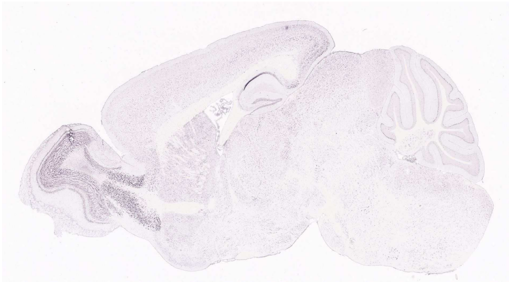

Satb1

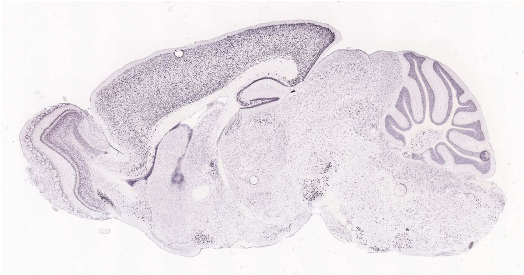

Supplement: Additional file 7 — Laminar expression profiles of regionally enriched genes. This file presents in situ hybridisation images demonstrating laminar expression patterns of the 56 regionally enriched genes that were represented in the Allen Mouse Brain Atlas. For FMCx and OCx genes, images presented in this study represent sagittal sections near midline (lateral ~0.7-1.4 mm). For TCx genes, coronal sections between Bregma -3.08 and -3.38 are presented where available. Only sagittal sections were available for Arhgap9, Cabp1, Col13a1, Hhatl, Ikbke and Mox2r, hence the lateral-most sections are presented for these genes. [file 1471-2202-12-15-S7.PDF]
