# Supplementary material for: Gene expression in the rat brain: High similarity but unique differences between frontomedial-, temporal- and occipital cortex
Source: BMC Neurosci. 2011 Jan 26;12:15. doi: 10.1186/1471-2202-12-15 (PMC3040714; doi:10.1186/1471-2202-12-15)
Supplement: Additional file 8 — Expression of regionally enriched genes in astrocytes, oligodendrocytes and neurons. This file shows gene expression profiles of regionally enriched genes across differentiating as well as mature astrocytes, oligodendrocytes and neurons. Individual samples are placed along the x-axis; Cultured Astroglia, Astrocytes P1, P7-P8 and P17, Astrocytes Gray P17, Oligodendrocyte progenitor cells, premyelinating, postmitotic oligodendrocytes (Myelin Oligos), Oligodendrocytes, Neurons P7n, P7, P16n and P16, see original publication for details. The y-axis indicates quantile normalised signal intensities for each gene in each individual sample. Raw microarray data were obtained from Cahoy et al [28]. 42 of our genes were represented in this data set. [file 1471-2202-12-15-S8.PPT]

## Slide 1
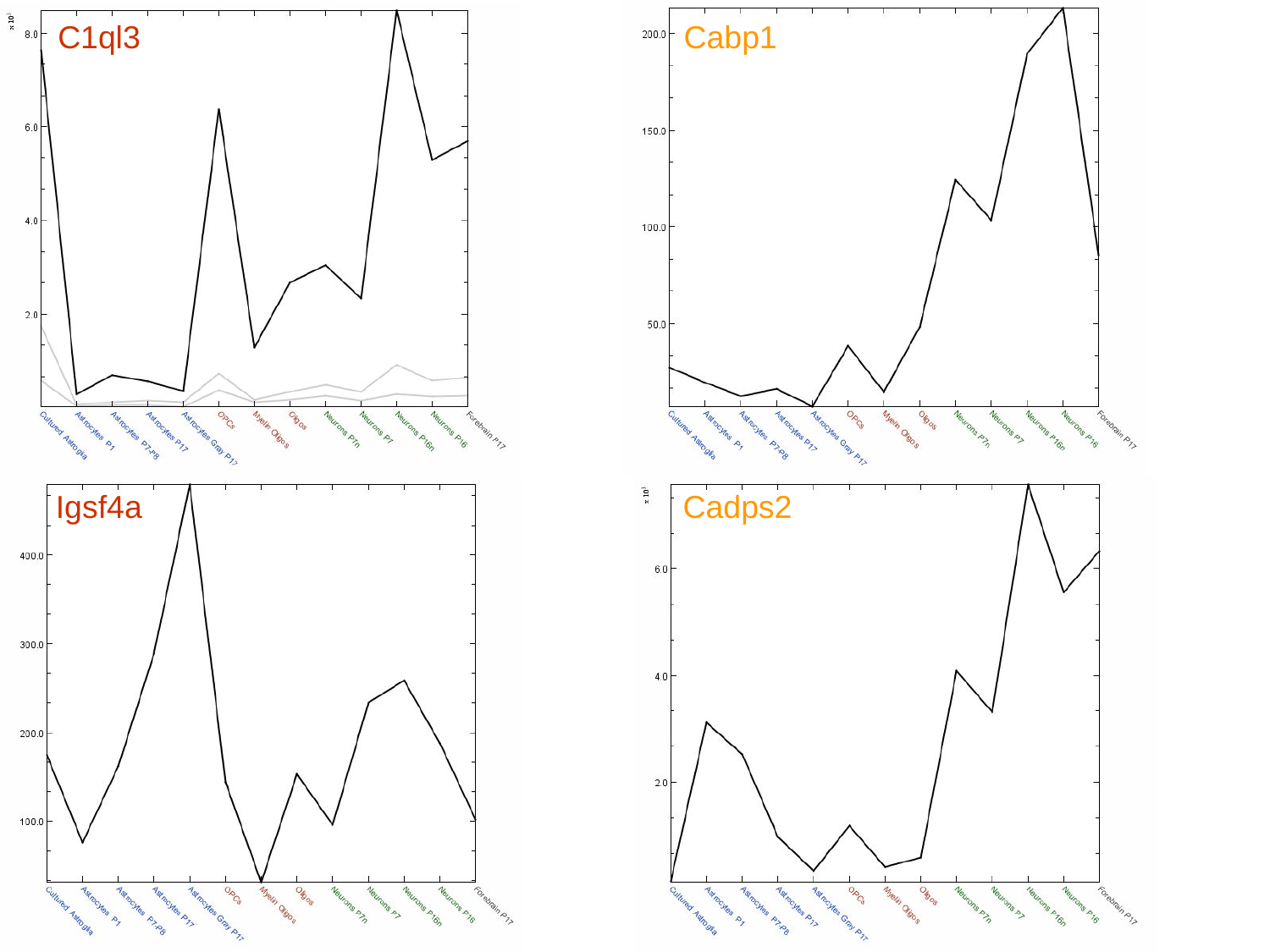

C1ql3
Cabp1
Igsf4a
Cadps2

## Slide 2
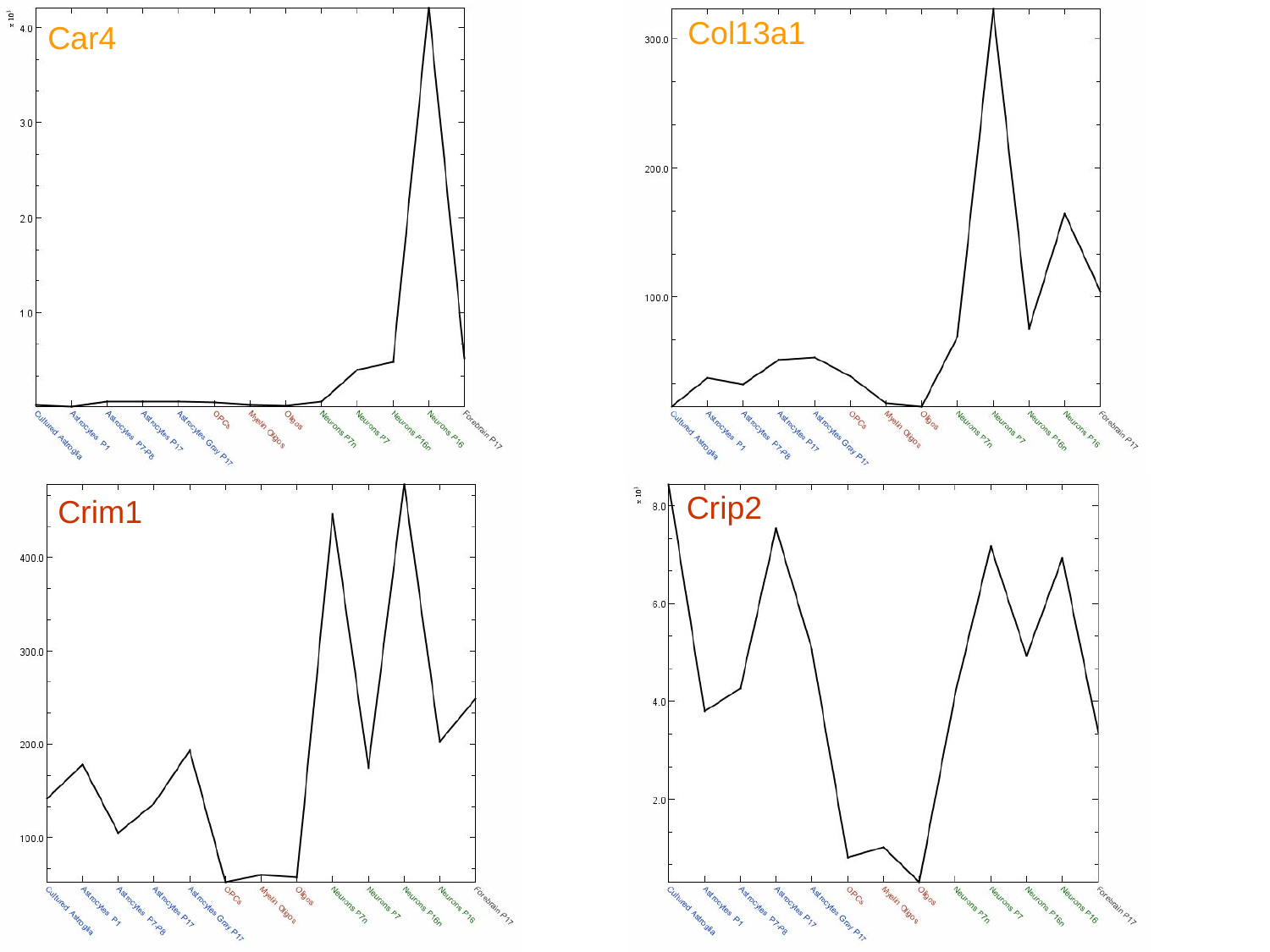

Col13a1
Car4
Crip2
Crim1

## Slide 3
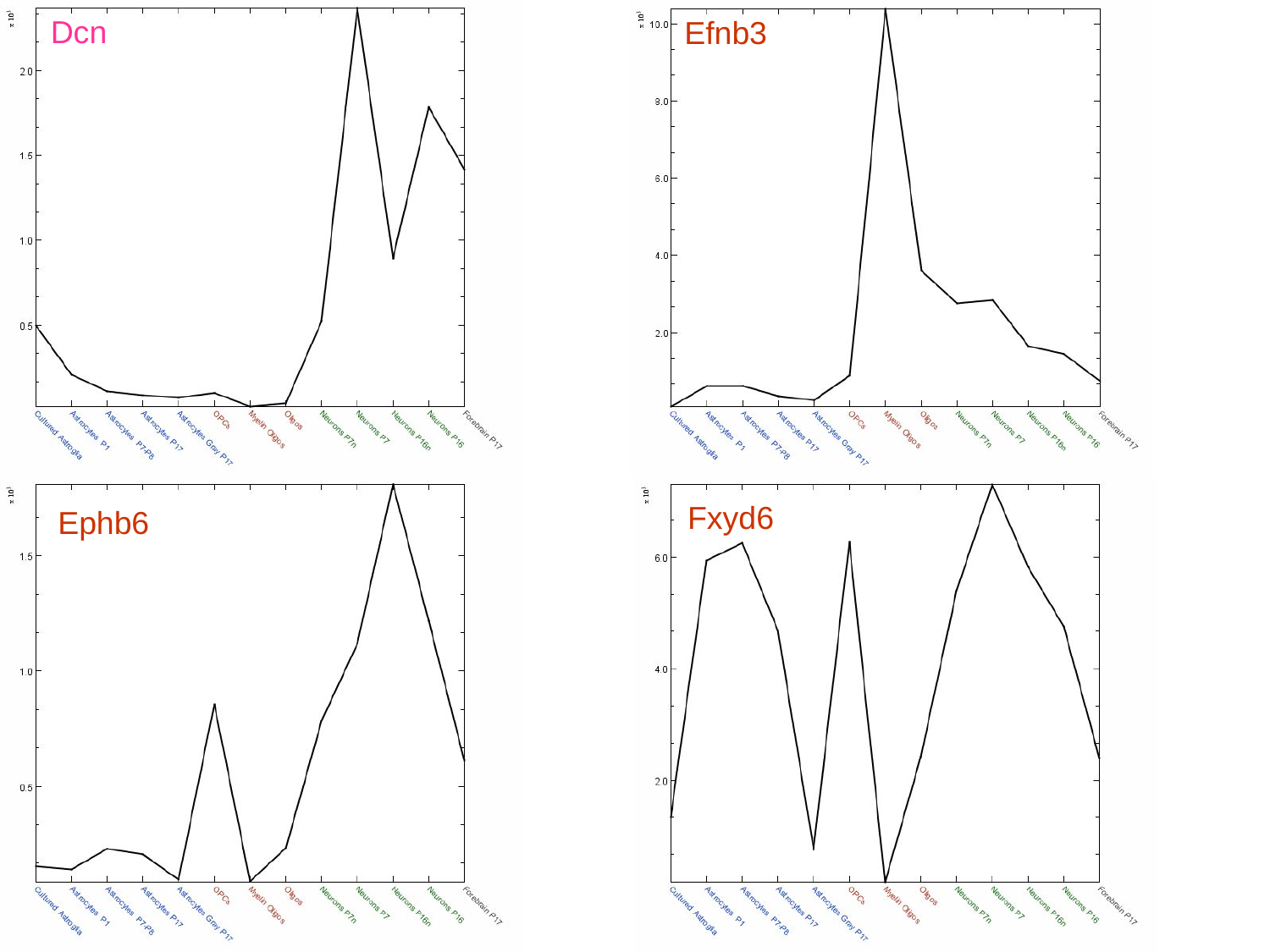

Dcn
Efnb3
Fxyd6
Ephb6

## Slide 4
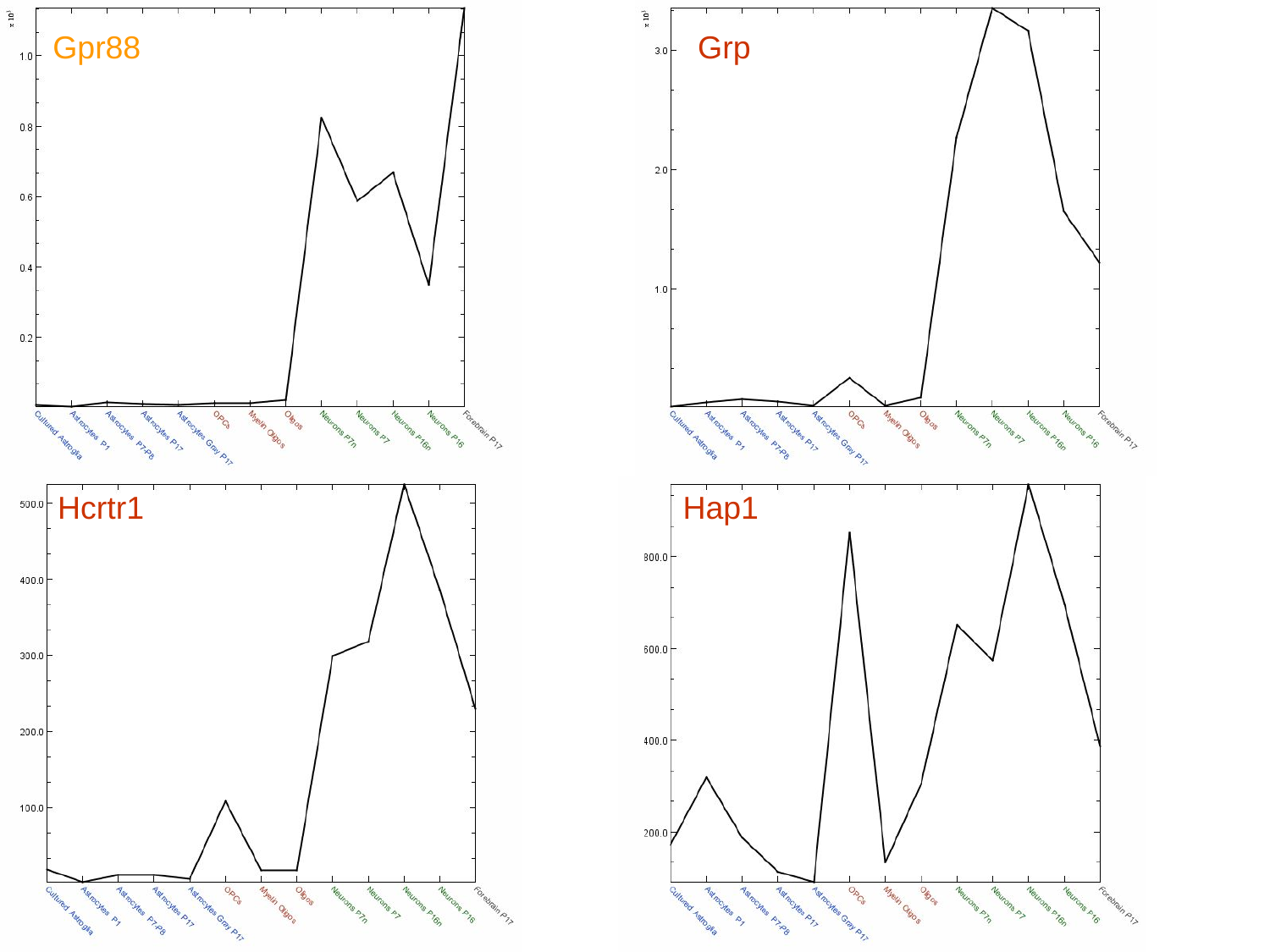

Gpr88
Grp
Hcrtr1
Hap1

## Slide 5
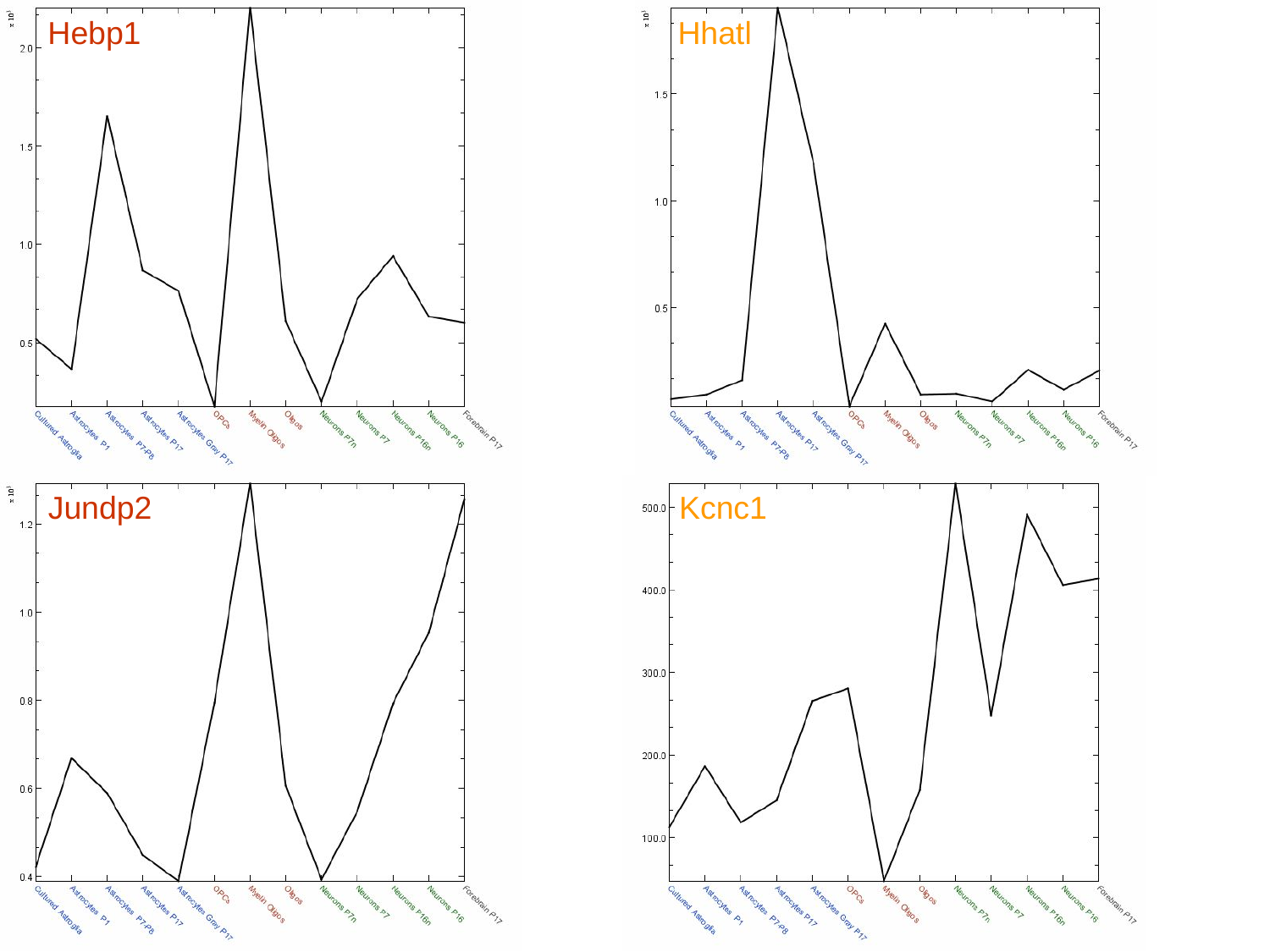

Hebp1
Hhatl
Jundp2
Kcnc1

## Slide 6
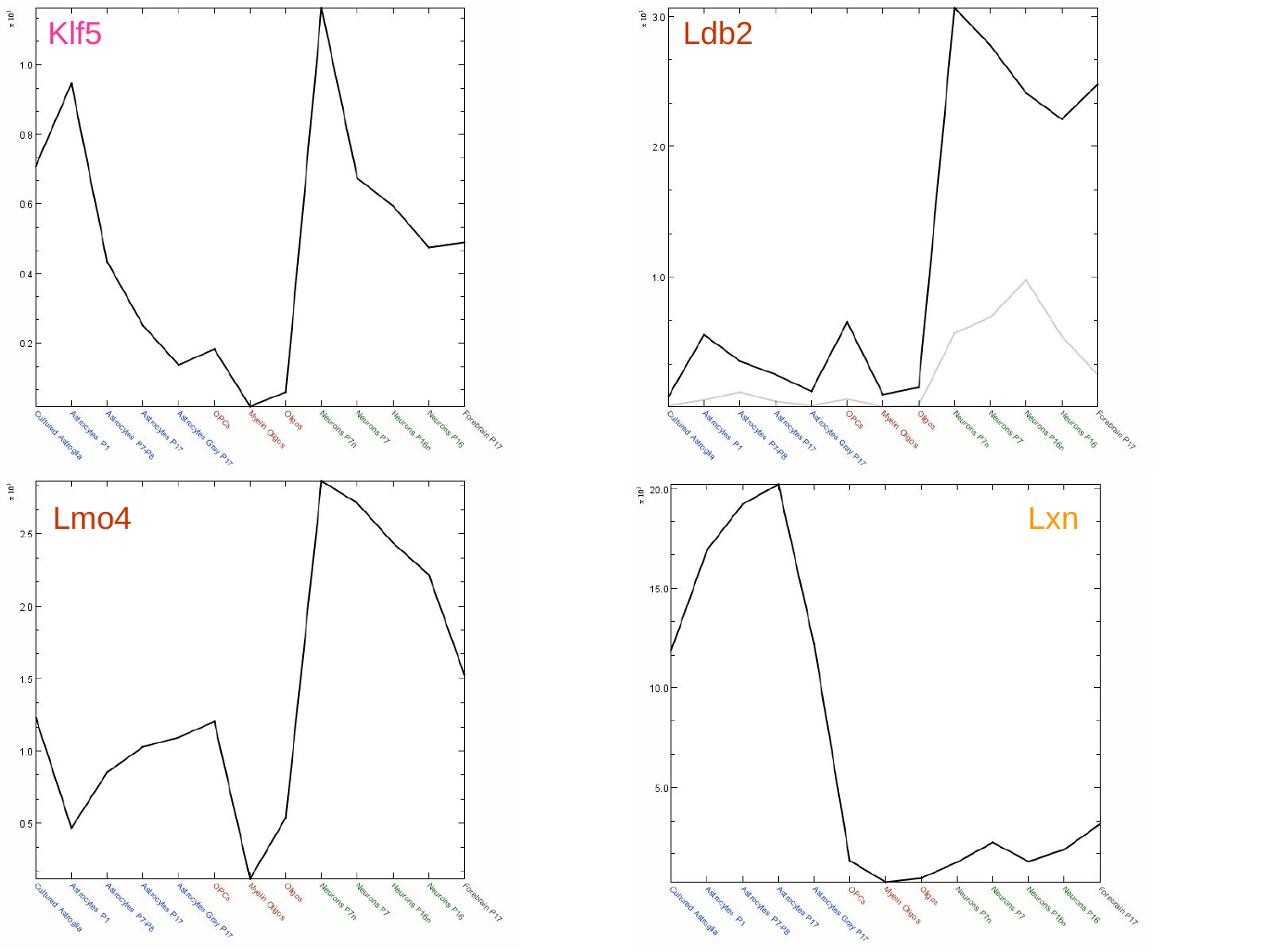

Klf5
Ldb2
Lmo4
Lxn

## Slide 7
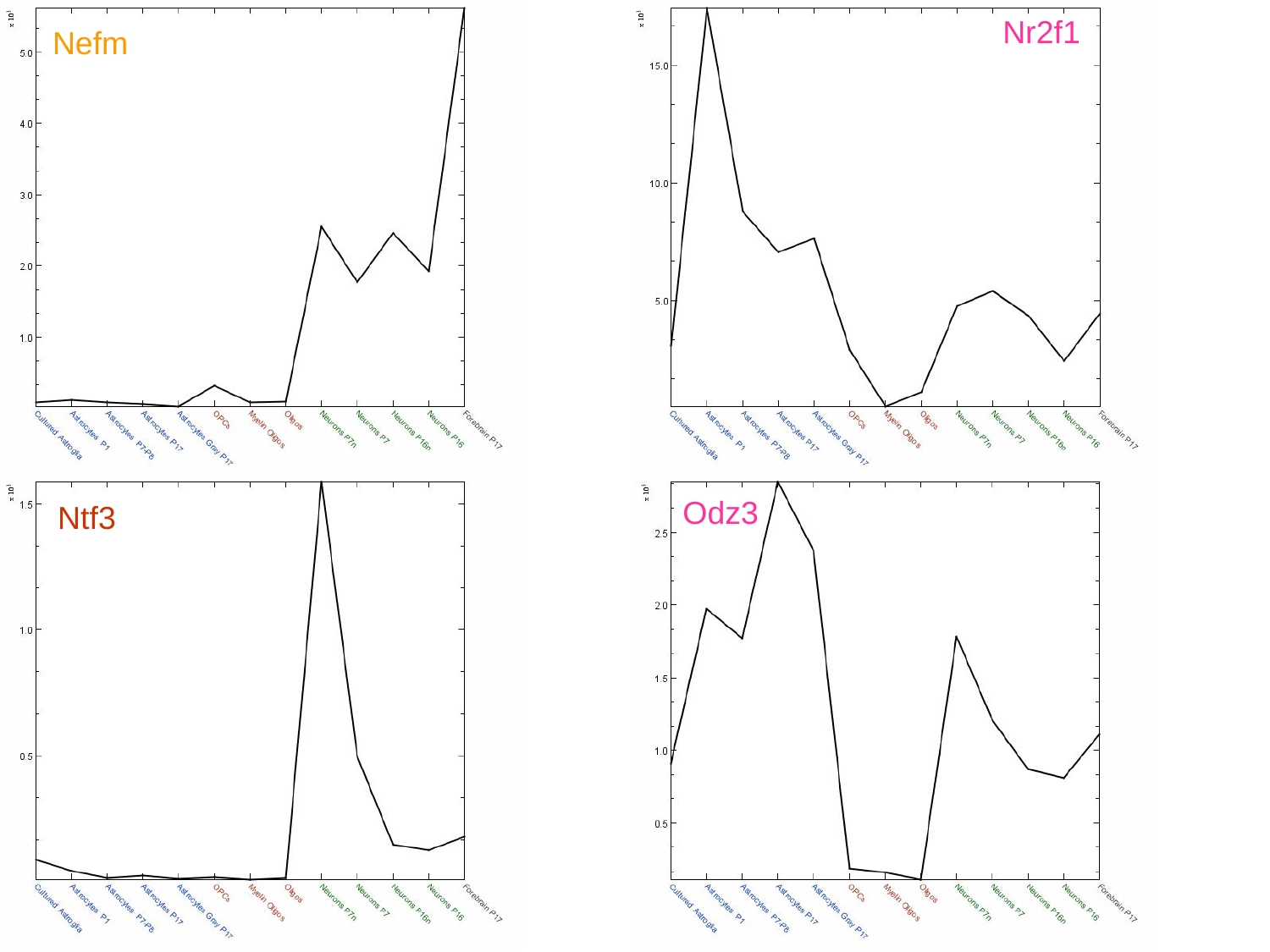

Nr2f1
Nefm
Odz3
Ntf3

## Slide 8
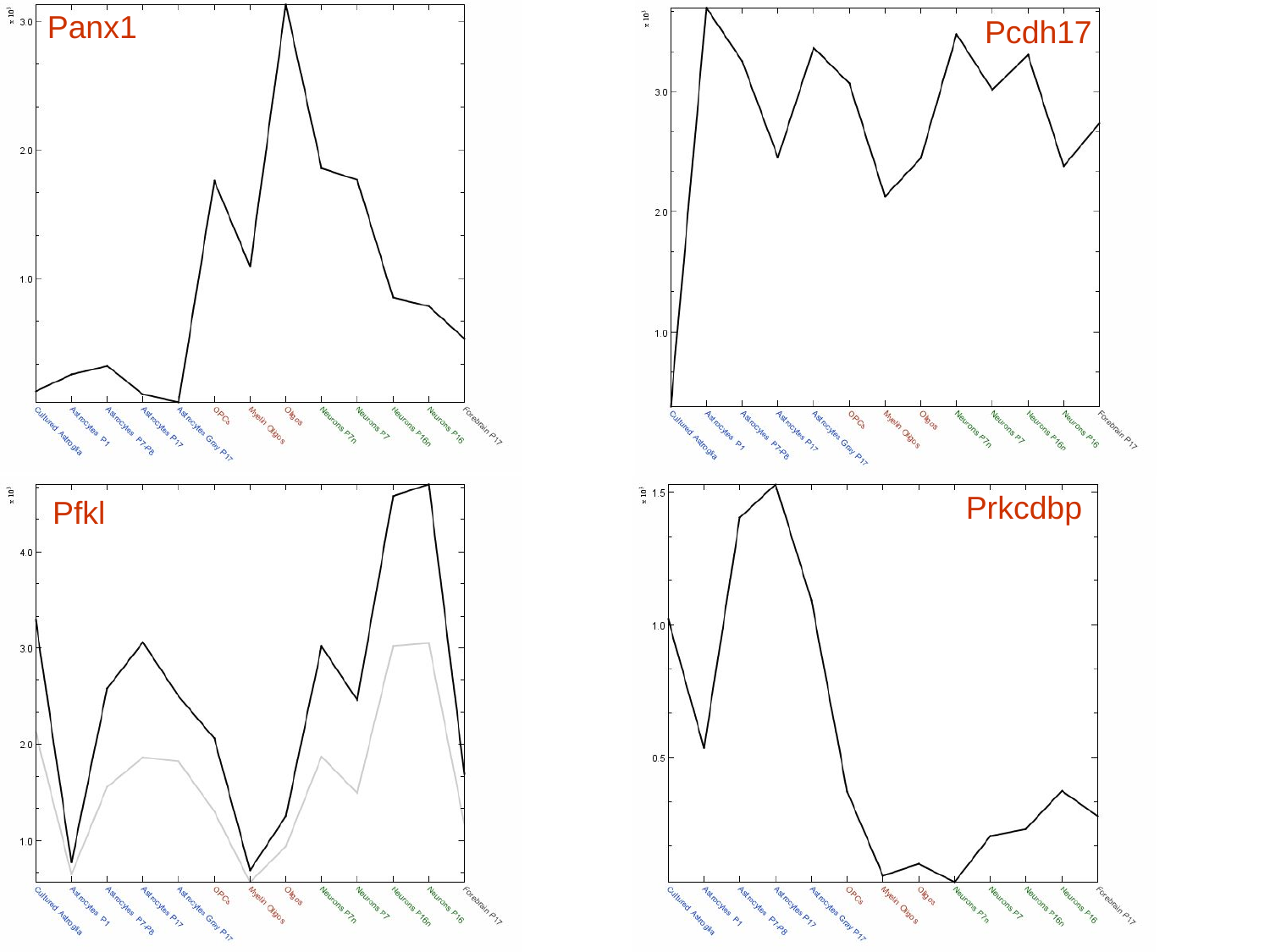

Panx1
Pcdh17
Prkcdbp
Pfkl

## Slide 9
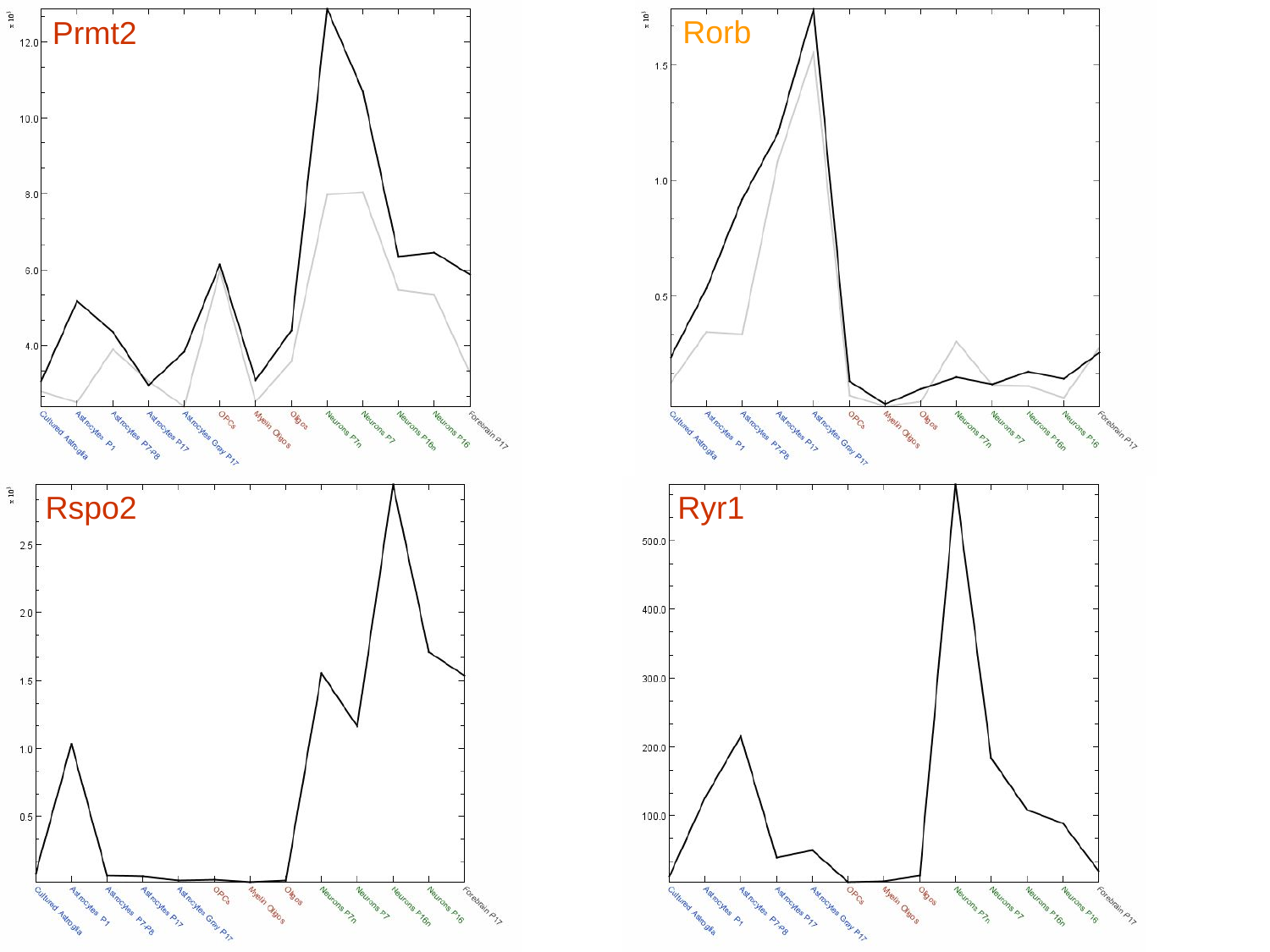

Rorb
Prmt2
Rspo2
Ryr1

## Slide 10
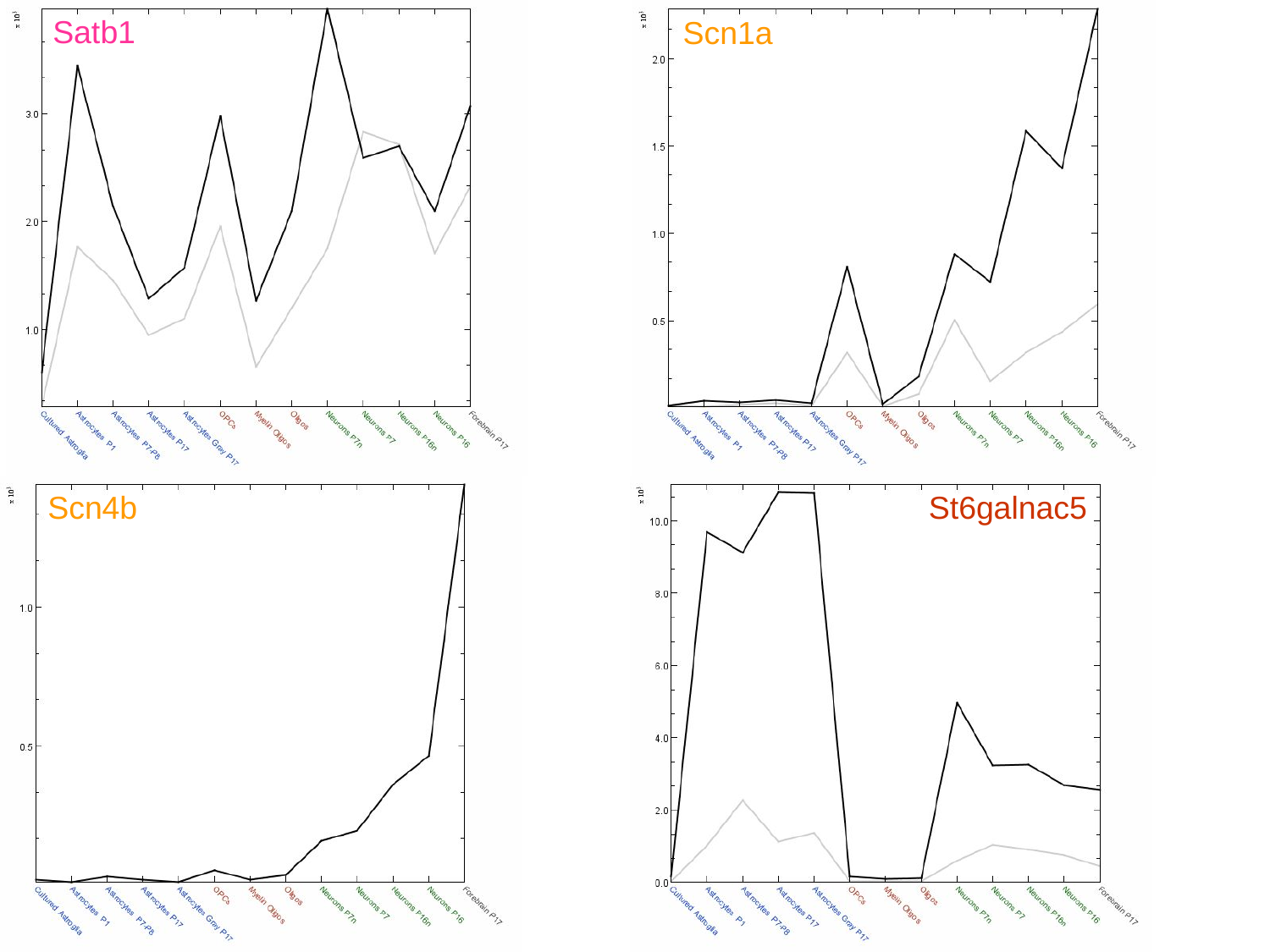

Satb1
Scn1a
Scn4b
St6galnac5

## Slide 11
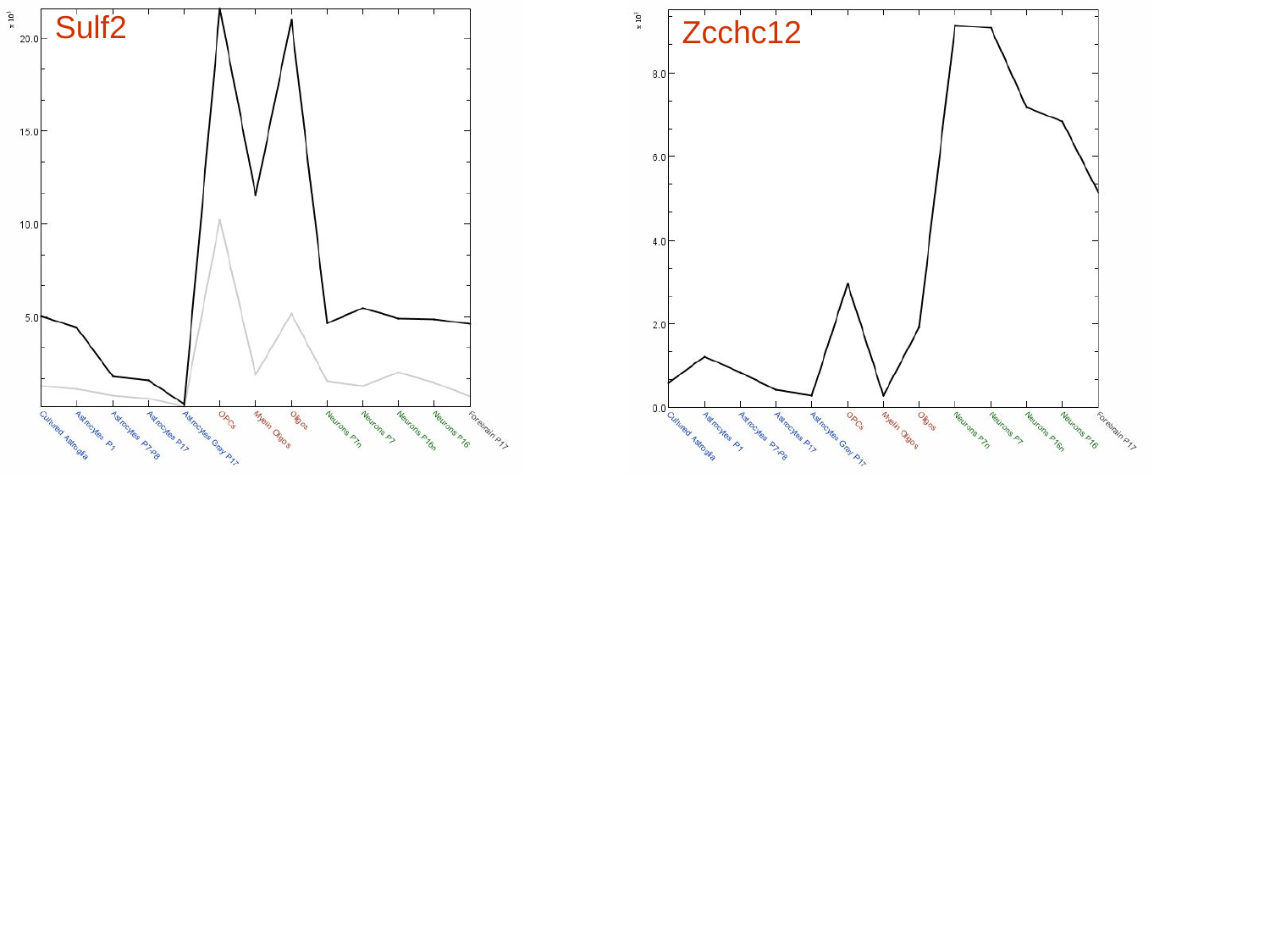

Sulf2
Zcchc12
