# Supplementary material for: Gene expression in the rat brain: High similarity but unique differences between frontomedial-, temporal- and occipital cortex
Source: BMC Neurosci. 2011 Jan 26;12:15. doi: 10.1186/1471-2202-12-15 (PMC3040714; doi:10.1186/1471-2202-12-15)
Supplement: Additional file 9 — Expression of regionally enriched genes in different neuronal subtypes. This file shows gene expression profiles of regionally enriched genes across different subtypes of neocortical neurons. Individual samples are placed along the x-axis; layer 6 glutamatergic neurons from cingulate cortex (CT6 strain), layer 5 glutamatergic neurons from cingulate cortex (YFPH strain), layer 5-6 glutamatergic neurons from somatosensory cortex (YFPH strain), layer 1-6 GABAergic neurons from cingulate cortex (G30 strain), layer 4-6 GABAergic neurons from cingulate cortex (G43 strain), layer 2-4 GABAergic neurons from cingulate cortex (GIN strain) and layer 1-6 GABAergic neurons from somatosensory cortex (G30 strain); see original publication for details. The y-axis indicates quantile normalised signal intensities for each gene in each individual sample. Raw microarray data were obtained from Sugino et al [30]. 51 of our genes were represented in this data set. [file 1471-2202-12-15-S9.PPT]

## Slide 1
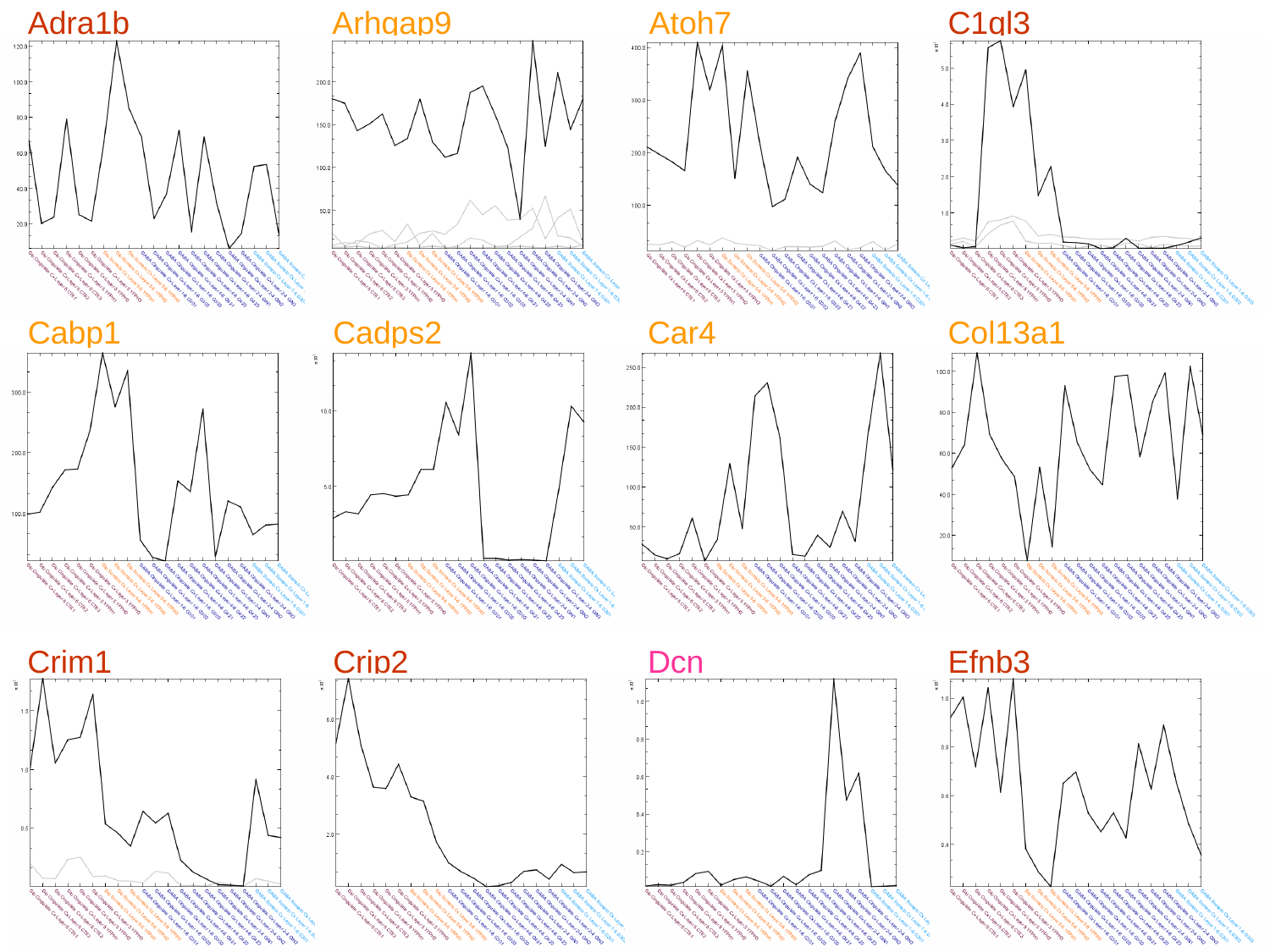

Adra1b
Arhgap9
Atoh7
C1ql3
Cabp1
Cadps2
Car4
Col13a1
Crim1
Crip2
Dcn
Efnb3

## Slide 2
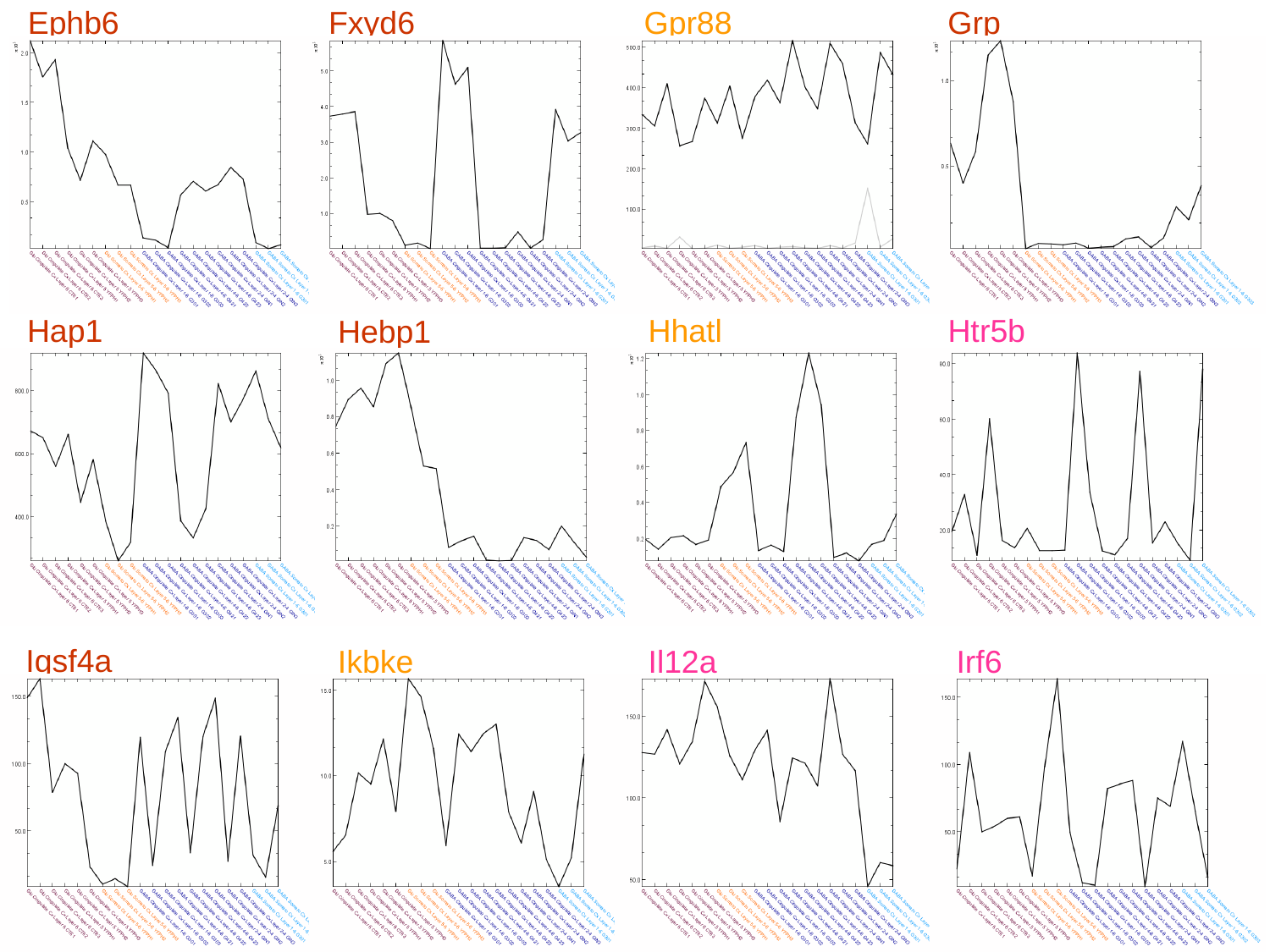

Ephb6
Fxyd6
Gpr88
Grp
Hap1
Hhatl
Htr5b
Hebp1
Igsf4a
Ikbke
Il12a
Irf6

## Slide 3
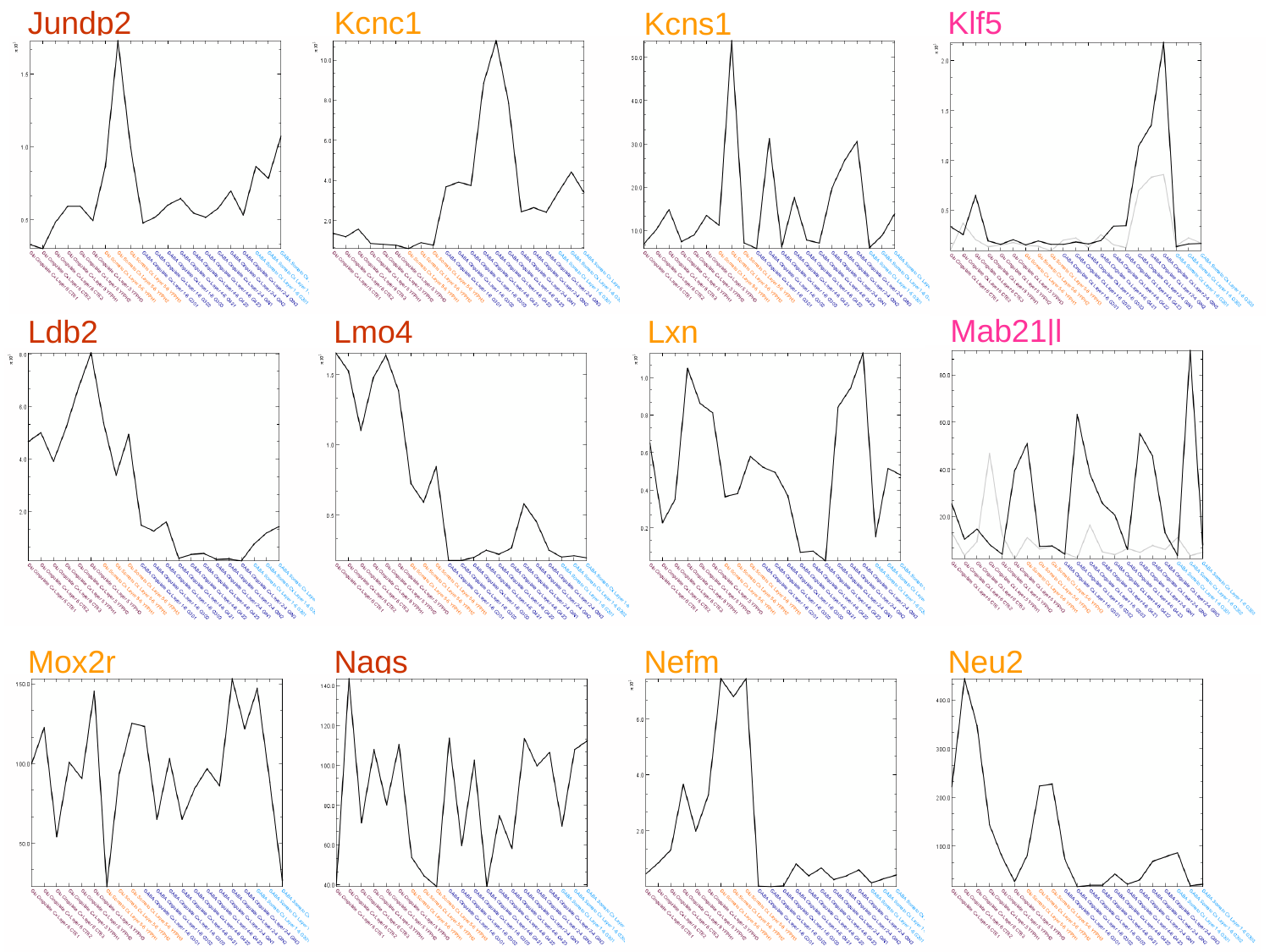

Jundp2
Kcnc1
Klf5
Kcns1
Mab21|l
Ldb2
Lmo4
Lxn
Mox2r
Nags
Nefm
Neu2

## Slide 4
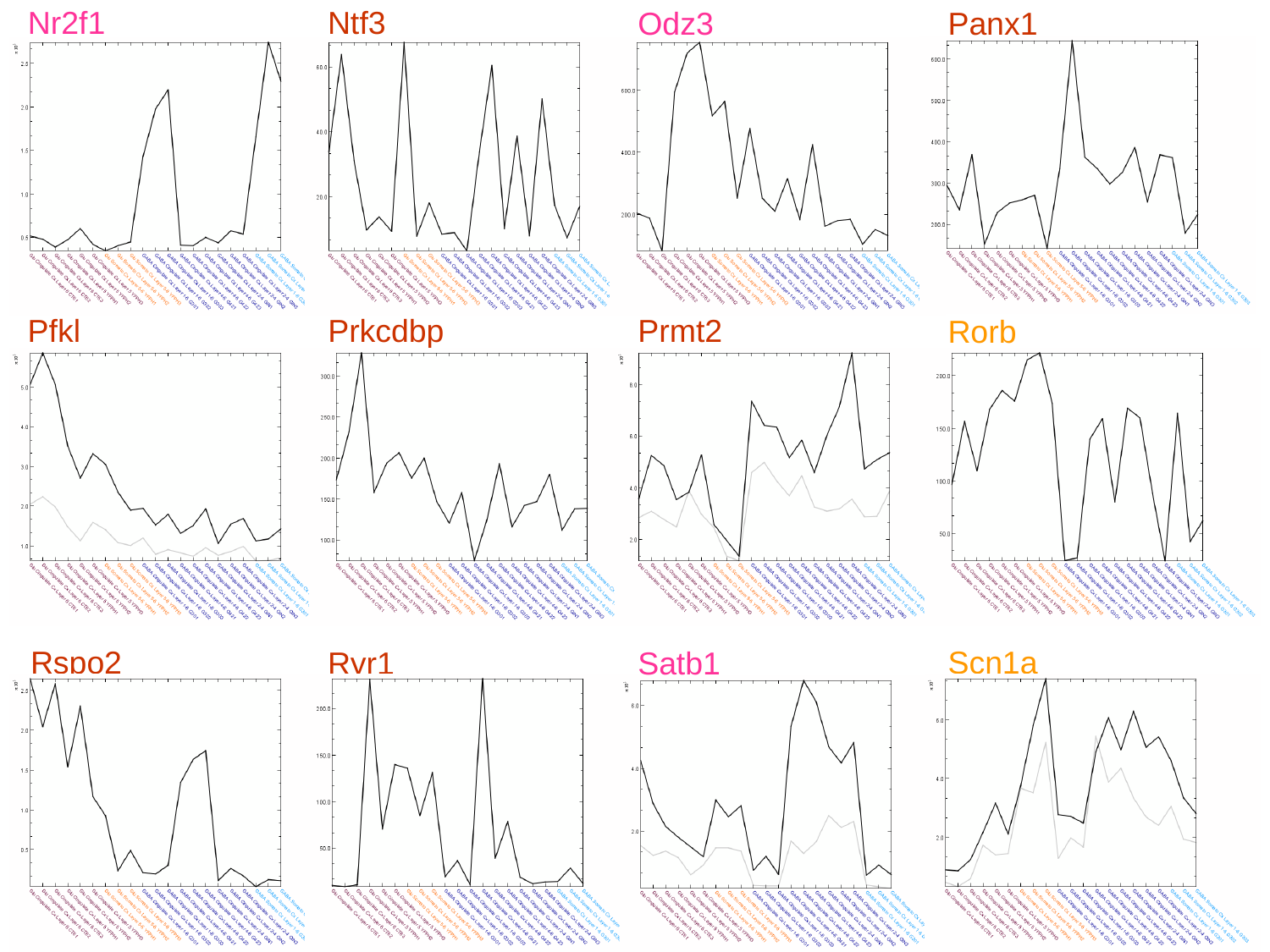

Nr2f1
Ntf3
Odz3
Panx1
Pfkl
Prkcdbp
Prmt2
Rorb
Rspo2
Scn1a
Ryr1
Satb1

## Slide 5
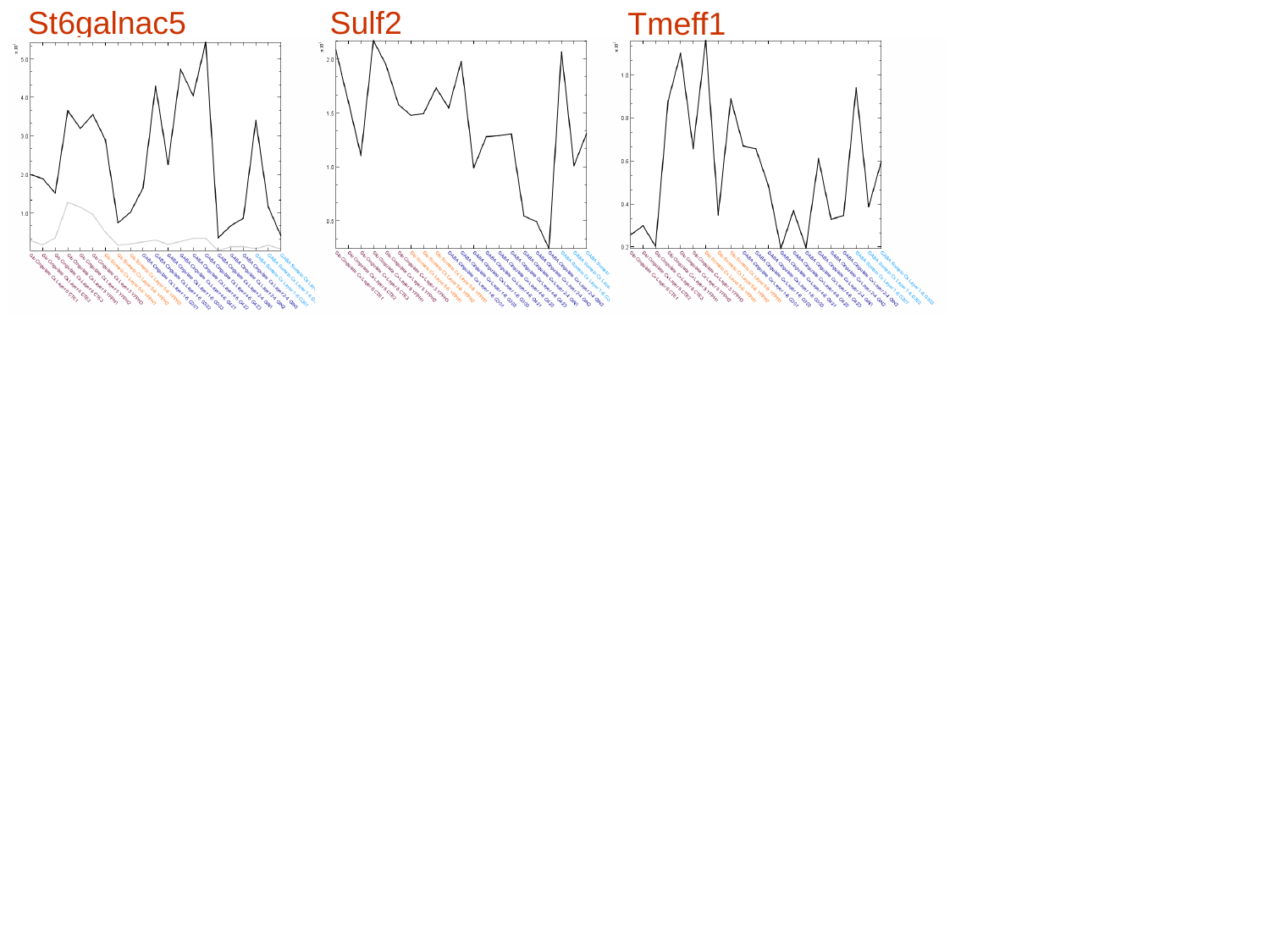

St6galnac5
Sulf2
Tmeff1
